# Solar‐Driven Biomass Reforming for Hydrogen Generation: Principles, Advances, and Challenges

**DOI:** 10.1002/advs.202402651

**Published:** 2024-05-30

**Authors:** Hu Pan, Jinglin Li, Yangang Wang, Qineng Xia, Liang Qiu, Baowen Zhou

**Affiliations:** ^1^ College of Biological Chemical Science and Engineering Jiaxing University 899 Guangqiong Road Jiaxing Zhejiang 314001 China; ^2^ Key Laboratory for Power Machinery and Engineering of Ministry of Education Research Center for Renewable Synthetic Fuel School of Mechanical Engineering Shanghai Jiao Tong University 800 Dongchuan Road Shanghai 200240 China

**Keywords:** biomass reforming, hydrogen generation, photo(thermal) catalysis, photocatalysts, semiconductors

## Abstract

Hydrogen (H_2_) has emerged as a clean and versatile energy carrier to power a carbon‐neutral economy for the post‐fossil era. Hydrogen generation from low‐cost and renewable biomass by virtually inexhaustible solar energy presents an innovative strategy to process organic solid waste, combat the energy crisis, and achieve carbon neutrality. Herein, the progress and breakthroughs in solar‐powered H_2_ production from biomass are reviewed. The basic principles of solar‐driven H_2_ generation from biomass are first introduced for a better understanding of the reaction mechanism. Next, the merits and shortcomings of various semiconductors and cocatalysts are summarized, and the strategies for addressing the related issues are also elaborated. Then, various bio‐based feedstocks for solar‐driven H_2_ production are reviewed with an emphasis on the effect of photocatalysts and catalytic systems on performance. Of note, the concurrent generation of value‐added chemicals from biomass reforming is emphasized as well. Meanwhile, the emerging photo‐thermal coupling strategy that shows a grand prospect for maximally utilizing the entire solar energy spectrum is also discussed. Further, the direct utilization of hydrogen from biomass as a green reductant for producing value‐added chemicals via organic reactions is also highlighted. Finally, the challenges and perspectives of photoreforming biomass toward hydrogen are envisioned.

## Graphical abstract

The progress and breakthroughs in semiconductor‐based photocatalysts for hydrogen generation from solar‐driven reforming of biomass and its derivatives are reviewed.

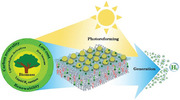

## 1 Introduction

**Figure 1:**
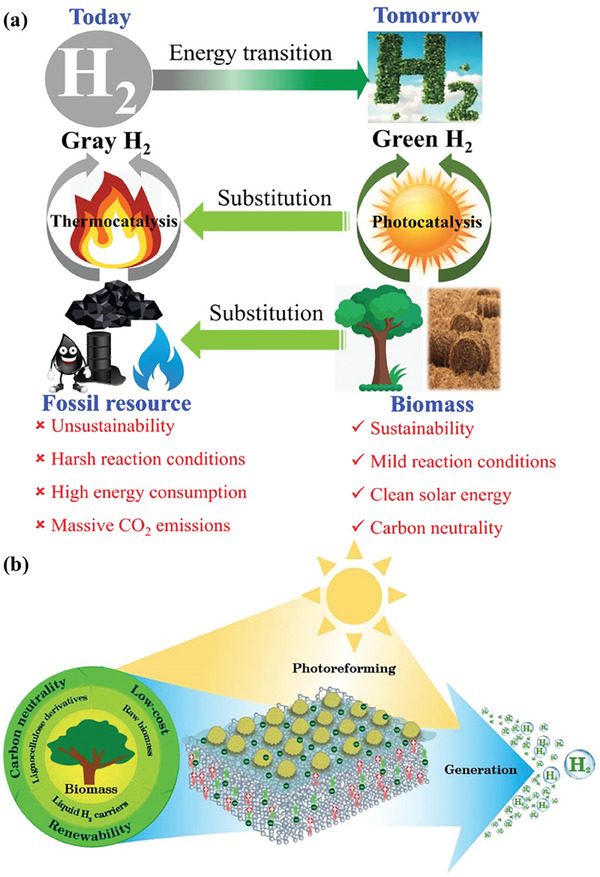
a) Comparison between gray hydrogen from fossil fuels and green hydrogen from sunlight and biomass. b) Schematic illustration of biomass photoreforming to H_2_ over semiconductor‐based photocatalysts in this review.

Energy is one kind of basic resource for the survival and development of our humanity. At present, more than 80% of global energy supplies are derived from fossil resources such as coal, oil, and natural gas.^[^ ^1^, ^2^ ^]^ Fossil resources are non‐renewable and will be eventually exhausted. Simultaneously, excessive consumption of fossil resources has caused serious environmental and ecological problems, such as air pollution and climate change.^[^ ^3^ ^]^ In this context, it is extremely imperative to explore green and renewable energy to power our economy. Hydrogen is regarded as an attractive energy carrier for a post‐fossil era because of its merits of high energy density, zero carbon emission, and multiple uses.^[^ ^4^, ^5^, ^6^ ^]^ In particular, its high gravimetric energy of 120 kJ g^−1^ far outstrips most fuels (e.g., 56 kJ g^−1^ for methane, 47 kJ g^−1^ for gasoline, 30 kJ g^−1^ for ethanol, 15 kJ g^−1^ for coal).^[^ ^7^ ^]^ By 2050, H_2_, as a vital energy vector, is predicted to account for 18% of global energy requirements, which can be broadly extended to transportation, industrial, and household appliances.^[^ ^8^, ^9^ ^]^ Meanwhile, hydrogen, as a broad‐spectrum chemical, also exhibits great potential in various applications (e.g., synthetic ammonia through the Haber‐Bosch process, petrochemical manufacturing, and refining steel).^[^ ^10^, ^11^ ^]^ The global market of H_2_ is estimated to reach US$700 billion by 2050.^[^ ^12^ ^]^ Regrettably, more than 95% of hydrogen is produced from thermocatalytic steam reforming of fossil resources (e.g., natural gas, coal, and petroleum) under high temperatures and high pressure with the virtual limitations of huge thermal input, fierce carbon footprint, and concomitant environmental issues (**Figure** **1**a), which is known as gray hydrogen.^[^ ^13^, ^14^ ^]^ Specifically, during the process of gray hydrogen production, ≈830 million tons of carbon dioxide are generated annually, accounting for more than 2% of global carbon dioxide emissions.^[^ ^15^, ^16^ ^]^ It is not sustainable and cannot meet the massive demand for green hydrogen by a carbon‐neutral economy system in the future.^[^ ^17^, ^18^ ^]^ It is thus greatly desired to explore disruptive strategies for green hydrogen production using renewable feedstocks (e.g., biomass, H_2_O) and clean energy inputs (e.g., solar energy, wind energy).^[^ ^19^, ^20^ ^]^

Nowadays, there are three main routes for green hydrogen production from non‐fossil resources: water electrolysis,^[^ ^21^ ^]^ photocatalytic water splitting,^[^ ^22^ ^]^ and biomass photoreforming.^[^ ^23^ ^]^ Electrolysis of water holds a grand promise to generate green hydrogen, where H_2_ is generated at the cathode while O_2_ is released at the anode. Thermodynamically, 1.23 V applied voltage as the energy input is theoretically needed to drive the reaction, but at least 1.8 V in practical application is usually required due to the sluggish kinetics of the oxygen generation reaction, resulting in a large amount of energy consumption and high cost.^[^ ^24^ ^]^ For example, the main cost of water electrolysis originates from electricity. High electricity consumption of 4.5–5.5 kWh m^−3^ is often required for this process in practice, where the electricity usually comes from fossil resources via thermal power, thus suffering from low economic competitiveness with a market share of less than 1%.^[^ ^25^, ^26^ ^]^ Additionally, photocatalytic pure water splitting (2H_2_O + light → 2H_2_ (g) + O_2_ (g)) offers an attractive way to prepare green H_2_ using unlimited and free solar energy.^[^ ^27^ ^]^ From a kinetic perspective, water molecules are extremely stable, and the cleavage O─H chemical bond of H_2_O requires a high energy of 500 kJ mol^−1^. Meanwhile, the reaction is an uphill process with a large thermodynamic barrier (H_2_O → H_2_ + 0.5O_2_, Δ*E* = −1.23 eV) and the sluggish half‐reaction kinetics of water oxidation toward O_2_, which significantly limits the efficiency of hydrogen production. Although considerable efforts have been devoted to this emerging research field, many challenges (e.g., low light harvesting efficiency, severe charges recombination, sluggish reaction kinetics) remain unaddressed.^[^ ^28^, ^29^, ^30^ ^]^ In addition, the facile reverse reaction of water splitting (O_2_ + H_2_ → H_2_O) over the photocatalyst surface, in conjunction with the severe performance degradation of photocatalysts arising from photocorrosion and leaching of active sites, behave as two critical obstacles of photocatalytic hydrogen production from water. What is more, the associated cost and safety issues of H_2_/O_2_ separation during photocatalytic overall water splitting also remain a bottleneck of this dream technology toward commercial applications.

As a sharp contrast, the photoreforming of biomass provides an appealing approach for producing green H_2_ by using solar energy.^[^ ^31^, ^32^ ^]^ In a broad sense, biomass includes all the organics that originate from various plants and animals like lignocellulose from forestry and agricultural residues, sugar from cane, oils, and fats from waste cooking oil and organic wastes, and etc.^[^ ^33^, ^34^ ^]^ Lignocellulosic/plant biomass is naturally produced from water, CO_2,_ and sunlight via photosynthesis with an annual global yield of 170 billion tons.^[^ ^35^, ^36^ ^]^ Overall, biomass is renewable, widely distributed, and carbon‐neutral with rich content of C, H, and O elements, thus being a suitable substitute for fossil resources.^[^ ^37^, ^38^ ^]^ Meanwhile, biomass is also considered as organic solid waste and the main source of environmental pollution. For example, lignin, as one of the three key components of lignocellulose, was the major pollutant of pulping and paper‐making industry with an annual yield of 50–70 million tons. In this context, the valorization of biomass into high‐value derivatives can provide one of the most attractive approaches to process the organic solid waste. Especially, through biological hydrolysis, acid/base hydrolysis, or pyrolysis, biomass can be transformed into a broad range of value‐added derivatives, including alcohols, aldehydes, carboxylic acids, saccharides, and so forth.^[^ ^39^, ^40^, ^41^ ^]^ It is noteworthy that because of the high hydrogen content, biomass and its derivatives are regarded as a proper reservoir for on‐site green hydrogen production.^[^ ^42^, ^43^, ^44^ ^]^ Furthermore, photocatalysis is a fascinating means for the sustainable production of H_2_ from biomass using virtually unlimited solar energy. In photocatalytic process, the redox species, e.g., electrons and holes, or reactive intermediates like radicals are readily generated under mild conditions. These active species can drive the thermodynamically and kinetically unfavorable reactions at room temperature, thus offering a new opportunity for surpassing the limitations of conventional thermocatalysis.

Photocatalytic hydrogen production from biomass has received increasing research interest owing to the following advantages: 1) Photocatalytic pure water splitting is both kinetically and thermodynamically unfavorable since the reaction is an uphill process with large thermodynamic barrier (H_2_O → H_2_ + 0.5O_2_, Δ*E* = −1.23 eV) and the half‐reaction of water oxidation toward O_2_ is a four‐electron reaction with sluggish kinetics.^[^ ^45^, ^46^, ^47^ ^]^ In contrast, the photoreforming of biomass for H_2_ evolution (Taking photocatalytic glucose reforming as an example, C_6_H_12_O_6_ + 6H_2_O → 12H_2_ + 6CO_2_, Δ*E* = −0.001 eV) is much more kinetically favorable and requires much less energy input.^[^ ^48^ ^]^ It is obvious that the biomass reforming reaction is nearly energy‐neutral (Δ*E* = −0.001 eV). Thereby, the reaction can be initiated by utilizing low‐energy photons (i.e., visible and IR light), which account for >90% of the solar energy.^[^ ^49^, ^50^ ^]^ Meanwhile, the hydrogen purity from photocatalytic biomass reforming is often higher than that from water splitting since the photoexcited holes can be consumed by biomass rather than water. Under such a circumstance, there is no oxygen generation simultaneously. As a result, the reverse reaction of H_2_ and O_2_ (H_2_ + O_2_ = H_2_O) can be effectively inhibited in the process of biomass photoreforming.^[^ ^51^, ^52^ ^]^ Meanwhile, the cost and safety issues associated with H_2_/O_2_ separation can be evitable. Furthermore, the quantum yield of photocatalytic biomass reforming (above 70%) is much higher than that (≈2.4%) of photocatalytic water splitting without biomass.^[^ ^53^ ^]^ As discussed above, due to its low thermodynamic barrier, low‐energy photons, e.g., visible and infrared light can be utilized for biomass photoreforming, thus promising to maximally utilize the entire solar spectrum.^[^ ^54^, ^55^ ^]^ 2) Compared with thermocatalysis, photoreforming biomass provides an environment‐friendly pathway for green hydrogen supply. For instance, solar energy is known as a clean, nearly free, and inexhaustible resource. Solar‐driven biomass reforming can be achieved under ambient conditions by photocatalysis. In sharp contrast, thermal catalytic reforming usually suffers from intensive thermal input, harsh operation conditions/cost, and massive CO_2_ emission.^[^ ^56^, ^57^, ^58^ ^]^ The ambient reaction conditions render photocatalytic biomass reforming substantially compelling compared to the thermal catalytic process.^[^ ^59^, ^60^ ^]^ 3) Biomass and its derivatives are an excellent raw feedstock for hydrogen production, because it is not only an abundant, CO_2_‐neutral, and renewable alternative to fossil resources but also an effective H_2_ carrier due to its high hydrogen content.^[^ ^61^ ^]^ Meanwhile, the photoreforming of biomass is also considered as an economically competitive approach by the simultaneous valorization of organic solid waste. Photocatalytic hydrogen production from biomass can be realized through direct and/or indirect pathways. In the direct pathway, raw biomass is directly used as feedstock for photocatalytic hydrogen production. It often suffers from a relatively low hydrogen production rate compared to the indirect pathway, which is primarily associated with the complex and stubborn structure of raw biomass.^[^ ^62^ ^]^ For the indirect method, raw biomass is first converted into biomass derivatives with simplified molecular structure, and subsequently, the derivatives are photoreformed, enabling hydrogen production from biomass with kinetic advantage.

**Figure 2:**
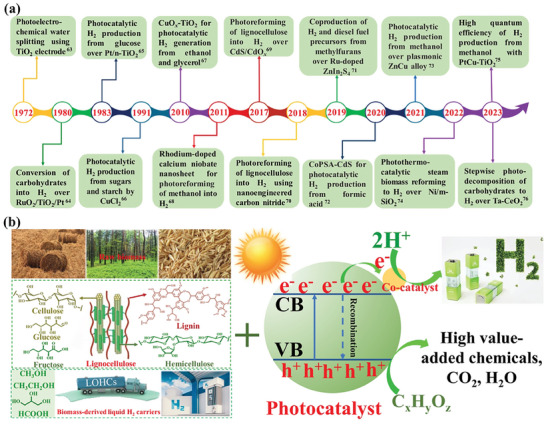
a) Timeline of the mainstream achievements for photocatalytic H_2_ production from biomass. b) Schematic illustration of photocatalytic reforming of biomass into H_2_.

In 1972, photoelectrochemical water splitting was implemented by using Pt‐decorated TiO_2_ as an electrode for the first time,^[^ ^63^ ^]^ which exhibits the enormous potential of semiconductors in the transformation of light energy into chemical energy. In 1980, the photoreforming of biomass for H_2_ production was subsequently reported using TiO_2_‐supported RuO_2_ and Pt as biomass oxidation and hydrogen evolution co‐catalysts, respectively, under ultraviolet illumination.^[^ ^64^ ^]^ Since then, the photoreforming of biomass has attracted increasing research interest and progressed significantly. A growing number of studies on photocatalytic hydrogen production from biomass have been reported. The timeline of the mainstream achievements for photocatalytic H_2_ production from biomass is presented in **Figure** **2**a.^[^ ^63^, ^64^, ^65^, ^66^, ^67^, ^68^, ^69^, ^70^, ^71^, ^72^, ^73^, ^74^, ^75^, ^76^ ^]^ It is noteworthy that the development of highly active, robust, and cost‐effective semiconductor‐based photocatalysts is the key to realize the practical application of photocatalytic hydrogen production from biomass.^[^ ^7^ ^]^ In recent years, substantial progress has been witnessed in the development of semiconductor‐based photocatalysts for this grand topic (Figure 2a). Thus far, a couple of reviews that focused on photocatalytic H_2_ generation from H_2_O and/or biomass have been reported.^[^ ^77^, ^78^, ^79^ ^]^ Nevertheless, a comprehensive summary of the most recent advances in semiconductor‐based photocatalysts for hydrogen generation from solar‐driven reforming of biomass and its derivatives is still rare, especially including the discussion of the emerging photo‐thermal‐coupling strategy, on‐site hydrogen evolution from biomass‐derived liquid organic hydrogen carriers and concurrent generation of value‐added chemicals. Herein, this review commits to providing state‐of‐the‐art progress in semiconductor‐based photocatalysts for H_2_ production from biomass and its derivatives (Figure 1b). Basic principles of biomass photoreforming for H_2_ generation are first introduced for a better understanding of the reaction mechanism. The reported semiconductor materials and cocatalysts with regard to the photoreforming of biomass and its derivatives to H_2_ are then summarized. What is more, various types of feedstocks derived from biomass for photocatalytic H_2_ production are then elaborately discussed with an emphasis on the effect of semiconductor‐based photocatalysts and catalytic systems. The coproduction of value‐added chemicals with H_2_ generation from biomass reforming is discussed at the same time. Of note, the emerging photo‐thermal coupling strategy that shows a grand prospect for maximally utilizing the entire solar energy spectrum is also discussed. Further, the direct utilization of hydrogen from biomass aqueous solution as a green reductant for producing value‐added chemicals via organic reactions is also highlighted. The two aforementioned routes present an economically promising strategy for biomass valorization. In the end, the challenges and perspectives in the field are discussed.

## 2 Basic Principles of Photocatalytic H_2_ Production from Biomass

Generally, biomass photoreforming reaction proceeds through the following three steps,^[^ ^80^ ^]^ as shown in Figure 2b: 1) the photocatalyst is excited by photons with energy equal to or higher than the bandgap of the semiconductor. The charge carriers, i.e., electron (e^−^)–hole (h^+^) pairs are generated, and the photoexcited electrons (e^−^) in the valence band (VB) are then pumped to the conduction band (CB) of the photocatalyst while the photogenerated holes (h^+^) are left in the VB; 2) The photogenerated e^−^ and h^+^ are migrated to the photocatalyst surface, respectively; 3) Protons (H^+^) are subsequently reduced to H_2_ by e^−^ on the reduction sites of the photocatalyst surface while biomass is simultaneously oxidized to chemicals or CO_2_, H_2_O by h^+^ on the oxidation sites of the photocatalyst surface. Of note, the majority of photoexcited charge carriers suffer from severe radiative and non‐radiative recombination, remaining a bottleneck of photocatalytic reforming of biomass toward H_2_.

Compared with photocatalytic water splitting, H_2_ evolution from biomass photoreforming is often kinetically and thermodynamically favorable.^[^ ^81^, ^82^ ^]^ H_2_ evolution from biomass is thermodynamically promising for maximizing the utilization of solar energy with wider wavelength regions (e.g., visible or even IR light) considering the extremely low Gibbs free energy of biomass reforming.^[^ ^83^ ^]^ It should be noted that the isotope labeling experiment proved that in some cases, H_2_ mainly originated from protons in water rather than biomass in the process.^[^ ^84^ ^]^ In contrast, by using some special bio‐derived substrates, such as methanol, ethanol, and furfural alcohol, it is found that the produced H_2_ can mainly come from protons of alcohol substrates.^[^ ^85^ ^]^ Therefore, the proton sources in H_2_ production from photorefining biomass still need further exploration. Biomass oxidation is a very complex process, especially if using raw biomass as the feedstock, because of the complicated molecular structure of natural biomass and the involvement of a multi‐step reaction of different biomass substrates. Of note, biomass can be activated by highly active species e.g., h^+^, ^•^OH, or ^•^O_2_^1−^ and converted into corresponding chemicals or CO_2_, H_2_O.^[^ ^86^, ^87^ ^]^

## 3 Semiconductors for Photocatalytic H_2_ Production from Biomass

**Figure 3:**
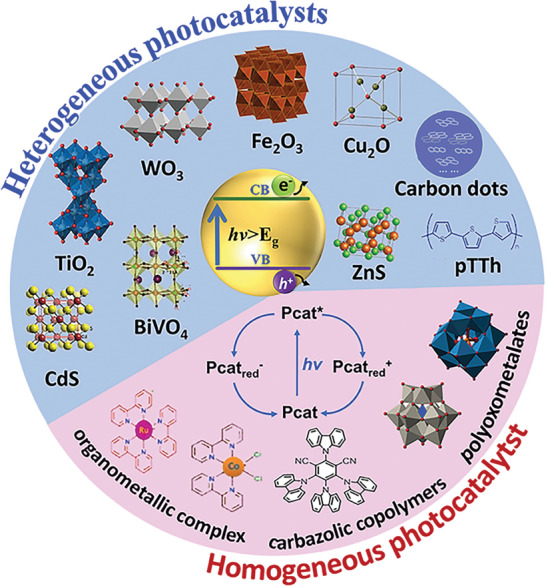
Potential heterogeneous and homogenous photocatalysts for photoreforming of biomass into H_2_. Reproduced with permission.^[^ ^88^ ^]^ Copyright 2022, Cell Press.

**Figure 4:**
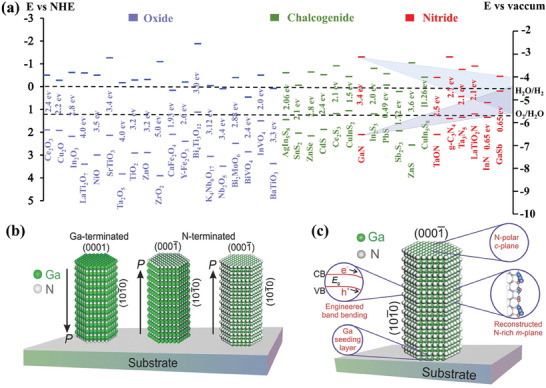
a) Band edge positions and bandgaps with respect to the vacuum level and the normal hydrogen electrode (NHE) for oxides, chalcogenides, nitrides semiconductors and tunable Ga(In, Sb)N band structures in comparison with various semiconductors. Reproduced with permission.^[^ ^112^ ^]^ Copyright 2016, Wiley‐VCH. b) Models for single crystal wurtzite GaN nanowires. c) Schematic of the key atomic and electronic structure of the epitaxial GaN. Reproduced with permission.^[^ ^151^ ^]^ Copyright 2016, Wiley‐VCH.

Photocatalyst is the protagonist of biomass reforming toward hydrogen driven by sunlight and the semiconductor is the basic component of photocatalyst. To date, a variety of photocatalysts have been developed for the grand topic (**Figure** **3**).^[^ ^88^, ^89^, ^90^ ^]^ In general, photocatalysts are classified into homogeneous photocatalysts, such as molecular organometallic complexes^[^ ^91^ ^]^ and some water‐soluble polyoxometalates,^[^ ^92^ ^]^ and heterogeneous photocatalysts, such as TiO_2_, BiVO_4_, CdS, and g‐C_3_N_4_ semiconductor‐based photocatalysts.^[^ ^93^ ^]^ Compared with homogeneous photocatalysts, heterogeneous photocatalysts, especially semiconductor‐based photocatalysts, demonstrate remarkable advantages of easy fabrication/recycling, low cost, environmental friendliness, and ease of scaling up.^[^ ^94^ ^]^ Thus, the review mainly focuses on the recent progress of semiconductor‐based photocatalysts for H_2_ generation from biomass. Semiconductor‐based photocatalysts are basically composed of light‐harvesting semiconductors and co‐catalysts.^[^ ^95^ ^]^ Semiconductor, as a core component of photocatalyst, is capable of harvesting photons and generating charge carriers with redox potentials under light irradiation.^[^ ^96^ ^]^ In principle, when the bandgap of the semiconductor is equal to the theoretical potential of water splitting (1.23 eV), photocatalytic H_2_ evolution reaction may occur. In practice, the bandgap energy of a semiconductor is usually required to be greater than 1.8 eV due to the thermodynamic and kinetic barriers of the reaction. However, there is an undesired compromise between broad‐range light absorption and adequate redox potentials. Thus far, a variety of semiconductors have been investigated for photoreforming of biomass into H_2_, including but not limited to oxides (e.g., TiO_2_,^[^ ^97^ ^]^ ZnO,^[^ ^98^ ^]^ WO_3_,^[^ ^99^ ^]^ Bi_2_O_3_,^[^ ^100^ ^]^ Fe_2_O_3_,^[^ ^101^ ^]^ BiVO_4_^[^ ^102^ ^]^), chalcogenides (e.g., CdS,^[^ ^103^ ^]^ ZnS,^[^ ^104^ ^]^ MoS_2_^[^ ^105^ ^]^), nitrides (e.g., g‐C_3_N_4_,^[^ ^106^ ^]^ GaN,^[^ ^107^ ^]^ Ta_3_N_5_,^[^ ^108^ ^]^ BN^[^ ^109^ ^]^), and porous crystalline polymers (e.g., covalent organic frameworks,^[^ ^110^ ^]^ metal–organic frameworks^[^ ^111^ ^]^). The band structure of most of the aforementioned semiconductors is shown in **Figure** **4**a and Table S1 (Supporting Information).^[^ ^112^ ^]^ Meanwhile, the advantages and disadvantages of the representative semiconductors are listed in **Table** **1**. Despite significant advances, there has been no virtual success in meeting the optical and electronic demand for photocatalysts. A suitable trade‐off between light absorption and redox potentials is highly desirable for the achievement of high‐efficiency biomass photoreforming toward H_2_.^[^ ^77^ ^]^

**Table 1:** Advantages and disadvantages of the representative semiconductors.

Semiconductors		Advantages	Disadvantages
Types	Representatives		
Metal oxides	TiO_2_	Good stability, non‐toxicity, cheapness	Inefficient light adsorption, high recombination rate of the photogenerated carriers
	BiVO_4_	Low cost, low toxicity, good photostability, visible light response	Short lifetime of charge carriers
Chalcogenides	CdS	Low cost, visible light response	Serious photocorrosion, rapid recombination of photogenerated electron‐hole pairs, heavy metal toxicity
Nitrides	g‐C_3_N_4_	Metal‐free, low cost, nontoxicity, visible light response, facile in preparation, high chemical stability	Low surface area, serious recombination of photogenerated carriers, high synthesis temperature (>500 °C)
Porous crystalline polymers	MOF, COF	Structural designability, uniform porous structure, high surface area	Sensitivity to acid, base, and humid atmospheres

### 3.1 Metal Oxides‐Based Semiconductors

Metal oxides are one of the most studied semiconductors for photocatalysis due to their appealing features, such as excellent stability, cheapness, and earth abundance. Regrettably, most metal oxides are limited by their large bandgap and can only absorb a small proportion of the solar spectrum, leading to unsatisfactory photocatalytic efficiency. Meanwhile, their photocatalytic activities are also inhibited by the short lifetime of charge carriers. Among a broad range of metal oxides‐based semiconductors, TiO_2_ is the most extensively studied semiconductor due to the fascinating features mentioned above^[^ ^113^, ^114^ ^]^ Nevertheless, thus far, there has been limited breakthrough in hydrogen production with high efficiency by utilizing TiO_2_‐based photocatalysts. It is basically associated with the large bandgap (3.2 eV) and high recombination rate of the photogenerated carriers.^[^ ^115^ ^]^ In detail, the wide bandgap of TiO_2_ makes it can only respond to ultraviolet light with a wavelength of shorter than 387 nm, accounting for approximately 3–5% of the total solar energy.^[^ ^116^ ^]^ Constructing a heterojunction with different semiconductors is an effective strategy to overcome the above issues by broadening the light‐responsive range and/or enhancing e^−^/h^+^ pairs separation.^[^ ^117^ ^]^ For example, the n‐p heterojunction of TiO_2_‐NiO with ultrafine core–shell structure was prepared by anchoring NiO nanoclusters onto ultrafine TiO_2_ nanoparticles for photoreforming of lignin into H_2_.^[^ ^118^ ^]^ The n‐p heterojunction of TiO_2_‐NiO greatly inhibited the recombination of photogenerated electrons and holes, thus promoting the activity. The H_2_ evolution rate of TiO_2_–NiO reached 0.45 mmol g^−1^ h^−1^ in 1 m NaOH solution under UV–vis light irradiation, which was ≈3.56 and 11.1 times higher than that of pure TiO_2_ and NiO, respectively. Zinc oxide (ZnO), as a low‐cost, abundant, and non‐toxic metal oxide, has also been extensively investigated as a promising semiconductor for photocatalysis.^[^ ^119^ ^]^ However, it is also capable of only absorbing ultraviolet light owing to the large bandgap of ≈3.3 eV. Moreover, the photocatalytic efficiency is also obstructed by the short lifetime of charge carriers as well.^[^ ^120^ ^]^ Tungsten oxides exhibit multiple oxidation states (e.g., W^6+^, W^5+^, and W^4+^), excellent stability, and a relatively narrow bandgap of ≈2.6 eV, which can be responsive to absorb 12% of the solar spectrum. These merits make Tungsten oxides a compelling semiconductor candidate to fabricate an appropriate photocatalyst.^[^ ^121^, ^122^, ^123^ ^]^ Fe_2_O_3_, as an n‐type semiconductor with a 2.2 eV bandgap, exhibits a strong visible light absorption capacity, thus promising to achieve high solar‐to‐fuel energy conversion efficiency.^[^ ^124^ ^]^

### 3.2 Metal Sulfide Semiconductors

**Figure 5:**
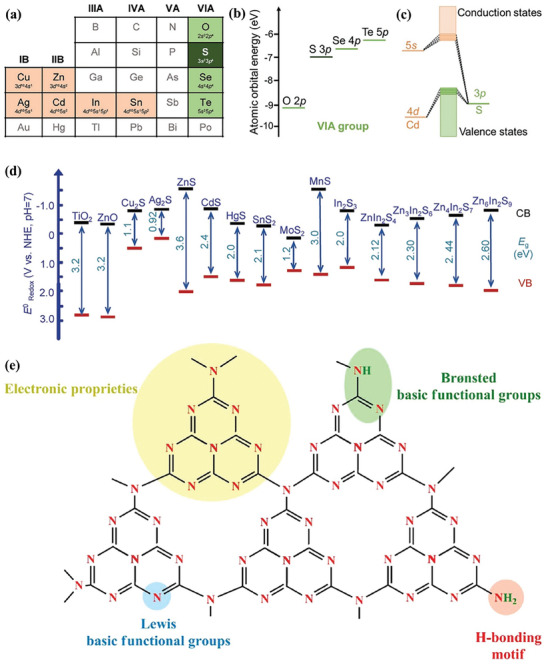
a) Part of the periodic table with highlighted the metals and chalcogenides for constructing of the metal sulfides, b) atomic orbital energies of O, S, Se, and Te, c) illustrative electronic structure of CdS, and d) band positions of some typical oxides and sulfides. Reproduced with permission.^[^ ^125^ ^]^ Copyright 2021, Wiley‐VCH. e) Multifunctional characteristics of C_3_N_4_ material. Reproduced with permission.^[^ ^136^ ^]^ Copyright 2020, the American Chemical Society.

Metal sulfides have emerged as an outstanding class of semiconductors for photocatalysis. Commonly, metal sulfides are composed of metals with d10 configuration, including Zn, Cd, In, and Cu, and chalcogenide p, such as S, Se, and Te (**Figure** **5**a). For metal sulfides, their CB edges are mainly contributed from s or p orbitals of metal, whereas VB edges are primarily derived from 3p orbitals of chalcogenide (Figure 5c). The atomic orbital energies of the chalcogenide p are close to each other but clearly different from that of O 2p as shown in Figure 5b.^[^ ^125^ ^]^ Owing to the higher VB position of S 3p orbital compared with that of the O 2p orbital, metal sulfides exhibit stronger reduction ability and narrower bandgap compared to metal oxides (Figure 5d). Thus, metal sulfides, such as ZnS, CdS, and ZnIn_2_S_4_, possess a suitable bandgap, showing an appreciable compromise between broad‐range light absorption and sufficient redox potentials. For instance, CdS with a bandgap of 2.4 eV can absorb visible light with wavelengths up to 516 nm while the band edge positions still well straddle the redox potentials of water splitting and biomass reforming.^[^ ^126^ ^]^ In spite of proper optical and electronic properties for photocatalysis, metal sulfides usually suffer from fierce photocorrosion problems because they are vulnerable to self‐oxidation by photogenerated holes (CdS + h^+^ → Cd^2+^ + S).^[^ ^127^ ^]^ The photocorrosion issue remains a bottleneck to the long‐term stability of CdS‐based photocatalysts. Meanwhile, their performance is also limited by the rapid recombination of photogenerated electrons and holes.^[^ ^128^ ^]^ In recent studies, it was found that both doping and heterostructure engineering were viable for addressing the aforementioned issues.^[^ ^129^ ^]^ For instance, P‐doped Zn_0.5_Cd_0.5_S‐P with rich S vacancies was fabricated through a combination of doping P element and constructing Z‐scheme heterostructure.^[^ ^130^ ^]^ After interstitial P doping, the Fermi level of Zn_0.5_Cd_0.5_S‐P was increased by 0.25 eV compared with that of Zn_0.5_Cd_0.5_S, and the impurity level of S vacancies in Zn_0.5_Cd_0.5_S‐P is approximate to the Fermi level of Zn_0.5_Cd_0.5_S. The resultant S vacancies play as outstanding electron trap centers and thus facilitated extending the lifetime of photogenerated electrons and inhibiting the recombination of photogenerated electrons and holes. This endeavor affords P‐doped Zn_0.5_Cd_0.5_S‐P measurable photocatalytic activity in hydrogen evolution from 5‐hydroxymethylfurfural (5‐HMF) aqueous solution, which is obviously superior to that of pristine ZnS‐P and CdS‐P. The optimal Zn_0.5_Cd_0.5_S‐P showed an impressive H_2_ evolution rate of 786 µmol g^−1^ h^−1^ from 5‐HMF aqueous solution under visible light irradiation and its activity has a negligible decrease after 8 h. Although Cd‐containing sulfides have achieved notable achievements in photorefining of biomass into H_2_, their heavy metal toxicity cannot be ignored.

### 3.3 Nitride Semiconductors

**Figure 6:**
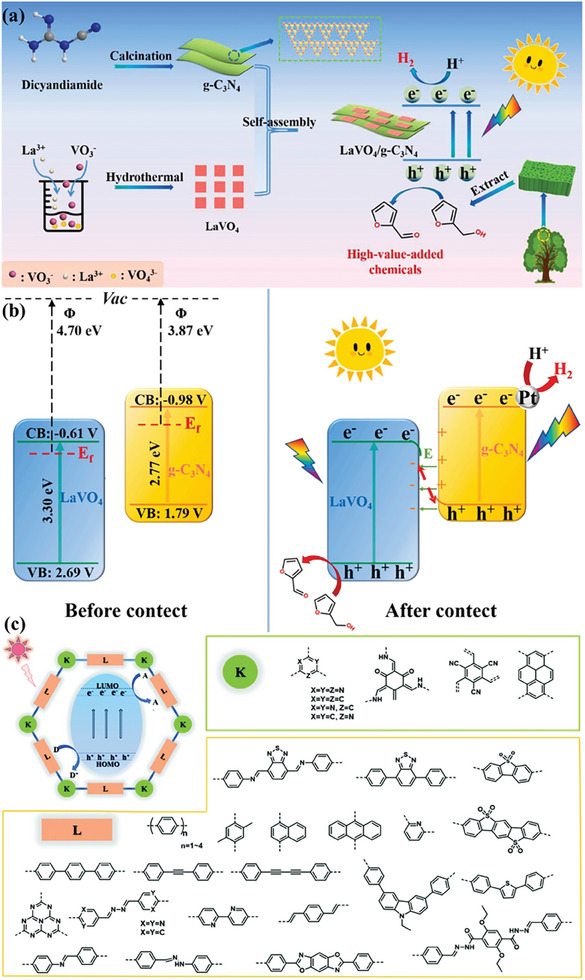
a) Schematic illustration of preparing 2D/2D LaVO_4_/g‐C_3_N_4_ photocatalyst for H_2_ evolution from furfuryl alcohol, and b) photocatalytic mechanism of H_2_ production over 2D/2D LaVO_4_/g‐C_3_N_4_ heterostructure. Reproduced with permission.^[^ ^143^ ^]^ Copyright 2022, Elsevier Inc. c) Structures of COF semiconductors (K denotes knots; L denotes linkers). Reproduced with permission.^[^ ^165^ ^]^ Copyright 2020, The Royal Society of Chemistry.

Since the successful synthesis of carbon nitride (C_3_N_4_) was reported in 1993,^[^ ^131^ ^]^ C_3_N_4_ has quickly become a rising‐star material, which was extensively investigated for multiple fields such as hydrogen generation,^[^ ^132^, ^133^ ^]^ biomass valorization,^[^ ^134^, ^135^ ^]^ CO_2_ transformation^[^ ^136^, ^137^ ^]^ and pollutant degradation.^[^ ^138^ ^]^ Particularly, g‐C_3_N_4_, as a typical non‐metallic semiconductor consisting of N, C, and H elements, is composed of a tri‐s‐triazine network interlinked by planar amino groups (Figure 5e). Basically, g‐C_3_N_4_ possesses electron‐rich characteristics, basicity, and hydrogen‐bonding groups due to the presence of abundant H and N atoms compared with carbon‐based materials (Figure 5e).^[^ ^136^ ^]^ Meanwhile, owing to the appealing attributes of low cost, nontoxicity, remarkable acid–alkali chemical resistance, and outstanding thermal stability, g‐C_3_N_4_ is taken into account as an interesting nominee in photocatalytic H_2_ production.^[^ ^139^ ^]^ More interestingly, g‐C_3_N_4_ is able to interact with lignocellulose and/or its derivatives by π‐π interaction and hydrogen bonding, which is conducive to activating the complex and stubborn lignocellulose molecules.^[^ ^140^ ^]^ Together with the suitable bandgap (2.70 eV), g‐C_3_N_4_ has appeared as a promising visible‐light responsive metal‐free semiconductor platform for photocatalytic reforming of lignocellulose.^[^ ^141^ ^]^ Nevertheless, the photocatalytic activity of raw g‐C_3_N_4_ was substantially inhibited by the fierce recombination of photogenerated electrons and holes, limited light absorption range, and low specific surface area. To address this critical issue, various strategies, such as the engineering of monolayer structure, surface dyadic heterostructure, and chemical modification, have been conducted to enhance and introduce new properties for accelerating photocatalytic H_2_ production. Recently, there have been endeavors to improve the performance of C_3_N_4_ by heterojunction engineering and functional group modification.^[^ ^142^ ^]^ As a typical example, the LaVO_4_/g‐C_3_N_4_ heterostructure was constructed by anchoring square LaVO_4_ nanoflakes on the surface of thin‐layer g‐C_3_N_4_ nanosheets using the thermal treatment method for photocatalytic H_2_ evolution from biomass‐derived furfuryl alcohol (**Figure** **6**a).^[^ ^143^ ^]^ The constructed LaVO_4_/g‐C_3_N_4_ showed an H_2_ production rate of 0.287 mmol g^−1^ h^−1^, which is three times that of pristine g‐C_3_N_4_ due to its abundant 2D/2D heterointerfaces that enables the effective separation of photogenerated electrons and holes (Figure 6b). Moreover, the introduction of functional groups in C_3_N_4_ can adjust the energy band structure. Typically, when the terminal amino of C_3_N_4_ was substituted with ethyl alcohol by an in‐situ C‐N coupling method, a new discrete energy level in the bandgap was generated, resulting in a significant enhancement in the visible‐light absorption capacity.^[^ ^144^ ^]^ Meanwhile, ethyl alcohol serves as an electron donor, thus enabling the formation of an internal electric field to promote the separation of photogenerated electrons and holes and extend the lifetime of photoexcited charges. As a result, the H_2_ yield of the modified C_3_N_4_ reaches 136.9 µmol from d‐xylose, which is ≈5.93‐fold that of pristine C_3_N_4_ (23.1 µmol) under the same experimental conditions. In spite of a series of great progress, the utilization of C_3_N_4_ for solar‐powered production of H_2_ from biomass is not suitable yet for practical applications.^[^ ^145^ ^]^

Ga(X)N (X = In, Mg, Ge, Si, Sb, Al, etc.), as an emerging semiconductor, have demonstrated significant advances in solar‐driven water splitting to hydrogen,^[^ ^146^ ^]^ CO_2_ reduction toward fuels,^[^ ^147^, ^148^ ^]^ N_2_ fixation,^[^ ^149^ ^]^ as well as an organic reaction.^[^ ^150^ ^]^ First of all, compared to the reported semiconductors above, Ga(X)N exhibited a widely tunable bandgap by incorporating various dopants with different concentrations. Meanwhile, the charge behavior can be mediated by quantum structure engineering (quantum dot, and quantum nanolayer). Moreover, the built‐in electric field can be achieved by spatial gradient doping with specific dopants through molecular beam epitaxy (MBE) and metal–organic chemical vapor deposition (MOCVD). Overall, state‐of‐the‐art material growth technologies endow Ga(X)N with highly tunable structure, morphology, and optoelectronic properties. Furthermore, the surface properties can be also engineered to mediate the adsorption and activation behavior of the complex biomass molecule with abundant functional groups (─OH, C─H, COOH, C─C, C─O, OCH_3_, and aromatic rings; Figure 4).^[^ ^151^, ^152^, ^153^, ^154^ ^]^ As discussed above, Ga(X)N nanowires integrated with silicon substrate thus appear as next‐generation light absorption platforms for solar‐driven reforming of biomass toward H_2_. The progress of Ga(X)N/Si nanoarchitecture on solar‐driven water splitting^[^ ^155^, ^156^ ^]^ and CO_2_ reduction.^[^ ^157^, ^158^ ^]^ Most recently, sunlight‐driven hydrogen generation from biomass and its derivatives have also been attempted by employing Ga(X)N/Si nanoarchitecture.^[^ ^159^, ^160^ ^]^ Besides, Zn_3_N_2_ is an n‐type semiconductor with about 3.4 eV bandgap. It can be synthesized by nitridation of zinc powder at 600 °C for 2 h in an ammonia atmosphere.^[^ ^161^ ^]^ Hexagonal boron nitride (h‐BN) is a type of a 2D material with a similar structure to graphite.^[^ ^162^ ^]^ The utilization of h‐BN as a semiconductor for photocatalysis has also become a research hotspot owing to its many advantages, such as remarkable thermal and electrical conductivity, high stability, and large surface area.^[^ ^163^ ^]^ Nevertheless, the h‐BN can only absorb ultraviolet light with a wavelength of <310 nm, which restricts its photocatalytic activity. Overall, nitride semiconductors present a promising family of building blocks to develop disruptive photocatalysts for sunlight‐driven biomass reforming toward H_2_, especially in conjunction with other suitable light absorbers.

### 3.4 Porous Crystalline Polymers

Covalent organic frameworks (COFs) are a type of crystalline porous network materials constructed from organic monomers by covalent bonds link, displaying exceptional structural designability and uniform porous structure with ultra‐high surface areas.^[^ ^164^ ^]^ Meanwhile, the π‐conjugated character of both the knot and the linker is of considerable benefit for promoting light harvesting and charge carriers migration (Figure 6c).^[^ ^165^ ^]^ Such fascinating properties render COFs with enormous prospects in photocatalysis.^[^ ^166^ ^]^ In this direction, the triazine‐based frameworks CTF‐1‐100W (C_8_N_2_H_4_) with ordered porous structure, composed of alternating triazine units and phenyl groups, were prepared by microwave‐assisted polymerization. The CTF‐1‐100W showed a bandgap at ≈2.48 eV, which is 0.49 eV smaller than that of g‐C_3_N_4_, resulting in a wider visible light absorption up to 500 nm. What is more, it exhibited an ≈0.3 eV deeper valence band position than that of g‐C_3_N_4_, thus displaying a stronger oxidization capability than g‐C_3_N_4_. Notably, the ordered planar structure can efficiently alleviate the photogenerated e^−^/h^+^ recombination. Thus, as‐prepared CTF‐1‐100W sample exhibited an H_2_ production rate of 5.50 mmol g^−1^ h^−1^ from methanol and triethanolamine aqueous solution, which is 50‐fold higher than that of g‐C_3_N_4_.^[^ ^167^ ^]^ Meanwhile, given the advantages described earlier, COFs, especially 2D COFs, have also been popularized as excellent platforms for the growth of active nanoparticles to enhance their photocatalytic activity. For example, a highly stable 2D COF (TpPa‐2) was developed by a solvothermal reaction of 1,3,5‐triformylphloroglucinol and 3,6‐dimethyl‐1,4‐diaminophenyl as a matrix for growth of CdS nanoparticles to synthesize a series of hybrid materials.^[^ ^168^ ^]^ The as‐synthesized hybrid materials were investigated for photocatalytic hydrogen production from lactic acid solution. Interestingly, upon introduction of merely 1 wt% of the COF content, a significant increase in H_2_ production rate with 1320 µmol g^−1^ h^−1^ was found, which was approximately tenfold and 47‐fold higher than of the bulk CdS (128 µmol g^−1^ h^−1^) and the COF (28 µmol g^−1^ h^−1^), respectively. When the content of the COF increased to 10%, named CdS‐COF (90:10) hybrid exhibited an optimal H_2_ production activity with a rate of 3678 µmol g^−1^ h^−1^, which was much higher than the bulk CdS and the COF. The π‐conjugated backbone, abundant 2D heterointerface, and high surface area of the CdS‐COF hybrid are beneficial for increasing the lifetime of photogenerated charge carriers and stabilizing CdS, thereby resulting in a superior photocatalytic activity.

**Figure 7:**
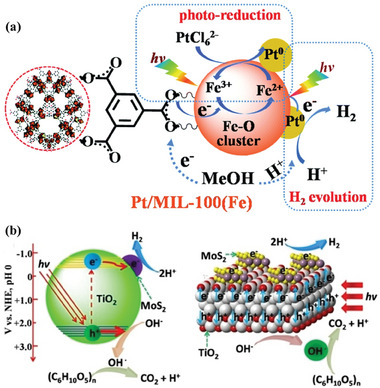
a) Proposed mechanism for photo‐reduction of deposited Pt nanoparticles and photocatalytic H_2_ evolution over Pt/MIL‐100(Fe). Reproduced with permission.^[^ ^171^ ^]^ Copyright 2016, The Royal Society of Chemistry. b) Photogenerated electron transfer and proposed mechanism for photorefining of lignocellulose into H_2_ over 2% MoS_2_/TiO_2_ photocatalyst. Reproduced with permission.^[^ ^205^ ^]^ Copyright 2021, Wiley‐VCH.

Metal–organic frameworks (MOFs) are another family of crystalline porous materials with uniform pores structures formed by connecting metal ions or metal clusters and organic ligands via coordination bonds.^[^ ^169^ ^]^ As noted, both metal centers and organic ligands can be designed. As a result, the structural, optoelectronic, and catalytic properties of MOF can be flexibly regulated by coupling organic linkers with metal centers.^[^ ^170^ ^]^ Meanwhile, the porous structure of MOFs with high surface area offers ideal platforms for loading catalytic centers. In addition, both organic ligands and uncoordinated organic ligands in MOFs can immobilize active sites through post‐modification. The geometric and electronic properties of active sites can be further engineered to facilitate H_2_ evolution from biomass and its derivatives. Typically, MIL‐100(Fe), as a water‐stable MOF material, is composed of trimers of FeO_6_ octahedra sharing a common vertex μ_3_‐O and 1,3,5‐benzenetricarboxylic acid as an organic ligands.^[^ ^171^ ^]^ MIL‐100(Fe) is a potential semiconductor for photocatalysis because its existing iron‐oxo (Fe‐O) clusters can be excited by visible light irradiation. Thus, the Pt/MIL‐100(Fe) photocatalyst was synthesized by in‐situ photodeposition of Pt nanoparticles on the surface of MIL‐100(Fe) for photocatalytic H_2_ evolution from methanol solution (**Figure** **7**a). In the process of photocatalytic H_2_ generation, the Fe‐O clusters are first stimulated to generate an excited charge separation state by transferring an electron from the O^2−^ to Fe^3+^ to form Fe^2+^ under visible light irradiation. The Pt nanoparticles as electron traps can capture the photo‐generated electrons from the Fe‐O clusters via the heterojunction between Pt and Fe–O clusters in MIL‐100(Fe). The H^+^ from methanol is reduced to H_2_ on Pt nanoparticles, and methanol serves as an electron donor to close the reaction cycle by consuming the holes. An optimum H_2_ production rate of 109 µmol g^−1^ h^−1^ was achieved in methanol solution over 0.8 wt% Pt/MIL‐100(Fe). As new stars in semiconductor‐based photocatalysis, the issues of porous crystalline polymers such as complex synthesis process, high cost, limited stability, and electronic conductivity need to be well addressed. In view of its porous structure, good chemical stability, and availability of functional modification, NH_2_‐MIL‐68(In) has served as an ideal platform for the assembly of semiconductors and catalytically active sites. Consequently, the Cosal‐NH_2_‐MIL‐68@In_2_S_3_ photocatalyst was fabricated by immobilization of molecular Co(II)‐salicylaldimine catalyst on MOF@In_2_S_3_ using post‐synthetic modification. It is noted that a novel MOF@In_2_S_3_ heterojunction was generated by in situ sulfurization process.^[^ ^172^ ^]^ The sulfurized heterojunction is beneficial for boosting the separation and transfer of photogenerated charge carriers, displaying a remarkable H_2_ production rate of 18746 µmol g_cat_^−1^ h^−1^ with >99.9% selectivity and an apparent quantum efficiency of 3.8% at 420 nm.

The structure of semiconductors plays a vital role in determining the hydrogen generation activity by affecting the physicochemical and optical properties. The structure parameters (i.e., bandgap structure, morphology, size, and crystal phase) exerting more influence were discussed. The bandgap structure of the semiconductor theoretically should be larger than the potential barrier of H_2_ evolution reaction from pure water splitting (1.23 eV). However, the bandgap energy in practical applications usually requires to be above 1.8 eV due to the thermodynamic loss and overpotential.^[^ ^55^ ^]^ Except for the bandgap energy, the conduction band and valence band potentials of semiconductors are also crucial for hydrogen evolution reaction. The conduction band potential should be more negative than the reduction potential of H_2_ (−0. 41 V), while the valence band potential requires to be more positive than the oxidation potential of the biomass. Morphology and size of the semiconductor also are two critical parameters in the photocatalytic hydrogen production from biomass, because they affect the physicochemical properties of photocatalyst, such as specific surface area and number of surface active sites. Normally, the specific surface area and number of surface active sites increase as the particle size reduces, resulting in an improvement in hydrogen production efficiency. This can be explained by improving contact efficiency between the photocatalysts and substrates. The crystal phase of the semiconductor has a significant impact on the pathway of photoreforming of biomass, thereby affecting corresponding hydrogen production activity. For example, TiO_2_ co‐doped with fluorine and Pt (Pt–F–TiO_2_) was developed by Iervolino et al. for photocatalytic hydrogen production from glucose.^[^ ^173^ ^]^ It is found that anatase is a more selective cleavage of the C–C bond in the glucose molecules compared to rutile, which promotes the production of hydrogen.

Overall, semiconductor, as a vital component of photocatalyst, plays an extremely important role in the entire process of photocatalysis: i) solar light harvesting; ii) photo‐generated e^−^/h^+^ pairs separation; iii) coordination with active sites for surface chemicals reactions. To date, various semiconductors, such as metal oxides, chalcogenides, nitrides, and porous crystalline polymers, have been explored for photocatalytic hydrogen production from raw biomass and its derivatives. Although enormous research efforts have been devoted to developing efficient semiconductors for photocatalytic H_2_ production, some challenges still need to be addressed for industrial applications, including weak absorption of visible and infrared light, easy recombination of photogenerated electrons and holes, and sluggish surface reaction kinetic. To address these challenges, many strategies have been proposed, such as deposition cocatalysts (e.g., Ag, Pt, Ru, Rh, Pd, Au, Co, Ni, and Cu), constructing heterojunction, doping elements, and surface dye sensitization. When a co‐catalyst is deposited on the semiconductor surface, the photo‐generated electrons can facilely transfer from the semiconductor to the cocatalyst until the energy levels of both are equal, because the work function of the cocatalyst is usually higher than that of the semiconductor. This can effectively suppress the recombination of photogenerated electrons and holes, thereby promoting photocatalytic efficiency. A rational heterojunction formed by combining two different band‐matched semiconductors shows a grand promise for improving the optical and electronic properties of the photocatalyst via immobilization or doping methods. In particular, the light absorption capability of a wide bandgap semiconductor can be extended by combining with a semiconductor with a narrow bandgap. Meanwhile, the built‐in electric field of heterojunction formed between the two different semiconductors is capable of efficiently suppressing the e^−^/h^+^ recombination, which is critical for increasing the probability of charges participating in surface reactions. According to the type of doped elements, element doping can be classified into metal doping (e.g., Co, Cu, Fe, Mo, Cr), non‐metal doping (e.g., N, S, P, F, Cl, B), and metal/non‐metal co‐doping. By element doping, both light absorption capacity and redox potentials of semiconductors can be broadly tailored, thus promising to make a desirable trade‐off for photocatalytic reforming of biomass toward H_2_. It can also form charge traps to capture photogenerated electrons and holes, thereby inhibiting the recombination of photogenerated charge carriers. Surface dye sensitization refers to the modification of semiconductors with photosensitizers to extend the light response range of semiconductors. Photosensitizers usually have strong visible light absorption ability, including some inorganic or organic chromophores. Surface dye photosensitization has also been recognized as one of the most popular methods for improving the efficiency of light utilization. It is highly encouraged to optimize photosensitization for improving semiconductor‐based photocatalysis of H_2_ generation from biomass.

## 4 Cocatalysts for Photocatalytic H_2_ Generation from Biomass

The combination of co‐catalysts, such as metals,^[^ ^174^ ^]^ metal oxides,^[^ ^175^ ^]^ metal phosphides,^[^ ^176^ ^]^ and sulfides,^[^ ^177^ ^]^ with light‐harvesting semiconductor has been validated to be an efficient strategy to improve the photocatalytic activity and stability. Co‐catalysts, as an indispensable component of photocatalysts, contributed to the improved performance owing to the following reseasons.^[^ ^178^ ^]^ First of all, if some kind of special metals like Cu, Au, and Ag are employed as co‐catalysts, they are capable of extending light absorption by the surface plasmonic resonance effect.^[^ ^179^ ^]^ Meanwhile, loading co‐catalysts on the semiconductor surface can result in the formation of Schottky heterojunction, which can promote the separation and migration of photogenerated carriers. Most importantly, co‐catalysts can provide active sites for the activation of specific chemical bonds, thus lowering the energy barriers of both H_2_ generation and biomass reforming.^[^ ^180^ ^]^ In addition, the stability of the semiconductor can be also enhanced by timely consuming the photo‐generated holes, thus avoiding the undesired decomposition of the semiconductor.^[^ ^181^ ^]^ Among a broad range of materials studied, noble metals (e.g., Pd, Pt, Ir, Au, Ag, Ru, Rh) are validated to be state‐of‐the‐art cocatalysts for H_2_ evolution. It is associated with the remarkable attributes that facilitate reducing the activation energy of the reaction and/or trapping the photogenerated electrons. It is worth noting that some kinds of special metals (e.g., Au, Ag, Cu, Pd) exhibit localized surface plasmon resonance (LSPR, collective oscillation phenomenon of the metallic conduction band electrons with the electromagnetic field of the incident light) effect.^[^ ^182^, ^183^ ^]^ The LSPR effect can broaden the light absorption range.^[^ ^184^ ^]^ Importantly, the LSPR effect can also generate high local temperature of the photocatalyst and energetic hot carriers, thus benefiting the solar‐to‐hydrogen energy efficiency.^[^ ^185^, ^186^ ^]^ Meanwhile, noble metal nanoparticles can effectively inhibit the recombination of photogenerated electrons and holes by transferring photogenerated electrons from the semiconductor to metallic active sites owing to their small Fermi level and large work function. It is found that the activity of noble metals‐decorated TiO_2_ for photocatalytic H_2_ evolution from methanol is much higher than that of the single TiO_2_.^[^ ^187^ ^]^ The catalytic activity of P25 titania decorated with various metals for photo‐reforming of methanol was compared, and the H_2_ production rate declined in the order of Pd > Pt > Ir > Au > Ru ≈ Rh > Ni.^[^ ^188^ ^]^ Notably, M (M = Pd, Pt, Au, Ni) was also coupled with TiO_2_ for testing the hydrogen evolution capability from the key component of lignocellulose—cellulose.^[^ ^189^ ^]^ Compared with pristine TiO_2_, all the decorated photocatalysts showed greatly enhanced H_2_ activity, whereas the H_2_ production rate followed the descending trend of Pd/TiO_2_ > Pt/TiO_2_ > Ni/TiO_2_ ≈ Au/TiO_2_. Herein, the first step in the photoreforming of cellulose into H_2_ is the (photo)hydrolysis of cellulose into glucose. Then the obtained glucose is converted into carbon dioxide (CO_2_) and H_2_ via C–C bond cleavage on the metal cocatalyst.

Considering the high cost and rareness of noble metals, the exploration of earth‐abundant metals e.g., Cu, Ni, Co, and Fe as practical alternatives for photocatalytic H_2_ production has drawn growing interest.^[^ ^190^, ^191^, ^192^ ^]^ Noble metal‐free co‐catalysts mainly consist of metal and/or metal oxides, metal sulfides, and metal phosphides. Immobilization of different metal oxides on the TiO_2_ surface was explored for photocatalytic H_2_ production from glycerol aqueous solution. The as‐prepared photocatalysts demonstrated a reduced activity order of CuO/TiO_2_ > NiO/TiO_2_ > CoO/TiO_2_.^[^ ^193^ ^]^ In this case, Cu species were effective for the water gas shift reaction (CO + H_2_O → CO_2_ + H_2_) and NiO facilitated the C–C bond scission in the reaction.^[^ ^194^ ^]^ In addition to metal and/or metal oxides, metal sulfides and phosphides (e.g., MoS_2_, Ni_x_S_y_, CoP, Ni_2_P) also exhibit great potential as alternatives to noble metals for H_2_ production from biomass photoreforming. Over recent years, MoS_2_ has been widely recognized as a promising alternative to noble metals, benefitting from its weak binding energy to *H, a graphite‐like layered structure with rich active sites, and outstanding stability arising from the covalent characteristic.^[^ ^195^ ^]^ For instance, photocatalytic H_2_ production from alcohol aqueous solution over CdS was evidently improved by loading MoS_2_ as a cocatalyst.^[^ ^196^ ^]^ For CdS semiconductors, the photocatalytic activity of MoS_2_ cocatalyst was even higher than that of precious metal Pt. This is attributed to that MoS_2_ is easier to form the desirable intimate junction with CdS than Pt because the majority of the (002) planes of MoS_2_ are parallel to the CdS surface. The intimate junction formed between MoS_2_ and CdS promotes electron transfer, which is mainly responsible for the enhanced photocatalytic activity of the MoS_2_/CdS catalyst. Similarly, Ni*_x_* S*_y_* was explored to decorate ZnS nanorods for photocatalytic H_2_ generation from benzyl alcohol, in the concurrent formation of benzaldehyde. The production of both H_2_ and benzaldehyde were enhanced, and the H_2_ production rate over ZnS/Ni*_x_* S*_y_* (3.65 mmol g^−1^ h^−1^) was over five times higher than that over ZnS (0.73 mmol g^−1^ h^−1^).^[^ ^197^ ^]^ It was found that Ni*_x_* S*_y_* promoted dehydrogenation of the OH group of benzyl alcohol due to its strong proton adhesion. Meanwhile, Ni*_x_* S*_y_* can effectively capture photogenerated electrons to reduce H^+^ to H_2_ and swiftly desorb the produced benzaldehyde, thereby resulting in the achievement of a high hydrogen production rate and a high benzaldehyde selectivity. Metal phosphides are also a typical family of non‐noble metal co‐catalysts for photocatalytic H_2_ generation. What is more, metal phosphides, such as Fe_x_P, CoP_3_, and Ni_2_P, exhibit a broad wavelength absorption ranging from ultraviolet to visible light, even near‐infrared light, owing to their dark appearance and narrow bandgap.^[^ ^198^ ^]^ For instance, Zhao et al. found that Fe_x_P was broadly responsive to the photons in the wavelength range from 350 to 800 nm.^[^ ^199^ ^]^ What is more, as reported, the decoration of CoP_3_ would broaden the light absorption range of Mn_0.2_Cd_0.8_S, enabling a red shift of the absorption edge owing to its efficient absorption from visible light to near‐infrared light.^[^ ^200^ ^]^ Further, benefiting from its rich active sites, and enhanced e^−^/h^+^ separation, CoP was coupled with Zn_2_In_2_S_5_ for enhancing H_2_ evolution and diols formation via photocatalytic reforming of methanol and ethanol.^[^ ^201^ ^]^ In this study, the selective activation of α‐C─H of the alcohols occurred over Zn_2_In_2_S_5_ without affecting the O─H group. The generation of radical intermediates (i.e., ^•^CH_2_OH radical from methanol, and ^•^CH(OH)CH_3_ radical from ethanol) was consequently realized with the assistance of photoexcited holes (CH_3_OH + h^+^ → ^•^CH_2_OH + H^+^; CH_3_CH_2_OH + h^+^ → ^•^CH(OH)CH_3_ + H^+^), followed by C─C coupling to diols. Meanwhile, CoP cocatalyst can effectively capture the photo‐generated electrons to reduce H^+^ for H_2_ evolution. Thus, the ethylene glycol formation rate of CoP/Zn_2_In_2_S_5_ is fivefold higher than that of bare Zn_2_In_2_S_5_. In spite of environmental friendliness, cheapness, and promising catalytic properties, the existing noble metal‐free co‐catalysts are still far away from practical applications because of their limited activity and inferior stability. Of note, the rational design of oxidation cocatalysts for biomass valorization to value‐added chemicals rather than overoxidation toward CO_2_ is critical for making photocatalytic reforming of biomass economically viable. Therefore, tremendous efforts are still required to improve the activity, selectivity, and durability of cost‐effective cocatalysts by cooperating with a suited semiconductor platform.

## 5 Raw Biomass Photoreforming for H_2_ Production

**Figure 8:**
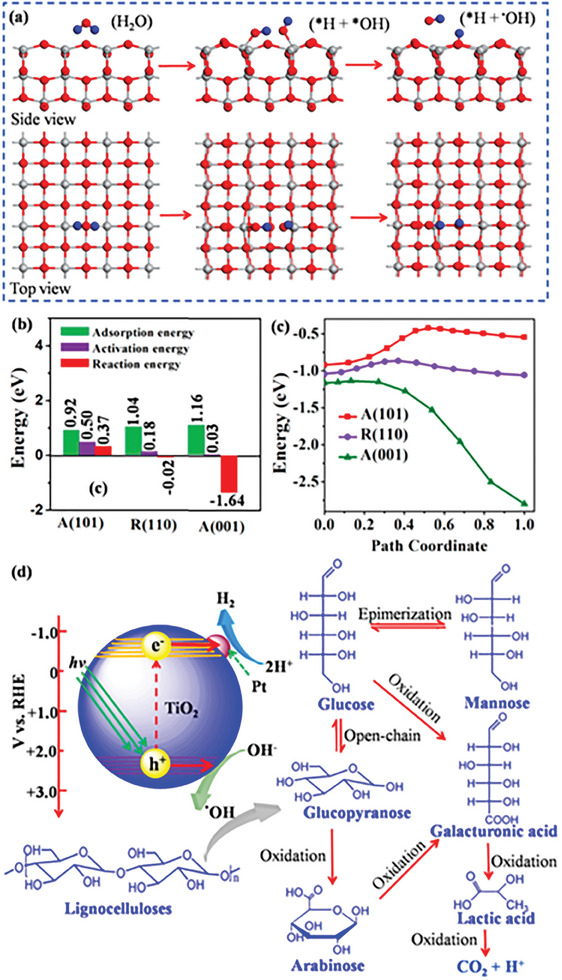
a) Schematic diagram of the ^•^OH generation on (001) facets of anatase TiO_2_. b) Comparison of the absorption, activation, and reaction energy for the generation of ^•^OH over (101) and (001) facets of anatase TiO_2_ and (110) facets of rutile TiO_2_. c) Reaction path for the generation of ^•^OH over (101) and (001) facets of anatase TiO_2_ and (110) facets of rutile TiO_2_. d) Energy diagram of charge transfer and proposed reaction mechanism for photoreforming of lignocelluloses into H_2_. Reproduced with permission.^[^ ^206^ ^]^ Copyright 2022, American Chemical Society.

Raw biomass is usually regarded as organic waste and an environmental pollutant. Among them, lignocellulose is the richest component of biomass, which accounts for >50% of dry biomass weight.^[^ ^202^ ^]^ Thus far, burning has remained the main approach for their disposal/utilization, suffering from resource waste and environmental pollution. From a sustainable point of view, photocatalytic reforming of raw biomass/lignocellulose for H_2_ generation is highly beneficial for reducing the cost of H_2_ production and alleviating environmental pollution. Unfortunately, the complexity, recalcitrance, and insolubility of raw biomass including but not limited to lignocellulose pose tremendous challenges for hydrogen production with high efficiency, especially driven by solar energy under ambient conditions.^[^ ^203^ ^]^ Numerous endeavors have been conducted to address the aforementioned issues (**Table** **2**). For example, Pt‐decorated TiO_2_ was employed for directly photoreforming rick husk in neutral water under natural sunlight.^[^ ^204^ ^]^ Because of the inert structure of the native lignocellulose, the photocatalyst illustrated a limited H_2_ production rate of ≈95 µmol g_cat_^−1^ h^−1^. The yield of H_2_ generated from rick husk was about half of that derived from cellulose, which is consistent with the about 54% cellulose content in rice husk. It is indicative that H_2_ primarily originates from cellulose in rice husks. It was found that during the process, the cellulose in the rice husk was first converted into soluble molecules (e.g., glucose and 5‐hydroxymethylfurfural), and the soluble molecules were subsequently oxidized into H_2_. Meanwhile, to improve the contact efficiency between the photocatalyst and biomass, the 2D MoS_2_ nanosheet was anchored onto the surface of the 2D TiO_2_ nanosheet with a specific surface area of 24.1 m^2^ g^−1^ using the hydrothermal method.^[^ ^205^ ^]^ The optimized 2% MoS_2_/TiO_2_ hybrid showed an H_2_ hydrogen production rate of 21.4 µmol g_cat_^−1^ h^−1^ from wood chip aqueous solution at pH 7. It was well recognized that the hydroxyl radical (^•^OH) generated from water oxidation by photogenerated holes is a highly active species for biomass decomposition (Figure 7b). Improving the ^•^OH yield during photocatalysis is considered a feasible strategy to facilitate the photocatalytic biomass‐to‐H_2_ conversion. Cheng et al. found that ^•^OH radicals were facilely generated on the exposed (001) facets of anatase TiO_2_ nanosheets, which was proved by density functional theory (DFT) calculations (**Figure** **8**a–c).^[^ ^206^ ^]^ During the photoreforming of lignocellulose into H_2_, the formation of ^•^OH radicals with strong oxidization ability promoted the depolymerization of lignocellulose, resulting in an enhancement in photocatalytic H_2_ production (Figure 8d). Meanwhile, the nanosheet structure with a thickness of 2.1 nm of TiO_2_ not only accelerates the accessibility with poplar wood chips but also promotes the separation of the photogenerated charges. These features afforded a hydrogen production rate of 26 µmol h^−1^ g^−1^ in 250 mL of 4 g L^−1^ poplar wood chip aqueous solution over an optimal 1.0% Pt/TiO_2_ photocatalyst, which was ≈2.1 times higher than that of the frequently reported anatase TiO_2_ nanosheets.

**Table 2:** Photoreforming of raw biomass for H_2_ generation.

Substrate	Catalyst	Light source	Condition	Rate [µmol g^−1 ^h^−1^]	Reference
wooden branch	Co/CdS/CdOx	100 mW cm^−2^	10 m KOH, 25 °C	5310	[69]
rice straw	Ni/m‐SiO_2_	500 W Xe lamp, 361.0 mW cm^−2^	H_2_O	1488100	[74]
wheat straw				728400	
corn stalk				1036500	
rice husk	0.5% Pt/TiO_2_	sunlight	H_2_O	95	[204]
wood chip	2% MoS_2_/TiO_2_	300 W Xe lamp	H_2_O	21.4	[205]
poplar wood chip	1.0% Pt/TiO_2_	300 W Xe lamp	H_2_O	26	[206]
grass	Pt/CdS/SiC	300 W Xe lamp (λ > 420 nm)	10 m NaOH, 343 K	259.0	[209]
wood				131.7	
paper				60.7	
grass	Cu,In‐doped ZnS	300 W Xe lamp	10 m NaOH, 70 °C	31.7	[210]
wood				60.2	
paper				73.9	
toilet paper	1% Pt g‐C_3_N_4_	300 W Xe (λ > 420 nm), 100 mW cm^−2^	pH 10	2167	[227]

**Figure 9:**
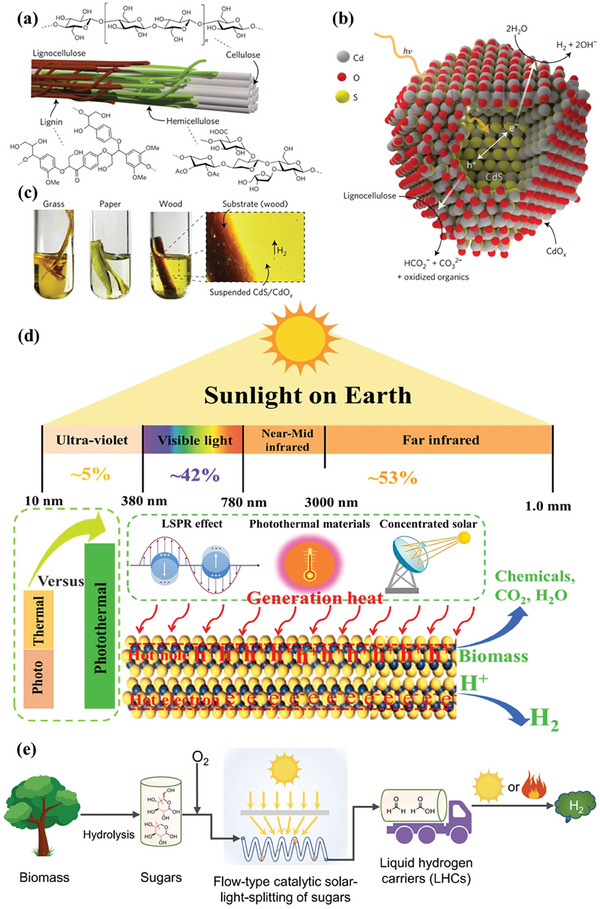
a) Lignocellulose comprised of cellulose, hemicellulose, and lignin, b) Photoreforming of lignocellulose for H_2_ production over CdS/CdO*_x_*, and c) Various raw biomass is photoreformed into H_2_ production over CdS/CdO*_x_* in the alkaline solution. Reproduced with permission.^[^ ^69^ ^]^ Copyright 2017, Springer Nature. d) Schematic illustration of photothermal catalytic biomass for hydrogen production. e) Schematic description of a stepwise photoreforming of raw biomass for H_2_ production. Reproduced with permission.^[^ ^76^ ^]^ Copyright 2023, Cell Press.

As mentioned above, the insolubility of raw biomass in common solvents remains a key challenge for efficient H_2_ production because of the recalcitrance since it limits the effective access of raw biomass to active sites.^[^ ^207^ ^]^ Acid/base pretreatment is an efficient approach to improve H_2_ production by conversion of raw biomass into soluble organic molecules, as well as by in situ modification of the photocatalyst. For example, when CdS quantum dots were tentatively utilized for photoreforming of unprocessed lignocellulose with a particle size of ˂ 0.25 cm to H_2_ in KOH aqueous solution under visible light illumination at 100 mW cm^−2^ (**Figure** **9**a–c),^[^ ^69^ ^]^ the KOH aqueous solution played an important role, not only in promoting the hydrolysis of insoluble lignocellulose into the soluble molecules, but also in facilitating the in situ generation of Cd(OH)_2_/CdO on the surface of CdS quantum dots. The in situ formed Cd(OH)_2_/CdO can serve as a cocatalyst and effectively suppress the CdS photocorrosion. More importantly, the Cd(OH)_2_/CdO promoted the oxidation of lignocellulose to CO_3_^2−^ by weakening the C─C bonds by forming analogous Cd–O–R bonds with the lignocellulosic substrates. As a result, a decent H_2_ production rate of 5.31 mmol g_cat_^−1^ h^−1^ was achieved by feeding a tree branch. Moreover, the production rate can be varied from 1 to 9 mmol g_cat_^−1^ h^−1^ using different waste papers, which is associated with the difference in the molecular structure of the feedstocks.

Compared to pure photocatalysis, photo‐thermal‐catalysis not only effectively improves the efficiency of photocatalysis, but also enhances solar energy utilization efficiency. In the process of photothermal catalysis, low‐energy visible and even infrared light, accounting for a large proportion of the whole solar energy, can be effectively converted into heat through the LSPR effect of the metallic nanoparticles, excited photothermal materials, and concentrated solar energy (Figure 9d). Compared with thermal catalysis, photo‐thermal‐catalysis can well address the most critical issues of thermal catalysis, such as high energy consumption and harsh reaction conditions. The stability can also be improved because the generation of some undesirable byproducts such as coke that lead to catalyst deactivation is avoided under relatively mild conditions. As a result, the catalytic performance of photo‐thermal‐catalysis is often superior to pure photocatalysis and thermal catalysis although the reaction mechanism has remained largely unknown. Photo‐thermal‐catalysis has been proven as a powerful and promising approach for effectively sunlight‐driven biomass photoreforming by the synergy of photo‐excited charge carriers and photoinduced heat, which enabled a substantial reduction in the activation energy barrier.^[^ ^208^ ^]^ To maximally utilize the different regions of the solar spectrum to produce H_2_ from raw biomass, photo‐thermal multifunctional architectures were boldly explored. For example, a semiconductor hybrid of Pt/CdS/SiC was designed for this grand topic.^[^ ^209^ ^]^ In this hybrid, visible light with a wavelength shorter than 580 nm was used to produce excited charge carriers while the remaining part was absorbed by SiC to generate heat through the non‐radiative recombination of photoexcited charge carriers. Consequently, the reaction system can be heated up to 343 K under a 300 W Xe lamp irradiation. The localized high temperature is favorable for the swelling and hydrolysis of lignocellulose in an alkaline solution. Together with photogenerated electrons and holes, the grass, wood, and paper were photoreformed into H_2_ at rates of 259.0, 131.7, and 60.7 µmol g_cat_^−1^ h^−1^ of H_2_ in 10 M NaOH solution, respectively. What is more, photo‐thermo‐catalytic steam biomass reforming toward H_2_ was achieved over Ni nanoparticles‐loaded mesoporous silica (Ni/m‐SiO_2_) using focused illumination of the entire solar spectrum.^[^ ^74^ ^]^ In this case, the focused light illumination does not only provide heat but also activates the substrate. Owing to the surface plasmonic absorption of loaded nickel nanoparticles via 3d electron excitation, Ni/m‐SiO_2_ exhibits strong absorption across the entire solar spectrum, resulting in high surface temperature under focused UV–vis–infrared (UV–vis–IR) light illumination. Meanwhile, photooxidation of C* species is substantially accelerated by chemisorbed O* on Ni nanoparticles upon illumination, which is the rate‐determining step of steam biomass reforming. Hence, the H_2_ production rates reached 1488.1 mmol g_cat_^−1^ h^−1^ for rice straw, 728.4 mmol g_cat_^−1^ h^−1^ for wheat straw, and 1036.5 mmol g_cat_^−1^ h^−1^ for corn stalk, respectively, under UV–vis–IR illumination at 361.0 mW cm^−2^. The varied H_2_ production rates may be attributed to different components and structures of the feedstocks tested. The catalytic activities were mainly attributed to the full spectrum strong absorption of loaded nickel nanoparticles and photothermal synergistic catalysis. Isotope labeling experiments validated the participation of water in photocatalytic biomass reforming, where water significantly promoted the generation of H_2_, and decreased the yields of char and tar. Moreover, it was reported that Cu–In‐doped ZnS was photothermally active for biomass reforming toward H_2_ in 10 m NaOH aqueous solution at 70 °C.^[^ ^210^ ^]^ The study showed that the activity of the Cu–In‐doped ZnS photocatalyst (ZnS‐1198) was 125 times higher than that of undoped ZnS. This improvement was ascribed to that doping Cu and In in ZnS facilitated suppressing the recombination of photogenerated electrons and holes. Notably, it was found that the photocatalyst crystallinity also played a vital role in the activity. In particular, the Cu–In‐doped ZnS photocatalyst with high crystallinity synthesized via a MOF was ≈8.9 times higher than that of the photocatalyst with low crystallinity prepared by the solvothermal method because high crystallinity implied few recombination centers. This discovery suggests that engineering the crystallinity of photocatalysts presents an efficient strategy for mediating photocatalytic biomass reforming toward H_2_. The naturally robust and complex molecular structure remains a fundamental challenge for direct photoreforming of raw biomass toward hydrogen, especially in neutral water under ambient conditions. From the viewpoint of chemistry, the multi‐step strategy is superior to direct photoreforming for hydrogen production from raw biomass. For instance, pinewood was first hydrolyzed by 1 v/v% HCl into soluble sugars (glucose and xylose) at 160 °C for 3 h. Then, the obtained hydrolysate was decolorized and detoxified by treating it with activated charcoal to alleviate the detrimental influence of its color and toxin on the subsequent process. Finally, the treated hydrolysate was photoreformed using the 1% Pt/TiO_2_ photocatalyst. The solar‐to‐hydrogen (STH) efficiency of this reported process reached 1.05%. The H_2_ yield from 8 consecutive photocatalytic cycles was 19.9 mL g_cat_^−1^.^[^ ^211^ ^]^ In addition, Ren and co‐workers proposed a “C–C bond first” stepwise strategy of photo‐assisted splitting of biomass toward H_2_.^[^ ^76^ ^]^ First of all, as shown in Figure 9e, poplar and wheat straw can be readily converted to sugars via hydrolysis by feeding H_2_SO_4_. More importantly, the C–C bond of the obtained sugars was fully cleaved to produce C_1_ liquid organic hydrogen carriers (LOHCs), i.e., HCOH and HCOOH over a synergetic Ta‐doped CeO_2_ that utilize the photoenergy and thermal energy, which is highly favorable for H_2_ storage and transportation. The H_2_ yield from glucose through stepwise photoreforming was much higher than that through direct photoreforming of glucose, validating the superiority of the stepwise strategy in H_2_ generation from biomass. In addition to acid pretreatment, thermo‐alkaline hydrolysis pretreatment was also effective to facilitate H_2_ generation from the photoreforming of lignocellulose. In Zhao's attempt, lignocellulose was first hydrolyzed into soluble fragments in 2 mol L^−1^ NaOH solution at 200 °C for 12 h. The obtained hydrolysate exhibited the superior ability to generate hydrogen compared to the two types of common sacrificial agents (i.e., triethylamine and triethanolamine) by employing a commercial SrTiO_3_ photocatalyst.^[^ ^212^ ^]^ The selective production of tartaric acid during thermo‐alkaline hydrolysis of lignocellulose makes a predominant contribution toward the superior performance in this process.

Concentrated solar energy, as a zero‐emission high‐temperature heat source, can provide extreme heating condition, such as high temperatures above 1000 °C and a fast heating rate above 100 °C S^−1^,^[^ ^213^ ^]^ offering favorable conditions for pyrolysis of biomass, especially raw biomass. Compared with traditional pyrolysis, concentrated solar exhibits unique advantages of fast heating rate, unilateral heating of the pellets, and energy saving. Takeda et al. demonstrated that solar pyrolysis of biomass was the least‐carbon intensive pathway among all current hydrogen production methods.^[^ ^214^ ^]^ Carbon dioxide emissions from solar biomass pyrolysis was 1.04 kg CO_2_‐eq kg^−1^ H_2_, which was less than half of the conventional biomass gasification process (2.67 kg CO_2_‐eq kg^−1^ H_2_). Solar pyrolysis of wood was carried out by Zeng et al. and mixed gas (CO, CO_2_, CH_4_, H_2_) was obtained with low H_2_ selectivity.^[^ ^215^ ^]^ Although solar pyrolysis of biomass for hydrogen production is theoretically feasible, the relevant reports are very limited, which may be due to the complex pyrolysis process and poor hydrogen selectivity. Therefore, precise solar pyrolysis of biomass for hydrogen production remains a challenge.

## 6 Photoreforming of Lignocellulosic Components for H_2_ Production

**Figure 10:**
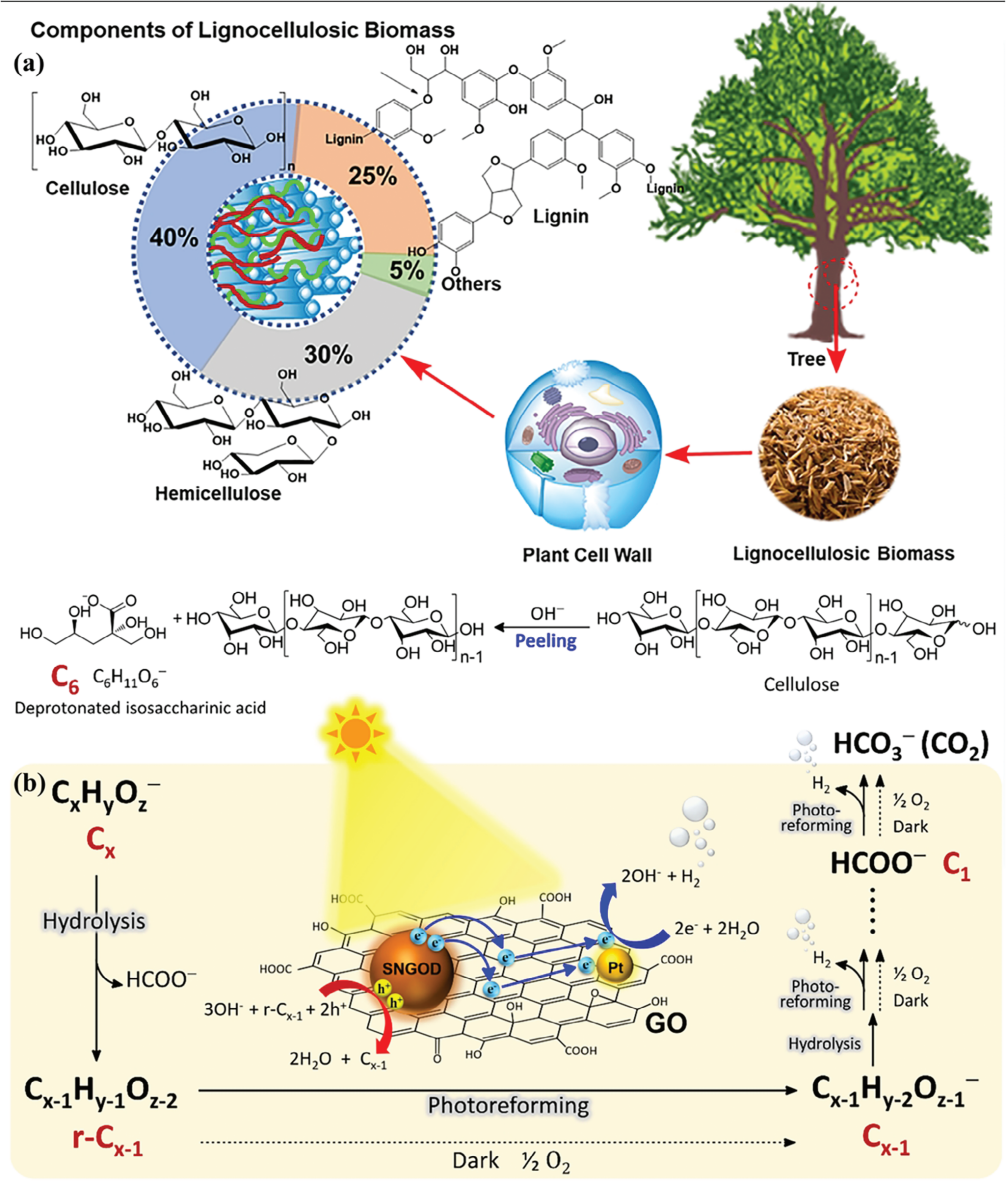
a) Schematic illustration of lignocellulosic components. Reproduced with permission.^[^ ^218^ ^]^ Copyright 2023, American Chemical Society. b) Mechanism of photoreforming of cellulose to H_2_ in NaOH solution. Reproduced with permission.^[^ ^228^ ^]^ Copyright 2021, American Chemical Society.

Again, lignocellulose is the most abundant biomass in nature and accounts for more than 50% of biomass by weight. In practice, lignocellulose can be easily extracted from raw biomass through chemical treatment or enzymolysis.^[^ ^216^, ^217^ ^]^ It is primarily composed of three main components i.e., cellulose, hemicellulose, and lignin (**Figure** **10**a).^[^ ^218^ ^]^ Cellulose, as the most abundant natural polymer accounting for 40–50% of lignocellulose, is a homopolymer composed of linear glucose units linked by β‐1‐4 glycosidic bonds. It is insoluble and stable due to its intra‐ and inter‐molecular hydrogen bonds.^[^ ^219^ ^]^ Hemicellulose, which accounts for 20–30% of lignocellulosic biomass, is a branched polymer containing pentose and hexose. It is well known as a crosslinking agent for cellulose and lignin.^[^ ^220^ ^]^ Lignin is an exclusive class of natural phenolic polymers primarily composed of interlinking phenylpropane units via C–C and C–O bonds and accounts for 18–28% of lignocellulosic biomass.^[^ ^221^ ^]^ Of note, it is also the main waste from the pulp and paper‐making industry with an annual global output of 50–70 million tones and estimated to reach 225 million tonnes per year by 2030.^[^ ^222^ ^]^ Through hydrolysis or enzymolysis, lignocellulose can be converted to a number of derivatives, including cellulose, hemicellulose, lignin, and soluble molecules (e.g., monosaccharide, liquid H_2_ carriers), which largely depends on the processing technology. Compared to the native lignocellulose, the depolymerized derivatives are beneficial for green H_2_ generation powered by sunlight owing to the relatively pure composition, simple structure, and high activity of the obtained bio‐derived compounds. Consequently, the utilization of the derivatives of lignocellulose as the feedstock for photocatalytic H_2_ production is of scientific and practical significance and has aroused great interest (**Table** **3**).

**Table 3:** Representative examples of photocatalytic H_2_ production from lignocellulose derivatives.

Substrate	Catalyst	Conditions	Light source	H_2_ evolution rate	AQY% (λnm)	Reference
cellulose	2% 2D‐2D TiO_2_/MoS_2_	H_2_O	300 W Xe lamp	201 µmol h^−1^ g^−1^	1.45 (380)	[205]
cellulose	1.0% Pt/TiO_2_	H_2_O	300 W Xe lamp	275 µmol h^−1^ g^−1^	1.89 (380)	[206]
cellulose	Bi_2_MoO_6_	10 M NaOH solution, 80 °C	300 W Xe lamp	53.66 µmol h^−1^ g^−1^		[223]
cellulose	0.16 wt% Pt/m‐TiO_2_	H_2_O, 40 °C	UV‐A lamp (365 nm, 16W)	132.6 µmol h^−1^ g^−1^	–	[224]
ball‐milling pretreated cellulose	0.16 wt% Pt/TiO_2_	H_2_O, 40 °C	UV‐A lamp (365 nm, 16W)	110.8 mmol h^−1^ g^−1^	–	[225]
cellulose	Pt/TiO_2_	0.6 M H_2_SO_4_ solution	250 W, iron‐doped halide lamp	1.32 mmol h^−1^ g^−1^	–	[229]
cellulose	Pt/TiO_2_	25 wt.% LiBr, 0.1M H_2_SO_4_, 25 °C	100 mW cm^−2^	180 µmol h^−1^ g^−1^	–	[230]
cellulose	P25‐S‐Ni	H_2_O, 80 °C	500 W Xe lamp, 400 mWcm^−2^	3.02 mmol h^−1^ g^−1^	–	[231]
cellulose	Pt/CdS/SiC	10 M NaOH solution, 343 k	300 W Xe lamp (λ > 420 nm)	361.5 µmol h^−1^ g^−1^	19.6% (380 nm)	[209]
lignin				71.5 µmol h^−1^ g^−1^		
lignin and lactic acid	0.2‐NiS/CdS	H_2_O	300 W Xe lamp (λ > 400 nm)	1512.4 µmol h^−1^ g^−1^	AQE:44.9%	[234]
kraft lignin	SiF/Ni‐NGQD	H_2_O	450 W Xe Lamp (100 mW cm^−2^)	14.2 mmol h^−1^ g^−1^	AQE: 37% (400 nm)	[235]
cellulose	1.0% wt Pt/monolayer g‐C_3_N_4_	pH = 12 aqueous solution	300 W Xe‐lamp (λ > 420 nm)	73 µmol h^−1^ g^−1^	–	[227]
hemicellulose				60 µmol h^−1^ g^−1^	–	
lignin				21µmol h^−1^ g^−1^	–	
glucose	TiO_2_ P25	H_2_O	256 W/m^2^	6.6 mmol h^−1^ g^−1^	–	[241]
glucose	P25‐CQDs‐H‐2	H_2_O	300 W Xenon lamp	2.43 mmol h^−1^ g^−1^	–	[244]
glucose	Pt NPs/PRCN	H_2_O, 80 °C	300 W Xenon lamp, 2.0 W m^−2^	72 µmol h^−1^ g^−1^	–	[245]
glucose	Zn_0.3_Cd_0.7_S	NaOH DMSO and H_2_O	300 W Xe lamp	13.64 mmol h^−1^ g^−1^	8.2 (500)	[248]
glucose	Pt_0.21_‐C_3_N_4_	10 M NaOH aqueous	40 W blue LED (*λ* = 427 nm	3.39 mmol h^−1^ g^−1^	–	[249]
xylose	Pt/HCN‐NEA	5 M NaOH aqueous	300 W Xe‐lamp	4092 µmol h^−1^ g^−1^	7.87 (420)	[250]
xylose	RuP_2_/Ti_4_P_6_O_23_@TiO_2_‐7	5 M KOH solution	visible light irradiation	16.3 mmol h^−1^ g^−1^	–	[252]

AQE: apparent quantum efficiency

### 6.1 Cellulose and Hemicellulose

Photocatalytic reforming of cellulose and/or hemicellulose presents a clean strategy for H_2_ production by directly feeding the inedible biomass ((C_6_H_10_O_5_)n + 7nH_2_O ═ 12nH_2_ + 6nCO_2_,). It was discovered that the incorporation of cellulose into the reaction system could enormously enhance photocatalytic H_2_ evolution from H_2_O. The H_2_ rate was ten times higher than that of the reaction system in water without cellulose.^[^ ^204^ ^]^ Despite great significance, the robust molecular structure of cellulose and hemicellulose, resulting from various inert chemical bonds and abundant hydrogen bonds network, remains a substantial obstacle to the achievement of highly efficient photocatalytic H_2_ production. The insoluble nature of most solvents makes the process more challenging. For instance, although A‐Bi_2_MoO_6_ can theoretically drive the hydrogen production from cellulose, it was nearly not active under 300 W Xe lamp irradiation at 25 °C for 4 h.^[^ ^223^ ^]^ Thus far, tremendous efforts have been devoted to abstract hydrogen from cellulose and hemicellulose (Table 3). Among the reported studies, TiO_2_ is the most widely used semiconductor material. The Pt/TiO_2_ photocatalyst prepared by wet impregnation was explored for photocatalytic reforming of cellulose toward H_2_.^[^ ^224^ ^]^ As we know, the Pt species promote the reaction by serving as the photoexcited electron traps for efficient separation of photogenerated electrons and holes and by acting as active sites for lowering the energy barrier of cellulose reforming toward H_2_. However, excessive Pt loading was detrimental to light harvesting of TiO_2_, thus causing a reduced photocatalytic activity. Under an optimized condition, H_2_ production rate reached 132.6 µmol h^−1^ g^−1^ over 0.16 wt% Pt/TiO_2_ in a 1.0 g L^−1^ cellulose concentration irradiated by a UV‐A lamp at 40 °C. To further promote cellulose‐to‐H_2_ conversion, the (001)‐facets‐exposed anatase TiO_2_ nanosheets were developed to provide rich active sites for photocatalytic H_2_ generation reaction and for promoting photogenerated charge migrate to the photocatalyst surface. Remarkably, the 94.5% exposed (001) facets (TiO_2_‐1) facilely produce center dot OH radicals, which is highly active for breaking the inert chemical bond network of cellulose.^[^ ^206^ ^]^ As a result, the H_2_ production rate over 1.0% Pt/TiO_2_ was enhanced to 275 µmol h^−1^ g^−1^ with an apparent quantum yield of 1.89% at 380 nm. Porous structure engineering was also extensively conducted to improve the activity by facilitating the accessibility of bio‐derived substrates to the surface of photocatalysts. As a successful attempt, 2D few‐layer MoS_2_ nanosheet was loaded onto the surface of 2D anatase TiO_2_ nanosheet to assemble a unique 2D‐2D porous structure.^[^ ^205^ ^]^ The smart 2D–2D porous structure provides an efficient charge transfer channel due to the intimate 2D nanojunction. It therefore inhibits the recombination of photogenerated electrons and holes and improves the catalytic activities of the catalysts. The optimal 2% 2D–2D TiO_2_/MoS_2_ showed an improved H_2_ generation rate of 201 µmol h^−1^ g^−1^ from neutral cellulose aqueous solution and an apparent quantum yield (AQY) of 1.45% was recorded at 380 nm.

It is well known that physical pretreatment is often beneficial for promoting the interaction between the substrate and photocatalyst, thus benefitting the reaction. In particular, ball‐milling is a viable and popular pretreatment for cellulose. As reported by Fan and coworkers, ball‐milling was utilized to significantly reduce the particle size, crystallinity index, and degree of polymerization of microcrystalline cellulose.^[^ ^225^ ^]^ It thus favors the contact between cellulose and catalytic sites of the photocatalyst. The H_2_ production rate was consequently increased from 66.6 to 110.8 mmol h^−1^ g _Pt_^−1^ without varying other experimental conditions, indicating that ball‐milling is a simple and viable strategy for enhancing cellulose‐to‐H_2_ conversion by reducing the particle size of cellulose.

Facile access of the reactants to active sites remains a huge challenge for heterogeneous photocatalysis of insoluble cellulose. Unlike ball‐milling, the conversion of cellulose into soluble small molecules under a strong acidic or alkaline environment via hydrolysis can further enhance the accessibility of the reactants to the active sites because of the high dispersion and solubility of the derivative molecules in water.^[^ ^226^ ^]^ In this context, strong bases or acids were usually added to the reaction system for promoting the solubility of cellulose, thus favoring hydrogen production from cellulose. The effect of the KOH concentration in the reaction system on the photocatalytic activity over the CdS/CdO*_x_* photocatalyst was studied.^[^ ^69^ ^]^ As expected, the activity was boosted by increasing the KOH concentration due to the improved solubility of cellulose in a higher‐concentrated KOH solution. Isotope‐tracking studies showed that H_2_ primarily originated from both water and cellulose. With the use of CdS/CdO*_x_* decorated with Co(BF_4_)_2_ as the photocatalyst, comparable hydrogen production rates were achieved for cellulose (2.57 mmol_ _g_cat_^−1^ h^−1^) and hemicellulose (2.32 mmol g_cat_^−1^ h^−1^) in 10 m KOH solution. The findings above offer a clear indicator that the transformation of cellulose into soluble small molecules is an effective strategy for improving hydrogen generation. However, it may suffer from the virtual limits of high cost, complex process, and environmental issues.

As noted, since high temperatures can promote the hydrolysis of cellulose in alkaline solution, photothermal catalysis was also attempted to accelerate H_2_ generation from α‐cellulose over atomically thin Bi_2_MoO_6_.^[^ ^223^ ^]^ A decent H_2_ yield of 214.63 µmol⋅g_cat_^−1^ was obtained through photothermal catalysis in 10 mol L^−1^ NaOH aqueous solution at 80 °C under 300 W Xe lamp irradiation for 4 h, which was much higher than that of both pure photocatalysis (almost zero yield at 25 °C) and thermal catalysis (79.29 µmol g_cat_^−1^ at 80 °C). It is thus rationalized that the photo‐thermal synergy is favorable for breaking the bottleneck of photoreforming biomass toward by maximizing the utilization of the entire solar spectrum, especially utilizing the infrared region with marked thermal effect that can not photoexcite most of semiconductors. It can be explained by boosting the activation of some reactants via thermal catalysis and decreasing the activation energy of the reaction via photocatalysis. Besides, atomically thin Bi_2_MoO_6_ benefits from abundant oxygen vacancies, rich H_2_ evolution sites, and an appropriate bandgap of 2.37 eV, thus presenting an ideal optical and structural configuration for biomass reforming. The metal‐free monolayer g‐C_3_N_4_ with ≈0.32 nm thickness was also employed for visible light‐driven reforming of cellulose and hemicellulose into H_2_ using state‐of‐the‐art cocatalyst of Pt.^[^ ^227^ ^]^ Herein, the catalytic activity of monolayer g‐C_3_N_4_ was better than that of bulk g‐C_3_N_4_ because the monolayer g‐C_3_N_4_ facilitated the separation of photogenerated charge carriers by shortening the charge diffusion distance and reducing the exciton binding energy. The hydrogen production rates for cellulose, hemicellulose, and lignin over 1.0% wt Pt/monolayer g‐C_3_N_4_ were ≈73, 60, and 21 µmol h^−1^ g^−1^, respectively, in an alkaline aqueous solution (pH 12) under visible light illumination. Owing to its uniform dispersion in solution and adjustable electronic structure, graphene oxide‐dot decorated with Pt was utilized for photoreforming of cellulose with particle size of <20 µm to H_2_ in NaOH solution, and the reaction mechanism was elucidated in detail (Figure 10b).^[^ ^228^ ^]^ Polymeric cellulose was first converted into C6 molecules via piece‐by‐piece peeling. Then the transformation of C6 molecules into C5–C1 molecules was achieved via continuous carbon elimination hydrolysis and photocatalytic oxidation. HCOO^−^ was found to be the final carbon product rather than HCO_3_^−^. It is noted that the photocatalytic oxidation of organic molecules was always accompanied by photocatalytic H_2_O splitting into H_2_. Therefore, the two reactions, i.e., biomass reforming and water splitting should be well considered during photocatalysis.

**Figure 11:**
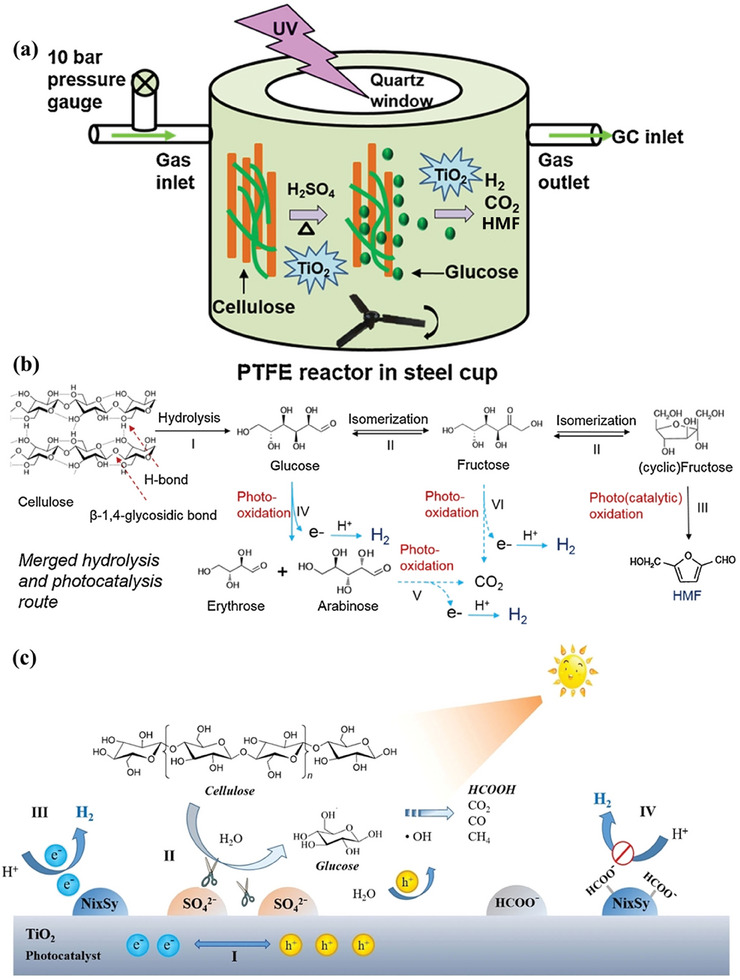
a) Illustration of experimental reactor used for photoreforming cellulose to hydrogen via the combination of photocatalysis and acid hydrolysis, and b) Proposed pathway of cellulose decomposition in combined processes of hydrolysis and photocatalysis. Reproduced with permission.^[^ ^229^ ^]^ Copyright 2018, Elsevier Inc. c) Proposed mechanism of cellulose photoreforming toward H_2_ over P25‐S‐Ni under solar irradiation. Reproduced with permission.^[^ ^231^ ^]^ Copyright 2018, Wiley‐VCH.

Apart from base pretreatment, strong acids can also favor cellulose hydrolysis. When sulfuric acid was introduced in the reaction,^[^ ^229^ ^]^ a couple of soluble small molecules (e.g., glucose, fructose, 5‐HMF) were generated by acid hydrolysis of cellulose. They can behave as ideal feedstock for in situ photocatalytic hydrogen production (**Figure** **11**a,b). A hydrogen production rate of 1.32 mmol g^−1^ h^−1^ was achieved from cellulose (size 10–1000 µm) over Pt/TiO_2_ photocatalyst in 0.6 m H_2_SO_4_ solution at 403 K under ultraviolet light irradiation. When LiBr was introduced with H_2_SO_4_ simultaneously, the optimal 62.5 wt% LiBr in low H_2_SO_4_ concentration (i.e., 0.1 mol L^−1^) solution can effectively depolymerize cellulose under mild conditions. Meanwhile, Li^+^ promotes the production of strong acidity by strong coordination with H_2_O. Simultaneously, Br^−^ contributed to wrecking the hydrogen bond of cellulose through hydrogen bonding interaction with cellulose, thus expediting the depolymerization of cellulose. The obtained soluble substrates are easily exposed to the photocatalyst, enabling H_2_ evolution at a considerable rate of 180 µmol g^−1^ h^−1^ over Pt/TiO_2_ nanoparticles.^[^ ^230^ ^]^ Of course, considering environmental accountability, it is expected to avoid the use of homogeneous acids or bases because these acids and bases are difficult to recycle, causing the generation of a large amount of wastewater. In contrast, the integration of solid acid with semiconductor material shows an eco‐friendly option, where solid acid is easily recyclable through convenient filtration or centrifugation. Typically, P25‐S‐Ni photocatalyst was prepared by simultaneous modification of TiO_2_ with chemisorbed sulfate (SO_4_^2−^) and nickel sulfide (Ni*_x_* S*_y_*) in one step for cellulose photoreforming toward H_2_ in neutral water.^[^ ^231^ ^]^ The optimum P25‐S‐Ni photocatalyst exhibited an appreciable hydrogen evolution rate of 3.02 mmol g^−1^ h^−1^, which is nearly 76‐fold higher than that of TiO_2_. The superior activity of P25‐S‐Ni photocatalyst was ascribed to the synergism of SO_4_^2−^ and Ni*_x_* S*_y_*. In this case, SO_4_^2−^ ions accelerated cellulose hydrolysis to improve the interaction between the reactant and photocatalyst, while Ni*_x_* S*_y_* served as a cocatalyst for lowering the energy barrier of H_2_ production (Figure 11c).

### 6.2 Lignin

Lignin is not only one of the three major components of lignocellulose but also a key pollutant of the pulp and paper industry. About 50 million metric tons of lignin was generated annually in the pulp and paper industry, whereas 95% of lignin is abandoned or combusted without valorization.^[^ ^232^ ^]^ Of note, it is very difficult to treat lignin from the pulp and paper industry by using conventional wastewater treatment technology on account of the stubborn structure of lignin. Utilizing lignin as the feedstock for hydrogen production offers a fantastic route for both energy and environmental topic. However, it has drawn much less attention than cellulose and hemicellulose because lignin is the most stubborn, complex, and diverse molecule among the three components of lignocellulose. Meanwhile, the dark color arising from its aromatic structure is not conducive to light harvesting by photocatalysts. Ultraviolet‐visible spectroscopy characterizations show that lignin has a broad absorption region from ultraviolet to visible light.^[^ ^233^ ^]^ Therefore, photocatalytic H_2_ generation from lignin is more challenging than that from cellulose and hemicellulose. For example, under the same experimental conditions, the H_2_ production rate of 0.26 mmol g_cat_^−1^ h^−1^ achieved from lignin with particle size of ˂ 0.25 cm over CdS/CdO*_x_* in 10 m KOH solution was nearly one order of magnitude lower than that from cellulose (2.57 mmol g_cat_^−1^ h^−1^) and hemicellulose (2.32 mmol g_cat_^−1^ h^−1^).^[^ ^69^ ^]^

**Figure 12:**
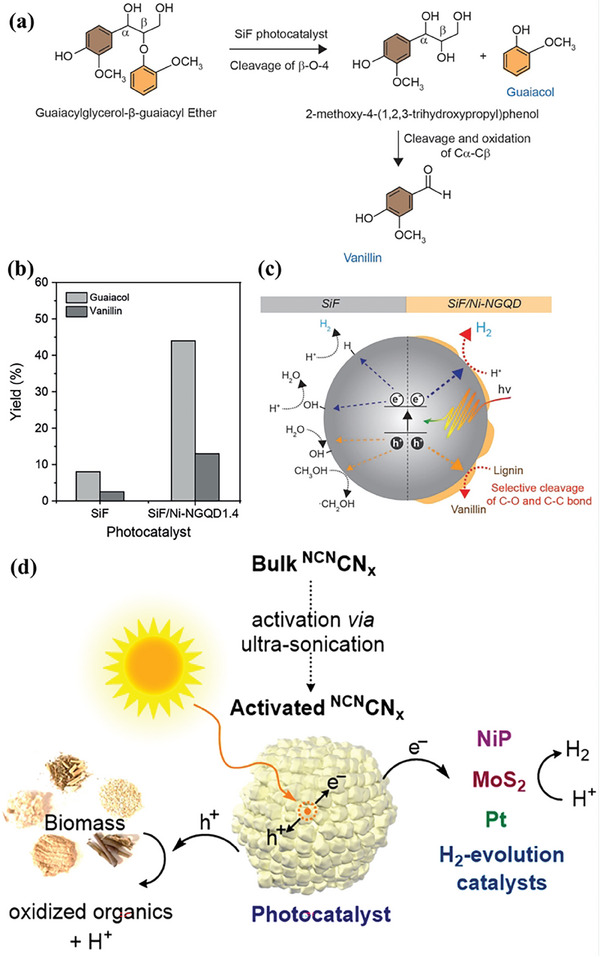
a) Selective cleavage of β‐aryl ether bond and C_α_‐C_β_ bond of lignin model compound over SiF/Ni‐NQGDs photocatalysts, b) Products of photoreforming of lignin model compound over SiF/Ni‐NQGDs and SiF, and c) Schematic illustration of lignin photoreforming over SiF/Ni‐NQGDs and SiF. Reproduced with permission.^[^ ^235^ ^]^ Copyright 2023, Wiley‐VCH. d) Proposed mechanism of photocatalytic H_2_ production from biomass over activated ^NCN^CN_x_ with various cocatalysts. Reproduced with permission.^[^ ^70^ ^]^ Copyright 2018, American Chemical Society.

To facilitate photocatalytic H_2_ evolution from lignin, 1D NiS/CdS nanocomposite was assembled by immobilizing NiS nanoparticles as co‐catalyst on CdS nanowires using a two‐step solvothermal method.^[^ ^234^ ^]^ The NiS co‐catalyst can restrain the recombination of photogenerated electrons and holes of CdS. The optimized 0.2‐NiS/CdS nanocomposite with 20 mol% of NiS loading exhibited optimal activity in the photoreforming of lignin and lactic acid in an aqueous solution with an H_2_ generation rate of 1512.4 µmol h^−1^ g^−1^, which was ≈554‐fold higher than that of sole CdS. Due to its many advantages, such as accessibility, broad light harvesting originating from its narrow bandgap (1.7 eV), and suitable conduction band edge position for hydrogen evolution reaction, silicon seems an ideal candidate for photocatalytic H_2_ production. However, there are still many limitations, including easy surface oxidation, poor stability, and low catalytic activity. To overcome these problems, modifying Si flakes (SiF) with Ni‐decorated N‐doped graphene quantum dots (Ni‐NGQDs) was proposed by Choi and coworkers. The developed Si‐based photocatalyst of SiF/Ni‐NQGDs was evaluated in coproduction H_2_ and vanillin from photoreforming of kraft lignin (**Figure** **12**).^[^ ^235^ ^]^ The results indicated that the Ni‐NGQDs enables to prevent the oxidation of silicon and promote the separation of photogenerated charge carriers (Figure 12c). More importantly, the Ni‐NGQDs as catalytic sites can selectively break the β‐aryl ether bond and C_α_‐C_β_ bond of kraft lignin (Figure 12a,b). The synergistic effect of SiF and Ni‐NQGDs resulted in a measurable H_2_ evolution rate of 14.2 mmol g^−1^ h^−1^ and a marked vanillin yield of 147.1 mg g_lignin_^−1^. In addition to photocatalysis, thermal‐assisted photocatalysis was also validated to be an effective strategy for enhancing H_2_ evolution from lignin. As a typical endeavor, a thermally radiative semiconductor hybrid of Pt/CdO_x_/CdS/SiC was developed for photo‐thermal catalysis of lignin into H_2_. A broad light absorption range from ultraviolet and visible regions holds a grand promise for the superior performance.^[^ ^236^ ^]^ For this hybrid, visible light with wavelengths exceeding 580 nm can be harvested by the black SiC, and the photo‐excited electrons and holes will be quenched via thermal radiation. As a consequence, the reaction system can be heated to 70 °C under 300 W Xe lamp irradiation, promoting the de‐polymerization of lignin in 10 m NaOH solution. Meanwhile, the photo‐generated electron transferred from SiC to CdO_x_/CdS, and then was consumed by protons to produce hydrogen. As a result, the H_2_ evolution rate reached 71.5 µmol g^−1^ h^−1^ from lignin in 10 m NaOH solution at 70 °C. Moreover, to minimize the detrimental impact on the environment, a nontoxic and metal‐free cyanamide (NCN)‐functionalized carbon nitride (CN_x_) photocatalyst (^NCN^CN_x_) was utilized for lignin photoreforming with a molecular Ni bis(diphosphine) cocatalyst (NiP) in potassium phosphate (KP_i_) solution (pH 4.5; Figure 12d).^[^ ^70^ ^]^ By ultrasonication, the aggregated ^NCN^CN_x_ can be disrupted, resulting in a 60% increase in the surface area and enhanced UV light absorption with an extended tail into the visible region. In addition to carbon nitride, carbon dot, as another rising star carbon‐based material, has also appeared as a promising candidate for photocatalytic reforming of lignin toward H_2_.^[^ ^237^ ^]^ Owing to their marked photophysical properties with a high proportion of long‐lived charge carriers and the availability of a reductive and an oxidative quenching pathway, carbon dots derived from biomass were employed as photon absorbers for photoconverting lignin into H_2_ with a Ni cocatalyst.^[^ ^238^ ^]^ Importantly, because of the outstanding solubility, carbon dots can be highly dispersed in the reaction system, which can effectively interact with the insoluble lignin. A H_2_ yield of 7.8 µmol was obtained under simulated solar light illumination (100 mW cm^−2^) at 25 °C for 24 h. Based on the discussions above, it is rationalized that lignin can be a viable and cost‐effective feedstock for photocatalytic H_2_ production.

### 6.3 Monosaccharides

Monosaccharides are the basic units of lignocellulose. Typically, monosaccharides include both C_5_ sugars (e.g., xylose, arabinose) and C_6_ sugars (e.g., glucose, fructose, galactose, and mannose). They can be facilely obtained from either edible or inedible biomass by hydrolysis. As a matter of fact, monosaccharides are widely available in plants and animals, and even some industrial wastewater also contains abundant monosaccharides. Owing to their abundance on the earth, monosaccharides are regarded as a huge hydrogen reservoir. They have been extensively investigated as model substrates for biomass photoreforming (Table 3). As a presentative, using noble metals‐loaded carbon nanotube‐titanium dioxide (CNT‐TiO_2_) composite material as the photocatalysts, various monosaccharides were employed as feedstock for photocatalytic hydrogen generation.^[^ ^239^ ^]^ The efficiency for hydrogen production was associated with the structural complexity of the monosaccharides tested. The H_2_ evolution rate declined in the order of arabinose > glucose > fructose > cellobiose as the structural complexity of monosaccharide increased.

As one of the most plentiful biomass‐based monosaccharides, glucose with abundant hydroxyl groups can effectively capture and consume photo‐generated holes to release H^+^. The released protons are then converted toward H_2_ with photo‐generated electrons. As discussed above, the cocatalyst played a crucial role in the photocatalytic reforming of biomass. In an endeavor of glucose photoreforming with various metals‐decorated TiO_2_ (Pt, Rh, Ru, Ir, Au, Ni, Cu),^[^ ^240^ ^]^ the activity of H_2_ followed the order of Rh > Pt ≈ Cu ≈ Ni > Au > Ru > Ir. The Rh/TiO_2_ catalyst showed the highest H_2_ activity with the lowest by‐product CO concentration (30 ppm). The extremely low CO concentration in the H_2_ production system is critical for the practical operation of the H_2_ fuel cell because CO can easily deactivate the membrane electrode assembly (MEA) of a fuel cell. The effect of experimental conditions such as reaction temperature on the reaction was also studied.^[^ ^241^ ^]^ It is observed that high temperature (60‐80 °C) facilitates hydrogen production by improving the access of glucose molecules to the active sites of the photocatalyst. However, higher glucose concentration is not conducive to hydrogen generation, which can be ascribed to the suppressed consecutive reaction steps. In view of its tunable structure, excellent stability, high carrier mobility, broadband light absorbance, and environmental friendliness, graphene oxide (GO), as a typical metal‐free semiconductor, also appears as an ideal option for photocatalytic hydrogen generation from monosaccharides.^[^ ^242^ ^]^ However, the rich vacancy defects in GO were detrimental to the reaction. Interestingly, co‐doping of S and N in GO dots (SNGODs) was discovered to be capable of repairing the vacancy defects, narrowing the bandgap, and prolonging the lifetime of photogenerated carriers of GODs.^[^ ^243^ ^]^ Consequently, by coupling with state‐of‐the‐art HER cocatalyst of Pt, the as‐prepared SNGODs showed a considerable activity of 164 mmol h^−1^ g^−1^ for H_2_ generation from glucose aqueous solution illuminated by a 300 W xenon lamp at 35 mW cm^−2^. The apparent quantum yields of SNGODs reached 7.4% under 420 nm monochromatic irradiation.

**Figure 13:**
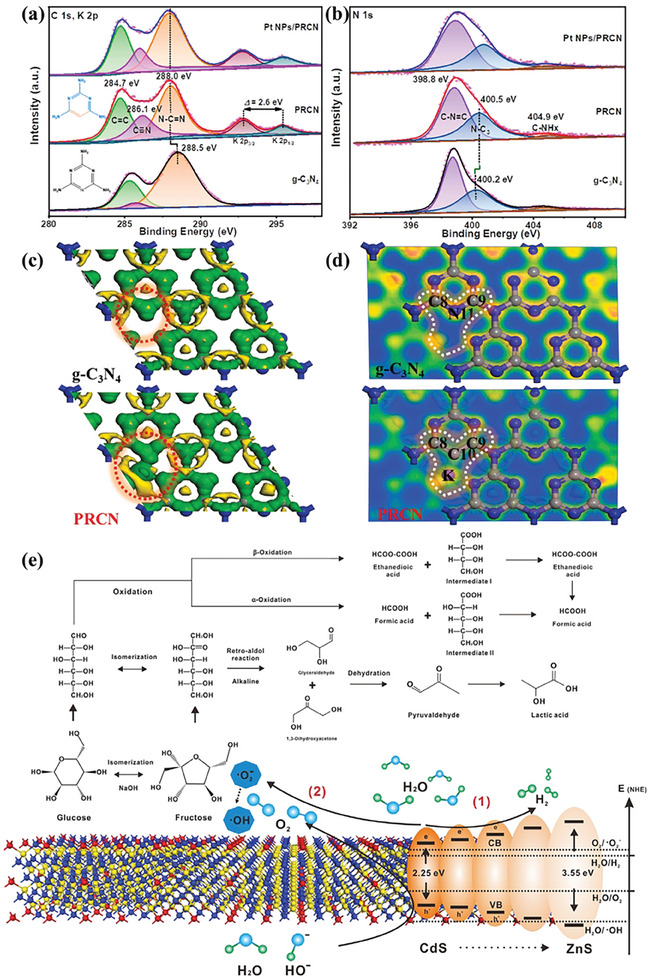
High‐resolution C 1s (a) and N 1s (b) XPS spectra of g‐C_3_N_4_, PRCN, and Pt NPs/PRCN, c) charge density difference (yellow/green: electron cloud density of N/C), and d) orbital population of g‐C_3_N_4_ and PRCN (Blue: N, Gray: C, Purple: K). Reproduced with permission.^[^ ^245^ ^]^ Copyright 2022, Elsevier Inc. e) proposed reaction mechanism for photocatalysis of glucose into H_2_ and lactic acid. Reproduced with permission.^[^ ^247^ ^]^ Copyright 2021, Cell Press.

In concurrent production of H_2_, glucose can be oxidized into various high‐value‐added chemicals (such as arabinose, fructose, lactic acid, formic acid, etc.) by the photogenerated holes in the presence of suitable cocatalysts. As an attempt, the carbon quantum dots (CQDs)/TiO_2_ composites were assembled for photoreforming glucose aqueous solution toward H_2_ and arabinose at the same time. In the designed hybrid, CQDs functioned as a photosensitizer and a co‐catalyst owing to their outstanding optical properties and high electrical conductivity.^[^ ^244^ ^]^ The P25‐CQDs‐H‐2 showed an appreciable arabinose selectivity of ∼75% and a hydrogen production rate of 2.43 mmol h^−1^ g^−1^. The mechanism investigation illustrated that ^•^O^2‐^ could be generated by the interaction of oxygen with photo‐generated electrons while highly active hydroxyl radical species (^•^OH) was generated from water oxidation by photo‐generated holes. These two kinds of active species are critical for promoting the activation and transformation of fructose by the direct C_1_‐C_2_ α‐scissions, thus leading to a considerable H_2_ activity and arabinose selectivity. Isomerization of glucose into fructose is a vital step for the conversion of glucose into value‐added chemicals and fuels. Apart from the widely studied TiO_2_‐based materials, the combination of polymeric red carbon nitride (PRCN) with Pt cocatalyst was also conducted for the photoreforming of glucose with co‐production of H_2_ and fructose.^[^ ^245^ ^]^ It is noted that the incorporation of C and K heteroatoms into PRCN enabled the achievement of a narrow band‐gap with absorption edge to the near‐infrared region and the transient redistribution of photo‐generated charges, thus benefitting the reaction. As presented in **Figure** **13**a–d, the active sites of PRCN were characterized by X‐ray photoelectron spectroscopy (XPS) characterizations and density functional theory (DFT) calculations. It was validated that the electron‐rich C heteroatom acted as the Lewis base site for the deprotonation of the ‐O(2)H group of glucose while the positive K site performed as the Lewis acid site to activate the aldehyde group of glucose. This strategy promoted the isomerization of glucose into fructose, and conversion of about 40% was achieved with an approximately 58% selectivity. When state‐of‐the‐art Pt was utilized as a cocatalyst for boosting the electron‐hole separation and lowering the reaction energy barrier, a considerable H_2_ generation rate of 72 µmol∙g^−1^∙h^−1^ was obtained.

**Figure 14:**
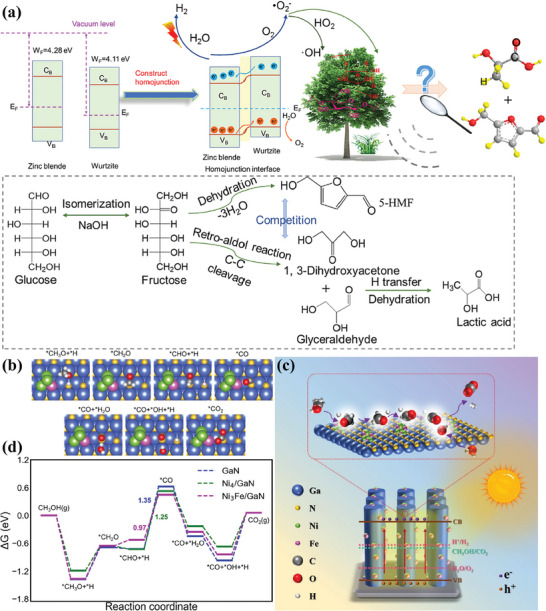
a) The possible reaction route of glucose photoreforming over Zn_0.3_Cd_0.7_S. Reproduced with permission.^[^ ^248^ ^]^ Copyright 2023, Elsevier Inc. b) Adsorption configurations for various intermediates of methanol photoreforming toward H_2_ on Ni_3_Fe/GaN, c) Schematic illustration of methanol photoreforming to generate H_2_ using NiFe NPs/GaN NWs/Si architecture, and d) Energy profiles for photoreforming of methanol on GaN(101̅0), Ni_4_/GaN, and Ni_3_Fe/GaN. Reproduced with permission.^[^ ^160^ ^]^ Copyright 2023, the American Chemical Society.

Compared to the pure generation of H_2_ from biomass photoreforming, the coproduction of H_2_ and value‐added chemicals makes the process more economically competitive. Lactic acid is one of the most important bio‐chemicals with a broad range of applications in biodegradable plastic, food, pharmaceuticals, and so on.^[^ ^246^ ^]^ Approximately 70% of lactic acid originates from the fermentation of carbohydrates (e.g., sucrose, starch). However, the fermentation suffers from strict operation conditions, low efficiency, and concurrent formation of a large amount of waste. Thus, the photoreforming of glucose into lactic acid with the co‐generation of hydrogen shows an appealing strategy for converting solar energy into storable fuels and value‐added chemicals. Fabrication of an efficient photocatalyst is at the core of this grand topic. Both suitable redox potentials and inhibited charge recombination are two important metrics for a rational photocatalyst. The construction of homojunction in photocatalysts was verified to be a viable means to achieve this goal.^[^ ^247^ ^]^ As reported by Zhao and co‐workers, the homojunction built‐in Zn*_x_* Cd_1‐_ *_x_* S can not only regulate the redox potential of the photocatalyst but also ensure the timely transfer of photo‐generated charges (Figure 13e). Consequently, a H_2_ production rate of 690 µmol g^−1^ h^−1^ was achieved with a glucose conversion of 90% and a lactic acid selectivity of about 87% over the optimized Zn_0.6_Cd_0.4_S photocatalyst under visible light irradiation. Inspired by this discovery, marigold‐like Zn_0.3_Cd_0.7_S was fabricated to further improve the yield of H_2_ and lactic acid.^[^ ^248^ ^]^ Ascribing to the marigold‐like morphology and homojunction, the Zn_0.3_Cd_0.7_S photocatalyst demonstrated high access of the feedstock to the reaction sites and effective e^−^/h^+^ separation, respectively. As a consequence, an appreciable H_2_ evolution rate of 13.64 µmol g^−1^ h^−1^ was obtained with a lactic acid yield of 76.80% in a mixed basic solution of H_2_O and dimethyl sulfoxide (DMSO), which was 272.80 and 19.21 folds higher than that of pristine ZnS and CdS in H_2_ generation, respectively. The reaction mechanism was also studied. As illustrated in **Figure** **14**a, ^•^O_2_^−^ was the vital photoactive species for the conversion of glucose into lactic acid and H_2_ was produced by reducing H^+^ with the use of photoexcited electrons in water. To avoid toxic Cd‐containing photocatalyst, Pt‐C_3_N_4_ photocatalysts were synthesized by embedding Pt nanoparticles in nontoxic polymeric carbon nitride (C_3_N_4_) via the pyrolysis of a preorganized supramolecular assembly. The photocatalytic activity was evaluated by photoreforming glucose into H_2_ and lactic acid in 10 M NaOH aqueous solution.^[^ ^249^ ^]^ The immobilization of Pt on the surface of C_3_N_4_ not only reduces the activation energy of hydrogen production from water reduction, but also promotes photogenerated electron transfer and cleavage of the C‐C bond of the fructose derived from glucose isomerization. As a result, the Pt‐C_3_N_4_ photocatalyst with 0.21 wt % Pt nanoparticles illustrated a measurable H_2_ rate of 3.39 mmol g^−1^ h^−1^ under irradiation of a 427 nm LED light, which is 9.2 times higher than that of the pristine C_3_N_4_. Meanwhile, the yield of high‐value‐added lactic acid reached up to 86%. Again, the concurrent production of H_2_ and value‐added chemicals makes photocatalytic biomass reforming economically competitive, thus showing a grand promise for photocatalytic reforming of biomass.

Xylose, a key component of hemicellulose, was also considered as a promising hydrogen resource. A curly‐like carbon nitride nanosheet (HCN‐NEA) with abundant mesopores decorated with Pt was used to produce hydrogen from xylose photoreforming.^[^ ^250^ ^]^ In this work, the in‐plane surface dyadic heterostructure of HCN‐NEA formed by fold structures was beneficial for extending visible light absorption and for suppressing e^−^/h^+^ separation. As a result, an appreciable hydrogen evolution rate of 4092 µmol h^−1^ g^−1^ was obtained under visible light with an apparent quantum efficiency (AQE) of 7.87% (λ = 420 nm), which was 15.6‐fold that of bulk carbon nitride. Besides, as reported by Wang and co‐workers, the grafting functional group is one viable strategy to tailor the bandgap structure of C_3_N_4_ for improving photocatalytic performance.^[^ ^144^ ^]^ Specifically, after grafting various contents of ethyl alcohol groups (NA) in carbon nitride (UCN), a new discrete energy level in the bandgap was formed. Consequently, the visible absorption region of UCN‐NA_x_ was extended from 450 to 650 nm. Meanwhile, the ethyl alcohol group, serving as an electron donor, can adjust the electron distribution to generate an internal electric field, which is beneficial for promoting carrier's separation and for expanding the carrier's lifetime. The ethyl alcohol group of UCN‐NA_x_ is conducive to the absorption of d‐xylose via hydrogen bonding interaction, thus facilitating the interaction between catalytic active site and d‐xylose. Benefitting from the above merits, the H_2_ yield of the optimal UCN‐NA_0.5_ (136.9 µmol) was ≈5.93 times that of pristine UCN (23.1 µmol) for photocatalytic reforming of d‐xylose in 5 m NaOH aqueous solution under a 5 W white LED irradiation for 4 h.

Meanwhile, xylose is also an alternative feedstock for the synthesis of lactic acid coupled with H_2_ evolution. The concurrent generation of lactic acid and H_2_ was realized by photocatalytic reforming of xylose over RuP_2_/Ti_4_P_6_O_23_@TiO_2_‐7.^[^ ^252^ ^]^ For the nanostructured TiO_2_ platform, the incorporation of RuP_2_ and Ti_4_P_6_O_23_ greatly broaden the light absorption range and provided abundant active sites for the coproduction of lactic acid and H_2_. Moreover, it also facilitated the separation of photo‐generated electrons and holes. Together, the improved properties afforded H_2_ generation at a rate of 16.3 mmol g^−1^ h^−1^ and lactic acid production at a rate of 2.41 mmol g^−1^ h^−1^ from xylose aqueous solution, respectively.

## 7 Photoreforming of Biomass‐Derived Liquid H_2_ Carriers

The safe storage and transportation of H_2_ remains one key challenge to its widespread application as a global energy carrier.^[^ ^253^ ^]^ On‐site hydrogen evolution from LOHCs has emerged as an innovative strategy for addressing this critical issue.^[^ ^254^ ^]^ Biomass‐derived alcohols (e.g., methanol, ethanol, glycerol), and formic acid have been identified as promising LOHCs, due to their unique advantages of low cost, renewability, high hydrogen content, easy transportation, and low toxicity.^[^ ^255^ ^]^ Short‐chain alcohols, as common biomass derivatives, are usually generated as targets or byproducts from biomass conversion. For instance, glycerol is a typical by‐product of the biodiesel industry with an annual global yield of almost 3 million tons that is estimated to be approximately sixfold higher than the current market demand.^[^ ^256^ ^]^ Meanwhile, ethanol can be massively obtained by the biological fermentation of sugars with an annual yield of 110 billion liters globally.^[^ ^257^ ^]^ Pyrolysis oils also contain a wide variety of alcohols including methanol, and ethanol. These bio‐derived alcohols are promising substrates for photocatalytic hydrogen production owing to their high hydrogen content and diverse functional groups with high reactivity. In addition, HCOOH derived from biomass is also a suitable LOHC for on‐site hydrogen evolution.^[^ ^258^, ^259^ ^]^ In the following, the advances in on‐site hydrogen production from photocatalytic reforming of bio‐derived LOHCs will be introduced.

### 7.1 Methanol for Solar‐Driven H_2_ Generation

Although methanol is frequently prepared from fossil resources via the well‐known Fischer–Tropsch process, methanol can be also produced from biomass via thermochemical and biochemical processes.^[^ ^260^ ^]^ Benefitting from the characteristics of high H_2_ content (99 kg m^−3^), liquid phase under ambient condition, highly compatible with the existing storage and transportation infrastructure of fossil fuels, methanol is considered as an ideal liquid organic hydrogen carrier.^[^ ^261^ ^]^ From the viewpoint of molecular structure, methanol is C–C bond‐free and exhibits a high hydrogen‐to‐carbon ratio. It is thus highly favorable for hydrogen evolution without marked coking during methanol photoreforming.^[^ ^262^ ^]^ Notably, the Gibbs free energy of CH_3_OH + H_2_O → 3H_2_ + CO_2_ is as low as 9.3 kJ mol^−1^, which is much lower than that of 237 kJ mol^−1^ for water splitting. Hence, from the thermodynamic point of view, methanol is an ideal platform molecule of hydrogen carrier compared to water, which is highly favorable for releasing H_2_ by integrating with the storage of intermittent solar energy. As a result, the economy based on methanol was proposed in the late 1990s and has received tremendous attention. The development of efficient photocatalytic systems for releasing H_2_ from methanol is critical for the success of the proposition. In recent years, considerable efforts have been devoted to photocatalytic hydrogen production from methanol (**Table** **4**). As a representative, Ni‐decorated CdS (Ni/CdS) was assembled for methanol photoreforming in the presence of NiCl_2_.^[^ ^263^ ^]^ In particular, the adsorption of Ni^2+^ sites results in a positively charged surface. The surface charge‐induced activation of Ni/CdS thereby contributed to great enhancements in both the activity and stability by promoting the adsorption of CH_3_OH and desorption of HCHO. Under the optimized experimental conditions, an incredible photon‐to‐hydrogen efficiency of 95% was recorded at 405 nm. We have discovered that the activity of nickel (Ni) could tuned by coupling with the secondary metal of Fe for photocatalytic methanol reforming toward H_2_.^[^ ^160^ ^]^ Operando spectroscopy characterization, in combination with DFT calculations, reveals that the synergistic effect of Ni and Fe significantly reduced the energy barrier of *CHO → *CO + *H by stabilizing the *CO intermediate (Figure 14b,d), which is the potential‐limiting step for hydrogen production from methanol. Meanwhile, the interface of GaN NWs/Si facilitated the activation of methanol to generate *CH_3_O since Ga and N atoms in GaN strongly bonded the O and H atoms in the hydroxyl of methanol, respectively. Moreover, gallium nitride nanowires (GaN NWs) supported by silicon wafers (GaN NWs/Si) with ordered 1D morphology offered an appropriate semiconductor platform for decoupling light absorption, charge carriers separation, and active site exposure. (Figure 14c). Together, NiFe NPs/GaN NWs/Si presents an efficient and affordable architecture for light‐driven H_2_ production from methanol at a rate of 61.2 mmol g^−1^ h^−1^ under concentrated light illumination of 5 W cm^−2^, which outperformed many noble metals tested (e.g., Ru, Pd, Ir, and Au). Most recently, we found that the synergy of NiCo nanoclusters with GaN NWs enabled switching the potential‐limiting step from *CHO → *CO to *CH_3_O → *CH_2_O during light‐driven on‐site hydrogen production from methanol‐reforming, thus resulting in greatly reducing the activation energy of methanol dehydrogenation.^[^ ^264^ ^]^ Simultaneously, in combination with photothermal effect, a considerable H_2_ production rate of 5.62 mol g_cat_^−1^ h^−1^ with a high turnover frequency of 43 460 h^−1^ is obtained under concentrated light of 5 W cm^−2^. Due to the outstanding resistance to sintering and coking, NiCo NPs/GaN NWs/Si demonstrates a decent turnover number of >16 310 000 mol H_2_ per mol NiCo during a long‐term operation of 600 h. What is more, to validate the practical viability, an outdoor testing under natural concentrated sunlight was conducted.

**Table 4:** Representative examples of photocatalytic H_2_ production from methanol.

Photocatalysts	Light source	H_2_ evolution rate	Solar‐chemical conversion efficiency	Lifetime	Reference
Zn_1.3_Cu_98.7_	Focused solar light 788 mW cm^−2^	328 mmol g^−1^ h^−1^	1.2%	–	[73]
NiFe NPs/GaN NWs/Si	300 W Xe lamp, concentrated light intensity 5 mW cm^−2^	61.2 mmol g^−1^ h^−1^	–	10 h	[165]
1% FLG/TiO_2_	31.9 mW cm^−2^	265 µmol g^−1^ h^−1^	–	–	[265]
TiO_2_/Cu50	300 W Xe lamp, 100 mW cm^−2^	17.8 mmol g^−1^ h^−1^	–	–	[266]
Cu‐Zn‐Ti oxide	100 mW cm^−2^, 210 °C	76.2 mmol g^−1^ h^−1^	–	–	[270]
Cu/ZnO/Al_2_O_3_	11 KW cm^−2^, 240 °C	3.45 µmol g^−1^ s^−1^	–	–	[271]
Cu/Zn/Zr (70:23:7) oxide nanocatalyst	300 W Xe lamp, 16 suns	1.51 mL g^−1^ s^−1^	45.6%	–	[272]
10%Cu/CeO_2_	300 W Xe lamp, 250 °C	36 mL g^−1^ min^−1^	–	–	[273]
Bi_2_Te_3_/Cu‐ CuO_x_/ZnO/Al_2_O_3_	Xe lamp, 1 sun, 305 °C	310 mmol g^−1^ h^−1^	30.1%	20 day	[274]
CuO/ZnO/ZrO_2_	700 mW cm^−2^	–	10.7%	–	[275]

**Figure 15:**
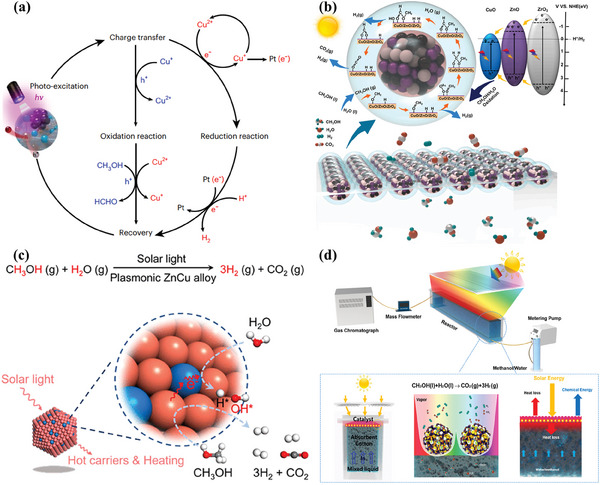
a) The possible reaction mechanism of CH_3_OH photoreforming toward H_2_ and HCHO over PtCu‐TiO_2_ photocatalyst. Reproduced with permission.^[^ ^75^ ^]^ Copyright 2023, Springer Nature. b) Schematic illustration of solar‐driven methanol reforming into hydrogen via synergetic photo‐ and thermocatalysis. Reproduced with permission.^[^ ^272^ ^]^ Copyright 2021, Elsevier Inc. c) Triggering water and methanol activation for solar‐driven H_2_ production over plasmonic ZnCu alloy via photo‐thermal‐catalysis. Reproduced with permission.^[^ ^73^ ^]^ Copyright 2021, the American Chemical Society. d) Schematics of the outdoor flow solar‐heating thermocatalysis of methanol reforming into H_2_. Reproduced with permission.^[^ ^275^ ^]^ Copyright 2023, Elsevier Inc.

What is more, combining the merits of graphene and TiO_2_, the few‐layer graphene/TiO_2_ composites (FLG/TiO_2_) were integrated for photocatalytic H_2_ production from methanol.^[^ ^265^ ^]^ The activities of all the composites tested far outstripped individual FLG and TiO_2_. The FLG/TiO_2_ composite with 1 wt% FLG showed a maximum H_2_ evolution rate of 265 µmol h^−1^ g^−1^, which was almost 3 times that of the bare TiO_2_. Large surface area, efficient charge carrier separation, and wide visible light responsiveness contributed to the enhanced activity. What is more, selective dehydrogenation of CH_3_OH toward H_2_ and high‐value HCHO provides an effective strategy for the coproduction of high‐purity H_2_ and value‐added chemicals. As a successful attempt, Cu nanoclusters were uniformly dispersed on (001) facets of TiO_2_ nanosheets (TiO_2_/Cu) by calcination of copper‐based metal‐organic framework (HKUST‐1) supported TiO_2_.^[^ ^266^ ^]^ Among all the samples tested, TiO_2_/Cu_50 exhibited a marked H_2_ formation rate of 17.8 mmol h^−1^ g^−1^ with 16.4% quantum efficiency at 365 nm within a 16 h test. Theoretical investigations reveal that strong interaction between Cu nanocluster and TiO_2_ can reduce the energy barrier of CH_3_OH dehydrogenation and lower the Gibbs free energy for HCHO and H_2_ desorption, which is beneficial for improving the activity and selectivity of H_2_ generation. Most recently, as a benchmark advance, an atomic‐level catalyst design strategy was proposed to develop the PtCu‐TiO_2_ photocatalyst for CH_3_OH dehydrogenation to H_2_ and HCHO.^[^ ^75^ ^]^ Using the synergy between single Cu atoms and Pd nanodots, the developed sandwich PtCu‐TiO_2_ photocatalyst illustrated a remarkable H_2_ evolution rate of 2383.9 µmol∙h^−1^ with a high apparent quantum efficiency of 99.2%, in the concurrent formation of value‐added chemical formaldehyde with 98.6% selectivity, achieving a nearly zero‐carbon‐emission process. During the reaction process, the Cu single atoms exhibited dual functions: 1) Cu^2+^ serves as an electron bridge to transfer electron to Pt for the reduction of H^+^ to H_2_; 2) Cu^+^ extracts photo‐generated holes to form the Cu^2+^ for selective oxidation of CH_3_OH to HCHO (**Figure** **15**a). The dual‐site single‐atom catalysts and synergy between single atoms and nanodots thus led to a very low activation energy (13.2 kJ∙mol^−1^), which primarily contributed to the benchmark performance of photocatalytic dehydrogenation of CH_3_OH toward H_2_ and CH_2_O.

As discussed above, photothermocatalysis has drawn growing research interest since the synergy of charge carriers and photo‐induced heat is promising for significantly improving the activity. Meanwhile, photothermal catalysis enabled the reduction in the reaction temperature compared with thermocatalysis.^[^ ^267^ ^]^ However, compared to both thermocatalysis and photocatalysis, the mechanism of photothermocatalysis remains largely unknown. As a common observation, by using a Cu‐modified TiO_2_ catalyst, the photothermal catalytic activity was superior to that of either thermocatalysis or photocatalysis.^[^ ^268^ ^]^ Photothermal reforming of methanol showed a marked H_2_ production rate of 60 mmol h^−1^ g^−1^ at 200 °C under light illumination, which is seven times higher than that of photocatalysis at 20 °C. In situ FTIR characterizations uncovered that CO could have evolved from the surface formate species; and increasing the temperature favored water gas shift reaction, thus leading to a measurable hydrogen activity under high reaction conditions.^[^ ^269^ ^]^ Differentiating from thermo‐assisted photocatalysis, it is reported that photon energy induced low‐temperature activation of Cu–Zn–Ti oxide solid solution catalyst for methanol steam reforming. The photo‐assisted thermal catalytic activity over Cu‐Zn‐Ti oxide was six times that of thermocatalysis at 210 °C.^[^ ^270^ ^]^ Meanwhile, Lu and coworkers reported that, for methanol steam reforming using Cu/ZnO/Al_2_O_3_ catalyst, the synergistic effect of light and heat from solar energy enabled decreasing the reaction temperature by more than 10 °C at 188 °C compared with the thermochemical reaction, accompanied by an increased hydrogen production of 32.9%.^[^ ^271^ ^]^ Mechanism investigations demonstrated that hot electrons induced by sunlight irradiation on Cu and ZnO facilitated the generation of the critical intermediates of hydroxyl groups during the thermal catalytic process, thus greatly improving the catalytic activity.

Copper (Cu) as a low‐cost plasma metal can effectively harvest and convert solar energy into energetic “hot carriers” and heat by the LSPR effect. Thus, Cu has received great attention in the construction of a rational architecture for photo‐thermal‐catalytic methanol reforming toward H_2_. For instance, the composite Cu/Zn/Zr oxide nanocatalysts were synthesized and their performance of the synergetic photo‐ and thermocatalysis for methanol reforming was H_2_ was evaluated (Figure 15b).^[^ ^272^ ^]^ Herein, the as‐designed photocatalyst was excited by high‐energy photons to generate photogenerated electrons for photocatalysis while low‐energy photons would be converted into heat via phot‐thermal effect for methanol evaporation and thermocatalysis. Because of the synergetic effect of photocatalysis and thermocatalysis, a considerable hydrogen evolution rate of 1.51 mL g^−1^ s^−1^ was achieved with a solar‐chemical conversion efficiency of 45.6% under irradiation of 16 suns. Furthermore, by simultaneous utilization of the plasmonic effect of copper and the rich oxygen vacancies of CeO_2_, a photothermal catalyst of Cu/CeO_2_ was developed for methanol steam reforming.^[^ ^273^ ^]^ Again, the synergism of photo and thermal effects played a crucial role in greatly improving the activity and stability of Cu/CeO_2_. The proper 10%‐Cu/CeO_2_ catalyst showed enhanced light absorption and a strong interaction between Cu and CeO_2_, which leads to a H_2_ production rate of 36 mL g^−1^ min^−1^ with 95.5% conversion at 250 °C.

Unlike photoexciting a semiconductor, LSPR shows another appealing approach for photo‐thermal‐catalytic methanol reforming by utilizing energetic “hot” carriers and heat. As a representative, a plasmonic ZnCu alloy, where Zn atoms were uniformly dispersed on the Cu surface, was assembled for solar‐driven methanol steam reforming (Figure 15c).^[^ ^73^ ^]^ The optimal alloy of Zn_1.3_Cu_98.7_ showed an impressive H_2_ production rate of 328 mmol g_cat_^−1 ^h^−1^ and a solar energy conversion efficiency of 1.2% under focused simulated sunlight irradiation with the light intensity of 788 mW cm^−2^. In the alloy, Zn atoms enabled the substantial reduction in the activation energy barrier of water dissociation and accelerated the trap and transfer of hot electrons. Meanwhile, due to the LSPR effect, Cu can efficiently harness and transform solar energy into energetic “hot carriers” and heat. The synergistic effect of Zn and Cu thus endows Zn_1.3_Cu_98.7_ with high activity.

In addition to the aforementioned photo‐thermal‐synergetic catalysis, photo‐driven thermocatalysis shows an ideal alternative to effectively utilize solar energy for methanol reforming toward H_2_, where light is utilized to only generate heat without the generation of photo‐excited charge carriers. For photo‐driven thermocatalysis, it is in principle a pure thermocatalysis process. Therefore, exploring an efficient photo‐thermal material that enables the achievement of a high localized temperature under light irradiation is at the core. The Bi_2_Te_3_ with a narrow‐bandgap is capable of almost fully absorbing the solar spectrum and efficient conversion of the absorbed sunlight into heat via electron‐phonon and electron‐electron scattering.^[^ ^274^ ^]^ Thus, a black photo‐driven thermal material of Bi_2_Te_3_ was coupled with the infrared insulating material of Cu to elevate solar heating temperature. The heterostructure of Bi_2_Te_3_/Cu with a 100 nm‐thick Bi_2_Te_3_ layer could maximize sunlight absorption and minimize heat loss. Consequently, the temperature of Bi_2_Te_3_ increased from 93 to 317 °C under 1 sun irradiation by realizing the cooperation of 89% solar absorption and 5% infrared radiation. It was further utilized as a heating device for photo‐driven thermocatalysis of methanol steam reforming over the CuO*_x_*/ZnO/Al_2_O_3_ catalyst. Herein, Bi_2_Te_3_/Cu enabled to balance sunlight absorption and thermal radiation. Consequently, the system can be heated up to 305 °C under 1 sun irradiation. Such a hierarchical solar‐heating catalysis system exhibited a remarkable H_2_ production rate of 310 mmol H_2_ g_cat_^−1^ h^−1^ and a measurable solar‐to‐hydrogen efficiency of 30.1% within 20 days. To explore the practical prospects of solar‐heating thermocatalysis, an outdoor solar‐driven thermocatalysis system was developed for H_2_ production from methanol (Figure 15d).^[^ ^275^ ^]^ The CuO/ZnO/ZrO_2_ nanocatalyst exhibits a solar full‐spectrum absorption of 91.4%. By optimizing liquid feeding rate, water/methanol molar ratio, and light intensity, an appreciable solar‐to‐chemical conversion efficiency of 10.7% was achieved.

### 7.2 Ethanol for Photocatalytic H_2_ Generation

**Figure 16:**
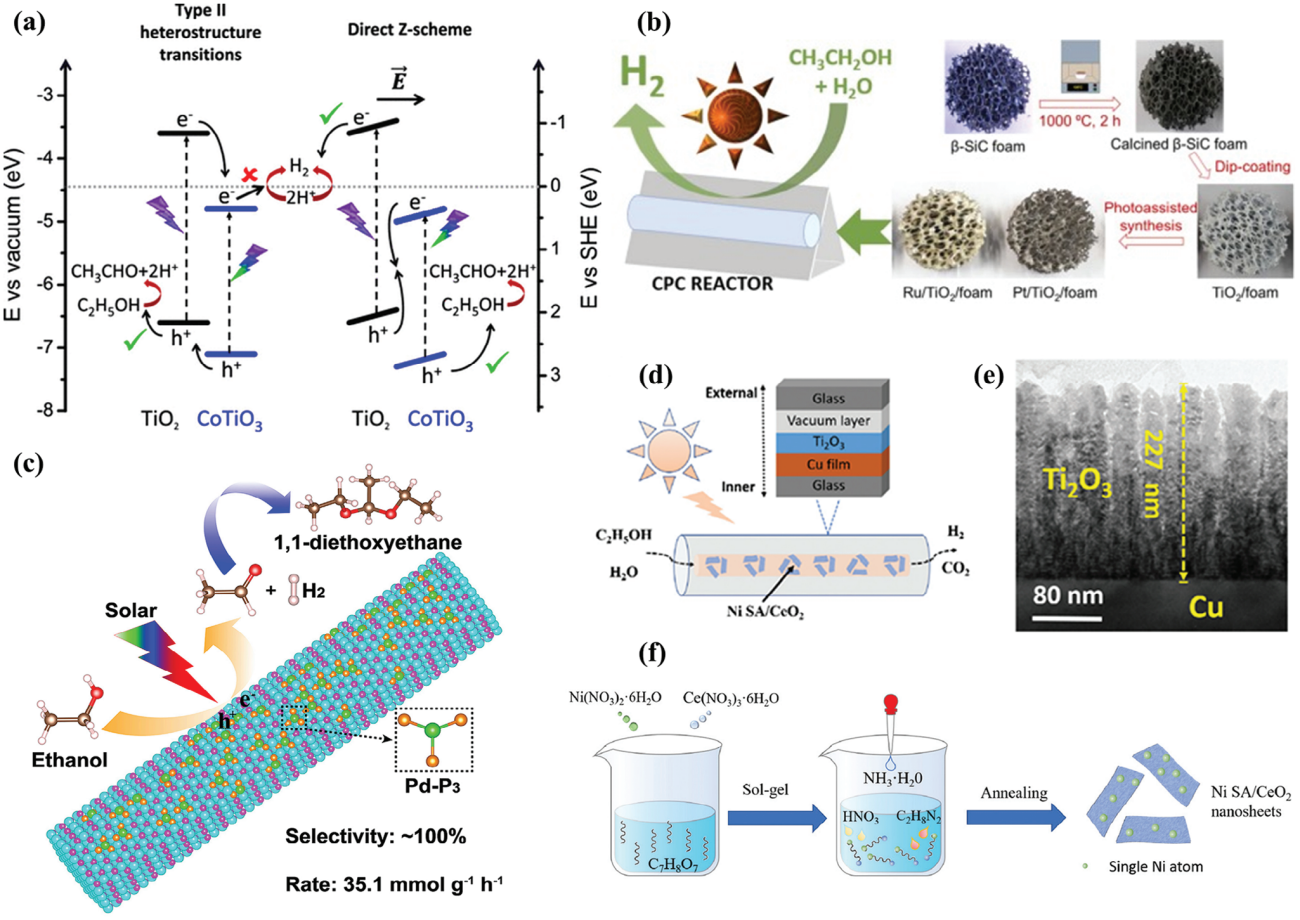
a) Schematic demonstration of the relative energy levels of TiO_2_ and CoTiO_3_ and a charge migration mechanism between TiO_2_ and CoTiO_3_ in a type II heterostructure and a direct Z‐scheme. Reproduced with permission.^[^ ^282^ ^]^ Copyright 2021, the American Chemical Society. b) Schematic illustration of solar hydrogen production from ethanol‐water over metal/TiO_2_ photocatalysts supported on β‐SiC alveolar foams. Reproduced with permission.^[^ ^284^ ^]^ Copyright 2023, Elsevier Inc. c) Schematic illustration of photoreforming of ethanol into DEE and H_2_ over PdPSA‐CdS. Reproduced with permission.^[^ ^295^ ^]^ Copyright 2021, Cell Press. d) Schematic demonstration of the photothermal device of Ni SA/CeO_2_‐Ti_2_O_3_/Cu for solar‐driven H_2_ evolution from ethanol, e) TEM cross‐section of a Ti_2_O_3_/Cu film, and f) Schematic of the synthesis process for the Ni SA/CeO_2_ nanosheets. Reproduced with permission.^[^ ^292^ ^]^ Copyright 2022, The Royal Society of Chemistry.

Ethanol is the predominant product of the fermentation of biomass with an annual yield of ≈10^11^ liters by the equation of C_6_H_12_O_6_ → 2C_2_H_5_OH + 2CO_2_.^[^ ^276^ ^]^ Owing to the distinguished characteristics of renewability, nontoxicity, convenient transportation, and high hydrogen content (13.3%)^[^ ^277^, ^278^ ^]^ bioethanol is also considered as an ideal LOHCs for photocatalytic H_2_ generation (**Table** **5**). In principle, hydrogen can be produced from ethanol via various routes as follows: 1) bioethanol dehydrogenation (CH_3_CH_2_OH(g) → CH_3_CHO(g) + H_2_(g), Δ*H*_r_^0^_298_ = 68 kJ mol^−1^) is an endothermic reaction. It provides an opportunity of producing value‐added chemicals from ethanol reforming in the concurrent generation of green H_2_ via selectively activating O–H and C–H bond. It is noted that acetaldehyde is a versatile chemical precursor with wide applications; 2) bioethanol steam reforming (CH_3_CH_2_OH(g) + 3H_2_O(g) → 6H_2_(g)+ 2CO_2_(g), Δ*H*_r_^0^_298_ = 173.5 kJ∙mol^−1^) is a high endothermic reaction, resulting in the requirement of massive external energy supply to drive this reaction; 3) bioethanol partial oxidation (CH_3_CH_2_OH + 1.5O_2_ → 3H_2_ + 2CO_2_, *ΔH*_r_^0^_298_ = −552.0 kJ mol^−1^) is an exothermic reaction. Owing to the presence of O_2_, it is challenging to achieve a high selectivity of H_2_ since the oxidation of ethanol toward CO_2_ and H_2_O is more energetically favored (CH_3_CH_2_OH(g) + O_2_(g) → 3H_2_O(g) + 2CO_2_(g), *ΔH*_r_^0^_298_ = −1277.4 kJ mol^−1^).^[^ ^279^ ^]^ What is more, from the viewpoint of molecular structure, in contrast with methanol, ethanol illustrated a complex chemical bond network. Photoreforming ethanol for selective H_2_ production is thus more challenging. As the most studied semiconductor, TiO_2_ was also used for photocatalytic hydrogen evolution from bioethanol. It was found that the crystal phase of TiO_2_ played a vital role in the photocatalytic reforming of ethanol toward hydrogen.^[^ ^280^ ^]^ By modifying with Au nanoparticles as cocatalyst, TiO_2_ anatase delivers a hydrogen production rate two orders of magnitude higher than obtained on TiO_2_ rutile. The varied hydrogen production rates can be simply linked to the difference in electron‐hole recombination rates over the two polymorphs of TiO_2_. What is more, platinum oxide (PtO*_x_*) was also immobilized on TiO_2_ for photocatalytic hydrogen production from ethanol aqueous solution.^[^ ^281^ ^]^ The hydrogen production rate increases with the increasing PtO*_x_* loading. Nevertheless, overloading PtO*_x_* was detrimental to the activity because the excessively loaded PtO*_x_* would decrease the light absorption of the photocatalyst via scattering the incident light. Meanwhile, PtO*_x_* can serve as the recombination centers, thus restricting the surface chemical reactions. The H_2_ production rate reached a peak value of 5.3 mmol g_cat_^−1 ^h^−1^ with a quantum efficiency (QE) of 4.6% at the optimal PtO_x_ loading of 0.29 wt%. Additionally, a direct Z‐scheme of CoTiO_3_/TiO_2_ was assembled by dispersing CoTiO_3_ with a narrow bandgap onto TiO_2_ with a large bandgap for photocatalytic H_2_ release from ethanol.^[^ ^282^ ^]^ The overall performance of CoTiO_3_/TiO_2_ obviously outperformed those of TiO_2_, CoTiO_3_, and Co‐doped TiO_2_. The CoTiO_3_/TiO_2_ (1%) exhibited the highest H_2_ production rate of 3.7 mmol h^−1^ g^−1^, which is twofold and 12‐fold higher than TiO_2_ and CoTiO_3_, respectively. Such an excellent catalytic activity of CoTiO_3_/TiO_2_ primarily originated from the type II heterostructure, which enabled efficient charge carrier separation (**Figure** **16**a). A step‐scheme (S‐scheme) 2D–2D heterojunction on thin TiO_2_ nanosheets with a few‐layered MoO_3_ was explored for using a ball milling method.^[^ ^283^ ^]^ As studied by in situ XPS and photoluminescence (PL) measurements, such 2D‐2D heterojunction was favorable for the separation of photo‐generated electrons and holes and for the desorption of H_2_ molecules, thus leading to a threefold increase in the H_2_ production rate of 0.06 µmol h^−1^ from ethanol‐water solution compared with TiO_2_ (0.02 µmol h^−1^). García‐Munoz et al. explored the feasibility of the alveolar open‐cell β‐SiC foam as the photocatalyst support for solar H_2_ generation from ethanol‐water mixtures (Figure 16b).^[^ ^284^ ^]^ The metal/TiO_2‐_coated β‐SiC foams i.e., Ru/TiO_2_/SiC and Pt/TiO_2_/SiC are highly active for hydrogen production from ethanol and water in a gas‐phase reactor coupled to a compound parabolic collector. Irradiated by natural sunlight, Pt/TiO_2_/SiC demonstrated a 14% UV‐to‐hydrogen (equivalent to 0.49% solar‐to‐hydrogen) conversion efficiency. As reported by the same research group, iron‐grafted mesoporous Pt/TiO_2_ photocatalysts were prepared for H_2_ production via a preferential ethanol dehydrogenation reaction pathway.^[^ ^285^ ^]^ XPS and time‐resolved fluorescence measurements showed that a surface heterojunction between Fe_2_O_3_ and TiO_2_ is favorable for the separation of photogenerated electrons and holes. Silica SBA‐15 is one of the most widely used mesoporous supports for synthesizing efficient catalysts because of its rich mesoporous structure and high surface area. Torres‐Garcia et al. reported the immobilization of TiO_2_ nanocrystals with different contents on SBA‐15 for photocatalytic H_2_ production from ethanol.^[^ ^286^ ^]^ The hydrogen production rates of all xTiO_2_/SBA‐15 are much higher than that of bare TiO_2_. The mesoporous SBA‐15 facilitates the dispersion of active sites, thus promoting hydrogen evolution. The addition of Pt greatly boosts the hydrogen generation rate, which is 310‐fold that of the Pt‐free sample.

**Table 5:** Representative examples of photocatalytic H_2_ production from ethanol.

Photocatalysts	Light source	H_2_ evolution rate	Apparent quantum yield (AQY)	Lifetime	Reference
PtO_x_(0.29)/TiO_2_	380 nm LED	5.3 mmol h^−1^ g^−1^	–	4.6%	[281]
CoTiO_3_/TiO_2_ (1%)	365 ± 5 nm UV LEDs, 79.1 mW cm^−2^	3.7 mmol h^−1^ g^−1^	1.1% (365 nm)	–	[282]
0.5Pt‐20%TiO_2_/SBA‐15	6 W fluorescent lamps	895.9 µmol g^−1^	–	15 h	[286]
1.09 wt% AgNPs/CdS	300 W xenon‐lamp	1720.9 µmol g^−1^ h^−1^	–	18 h	[287]
Ni‐Ag/CNx	100 mW/cm^2^	354 µmol g^−1^ h^−1^	0.15% (410 nm)	–	[288]
Ni/NCN‐CN	5 mW/cm^2^	2.32 mmol h^−1^ g^−1^	–	24 h	[289]
Ni_0.04_Cu	574 mW/cm^2^, 210 °C	176.6 mmol g^−1^ h^−1^	3.8%^a)^	–	[290]
Pt‐Ag/Ag_3_PO_4_‐WO_3_ (10 wt%) oxide nanocatalyst	300 W Xe lamp, 125 mW/cm^2^,85 °C	about 5 mmol g^−1^ h^−1^	–	5 cycles	[291]
Ni SA/CeO_2_	300 W/cm^2^, 462 °C	519 mmol h^−1^ g^−1^	16.7% ^a)^	60 h	[292]
Multi‐Au@SiO_2_/TiO_2_	300 W Xe lamp, 100 mW/cm^2^	6313 µmol h^−1^ g^−1^	–	30 h	[296]
Pt/UCN/Nb_2_O_5_‐(2)	100 mW cm^−2^	632 µmol h^−1^ g^−1^	2.18% (400 nm)	five cycles	[299]

^a)^ solar‐to‐fuel (STF) efficiency

Compared with the wide bandgap (3.2 eV) of TiO_2_, the narrow bandgap of CdS (2.4 eV) enabled visible light absorption with wavelength up to 520 nm. CdS semiconductor has been widely studied in various photocatalytic systems. Nevertheless, its overall performance is restricted by the facile recombination of photogenerated electron–hole pairs as well as the severe photocorrosion (CdS + 2h^+^ → Cd^2+^ + S↓). To improve the catalytic performance of CdS, Zhang et al. prepared CdS nanofibers as semiconductors using a microwave‐assisted method, followed by photodepositing silver nanoparticles (AgNPs) for photocatalytic ethanol dehydrogenation.^[^ ^287^ ^]^ Owing to the surface plasmon resonance effect of AgNPs and the generation of Mott‐Schottky heterojunction between CdS and AgNPs, the prepared 1.09 wt% AgNPs/CdS exhibited a wide visible light absorption range, strong electron generation capacity, and excellent electron transfer characteristics. These characteristics endowed a measurable H_2_ evolution rate of 1720.9 µmol h^−1^ g^−1^, which is over 40 times that of pure CdS. After running for 18 h, there was no significant decrease in H_2_ evolution rate.

Carbon nitride (g‐C_3_N_4_), as a metal‐free semiconductor, has been extensively explored for H_2_ production from photoreforming ethanol, due to its good stability, low cost, and visible light response. Modified by Ni‐Ag nanoparticles, the H_2_ evolution rate for Ni‐Ag/CN*_x_* reached 354 µmol g^−1^ s^−1^, which is 4.3 times that of Ni/CN*_x_* (82 µmol g^−1^ s^−1^).^[^ ^288^ ^]^ Herein, Ag can not only disperse and stabilize Ni nanoparticles but also provide catalytic sites to suppress the accumulation of ethanol decomposition products (acetate species), thus leading to an increased H_2_ activity. The modification of carbon nitride with functional groups also presents a viable means for improving the photocatalytic performance. As studied by Gunawan, a cooperative modification of an in situ photodeposited Ni cocatalyst on carbon nitride through cyanamide functionalization and interfacial charge‐induced activation, enables carbon nitride to effectively utilize photogenerated holes for oxidizing ethanol to acetaldehyde, which greatly promotes electron transfer to the Ni cocatalyst for H_2_ generation.^[^ ^289^ ^]^ Meanwhile, the excess Ni^2+^ ions remaining in the solution facilitate both photogenerated electrons utilization and ethanol adsorption. The synergism thus resulted in a remarkable H_2_ production rate of 2.32 mmol h^−1^ g^−1^ coupled with an acetaldehyde production rate of 2.54 mmol h^−1^ g^−1^.

Compared with traditional photocatalysts, plasmonic metals exhibit a relatively broad light absorption range and have attracted increasing attention recently. By virtue of the plasmonic effect of Ni–Cu bimetals, solar‐driven H_2_ generation from ethanol was realized by Ye and co‐workers.^[^ ^290^ ^]^ The optimal Ni_0.04_Cu photocatalyst showed an impressive H_2_ production rate of 176.6 mmol g_cat_^−1^ h^−1^ with a solar‐to‐fuel conversion efficiency of 3.81% under simulated 5.7 suns irradiation without other energy inputs. The mechanism investigations revealed that the synergism of photocatalysis and thermal catalysis was responsible for high activity. The hot electrons produced from Cu nanoparticles were migrated to Ni atoms, thus facilitating the separation of hot carriers and activating the reaction substrates. As a representative endeavor, the plasmonic Pt–Ag metal nanoparticles were decorated on the surface of Ag_3_PO_4_‐WO_3_ for photothermal reforming of ethanol to H_2_.^[^ ^291^ ^]^ It was found that the hydrogen production rate increases with increasing temperature from 25 to 85 °C, which may be attributed to the heat‐promoting product desorption. The H_2_ production rate of Pt–Ag/Ag_3_PO_4_‐WO_3_ (10 wt%) reached 5 mmol g_cat_^−1^ h^−1^ under 300 W Xe lamp irradiation with the light intensity of 125 mW cm^−2^ at 85 °C. The large surface area (16.8 m^2^ g^−1^) and low bandgap of Ag_3_PO_4_–WO_3_, together with the lowered e^−^/h^+^ recombination as a result of plasmonic Pt–Ag metal, primarily contributed to the enhanced H_2_ production. Yuan et al. reported solar‐driven H_2_ evolution by bioethanol steam reforming (CH_3_CH_2_OH + 3H_2_O → 2CO_2_ + 6H_2_) using a Ni SA/CeO_2_–Ti_2_O_3_/Cu hybrid (Figure 16d). The as‐prepared Ti_2_O_3_/Cu performed as a photo‐thermal device under solar irradiation, and Ni SA/CeO_2_ as a catalyst does not only promote the reaction by lowering the energy barrier of ethanol and H_2_O decomposition but also resists coking and sintering (Figure 16e,f).^[^ ^292^ ^]^ Specifically, the Ti_2_O_3_/Cu photothermal device makes the reaction temperature rise from 115 to 462 °C under 3 suns irradiation, and an impressive H_2_ production rate of 519 mmol g_cat_^−1^ h^−1^ was obtained with 6.7% solar‐to‐fuel efficiency. Recently, we proposed an air‐promoted strategy for photocatalytic hydrogen production from bio‐derived ethanol over a core/shell Cr_2_O_3_@GaN nanoarchitecture.^[^ ^293^ ^]^ O_2_, as a promoter rather than a quencher, was converted into active oxygen species, which facilitated the C–C cleavage of the key C_2_ intermediate. It thus significantly reduced the reaction energy barrier of hydrogen generation and then favored removing the carbon residual with inhibited overoxidation. Thus, an impressive H_2_ production rate of 76.9mol g_cat_^−1^ h^−1^ was obtained with a record‐high TON of 266,943,000 mole H_2_ mole Cr_2_O_3_^−1^ over 180 h of long‐term operation.

**Figure 17:**
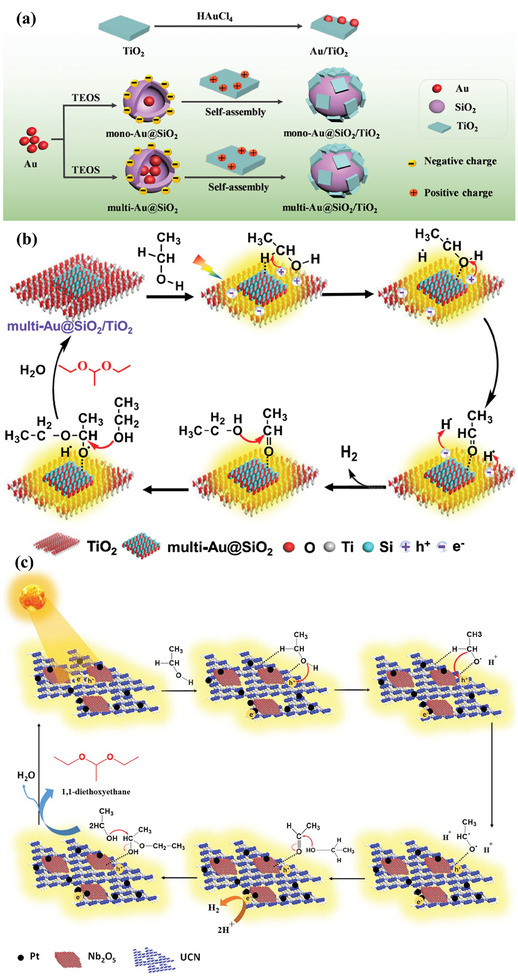
a) Synthesis routes of Au/TiO_2_, mono‐Au@SiO_2_/TiO_2_, and multi‐Au@SiO_2_/TiO_2_, and b) Mechanism of photoreforming ethanol into DEE and H_2_ over Multi‐Au@SiO_2_/TiO_2_. Reproduced with permission.^[^ ^296^ ^]^ Copyright 2020, American Chemical Society. c) Mechanistic illustration of photocatalytic co‐production of DEE and H_2_ over the Pt/UCN/Nb_2_O_5_‐(2) photocatalyst. Reproduced with permission.^[^ ^299^ ^]^ Copyright 2022, The Royal Society of Chemistry.

Photocatalytic co‐production of value‐added chemicals with H_2_ from renewable bioethanol is an extremely appealing approach to improve the economic competitiveness of bioethanol reforming. Besides acetaldehyde, 1,1‐dimethoxyethane (DEE) is also an important compound from ethanol reforming, which is widely used in various applications such as pharmaceuticals and fragrance. Meanwhile, DEE can be utilized as an excellent fuel additive, which is of benefit for improving ignition performance and reducing nitrogen oxide emissions. Usually, the conversion of ethanol to DEE requires a two‐step process: 1) oxidizing ethanol to acetaldehyde; 2) conversion of acetaldehyde to DEE via the acid‐catalyzed acetaldehyde. Various photocatalysts, such as Pt/TiO_2_,^[^ ^294^ ^]^ PdPSA‐CdS,^[^ ^295^ ^]^ Au@SiO_2_/TiO_2_,^[^ ^296^ ^]^ Au/TiO_2_,^[^ ^297^ ^]^ and CdS/Ni‐MoS_2_,^[^ ^298^ ^]^ have been developed for photocatalytic co‐production of DEE and H_2_ from ethanol. Among a range of discoveries, atomically dispersing Pd‐P_3_ species on CdS nanorods (PdPSa‐CdS) showed outstanding performance for photoreforming ethanol into DEE and H_2_.^[^ ^295^ ^]^ In this discovery, the partially reduced Pd centers in PdPSA–CdS exhibited unoccupied hybrid states of Pd 4d and P 3p, which facilitated electron transfer from ethanol to PdPSA, thus promoting C–H and O–H activation of ethanol (Figure 16c). Therefore, the as‐prepared PdPSA‐CdS demonstrated a marked DEE production rate of 35.1 mmol g^−1^ h^−1^ and ≈100% selectivity. What is more, multi‐Au@SiO_2_/TiO_2_ were fabricated through electrostatic self‐assembly, where SiO_2_ and Au particles serve as shell and core, respectively (**Figure** **17**a).^[^ ^296^ ^]^ In the hybrid of multi‐Au@SiO_2_/TiO_2_ SiO_2_ and TiO_2_ worked in synergy for selective activation of C–H and O–H bonds of ethanol, respectively, leading to the generation of CH_3_CH^•^(OH) or CH_3_CH_2_O^•^ radicals, respectively (Figure 17b). Meanwhile, the introduction of SiO_2_ enhances the acidity of the catalyst surface, thus favoring the cleavage of the αC–H bonds of ethanol and the adsorption of the intermediate, i.e., acetaldehyde. As a result, the multi‐Au@SiO_2_/TiO_2_ exhibited a DEE production rate of 6232 µmol g^−1^ h^−1^ with >99% selectivity and a H_2_ production rate of 6313 µmol g^−1^ h^−1^. Chauhan et al. demonstrated the integration of Nb_2_O_5_ with Lewis acidity and urea‐derived carbon nitride (UCN) to construct a heterostructure for photocatalytic dehydrogenation of ethanol into DEE and H_2_ using Pt as a co‐catalyst (Figure 17c).^[^ ^299^ ^]^ The heterostructure facilitates the separation and transportation of photogenerated electron–hole pairs. Ethanol was first oxidized into acetaldehyde and 2H^+^ by the holes localized in UCN. In the meantime, H^+^ was reduced H_2_ by Pt‐mediated electron. Subsequently, the generated acetaldehyde is adsorbed and activated by active sites (e.g., –OH, Nb^5+^) of Nb_2_O_5_ to produce DEE. The highest production rates of DEE and H_2_ over the Pt/UCN/Nb_2_O_5_‐(2) photocatalyst are 594 and 632 µmol g^−1^ h^−1^, respectively.

### 7.3 Glycerol for Photocatalytic H_2_ Generation

Glycerol, as a typical by‐product of the biodiesel industry, possesses a large annual output of ≈3 million tons in 2020.^[^ ^300^ ^]^ Photocatalytic reforming of glycerin (C_3_H_8_O_3_ + 3H_2_O → 7H_2_ + 3CO_2_, ΔG = 5.3 kJ mol^−1^) also presents a sustainable route for hydrogen production because of its availability, renewability, and low price. It is worth noting that glycerol valorization is also conducive to improving the economic competitiveness of the biodiesel industry. Thus far, various photocatalytic systems have been developed for efficient and long‐lasting H_2_ generation from glycerol (**Table** **6**). Among the reported systems for photoreforming of glycerol into H_2_, Pt/TiO_2_, a typical photocatalyst, showed that the crystalline phases of TiO_2_ had a significant impact on H_2_ production and rutile TiO_2_ is more conducive to H_2_ generation than anatase TiO_2_.^[^ ^301^ ^]^ What is more, the size and distribution of Pt nanoparticles also affected the reaction. Uniformly dispersed Pt NPs with small sizes are beneficial for the achievement of high activity. As an earth‐abundant alternative, NiO was coupled with TiO_2_ for producing H_2_ production from photocatalytic reforming of glycerol.^[^ ^194^ ^]^ It is found that the E*_g_* value of NiO/TiO_2_ decreases as the NiO loading increases, which can accelerate the rate of hydrogen production from glycerol. Nevertheless, excessive nickel loading reduces the hydrogen production rate owing to the reduced surface area of the catalyst. Isotopic experiments suggested that both water and glycerol contributed to the formation of H_2_ generation over NiO/TiO_2_, whereas glycerin was the main source of H_2_. What is more, the activity of NiO was higher than that of other transition metal oxides (e.g., CoO and CuO). USY‐TiO_2_‐metal (Pt, Au, and Ag) composites were also explored for improving the performance of glycerol photoreforming by incorporating TiO_2_ into ordered porous USY zeolite to avoid crystallite size growth and aggregation of TiO_2_ nanoparticles.^[^ ^302^ ^]^ The influence of co‐catalyst on the activity was also studied, following the descending order of Pt > Au > Ag. It is closely related to their work functions. It is worth mentioning that the activity of USY‐TiO_2_‐Pt is more than four times that of unsupported TiO_2,_ which may be attributed to the unique properties of USY and the interaction between USY and TiO_2_. To further optimize the performance of TiO_2_, anatase titania was loaded on activated mesoporous carbon (MCH), aiming at achieving a high specific surface area. Platinum was subsequently photodeposited on anatase titania.^[^ ^303^ ^]^ This method renders uniform dispersion of anatase titania on mesoporous carbon and good deposition of platinum on anatase titania. The obtained Pt/TiO_2_‐MCH photocatalyst with a high specific surface area of 157 m^2^ g^−1^ exhibited a remarkable H_2_ yield of 863 mmol H_2_ g^−1^_cat_ from glycerol photoreforming after 12 h of reaction, which was much higher than those of Pt/TiO_2_ (274 mmol H_2_ g^−1^_cat_) and pure Evonik P25 (2 mmol H_2_ g^−1^_cat_). Meanwhile, the Pt/TiO_2_‐MCH photocatalyst performed better resistance to deactivation compared with Pt/TiO_2_ and pure Evonik P25, because mesoporous carbon inhibited the adsorption and poisoning of TiO_2_ by reaction intermediates. The depositing method for loading Pt onto TiO_2_ was also investigated.^[^ ^304^ ^]^ It was found that the Pt/TiO_2_‐m_SOMC prepared using the surface organometallic chemistry (SOMC) method exhibited higher hydrogen production activity (4.93 mmol g^−1^ h^−1^) than that synthesized using impregnation (3.06 mmol g^−1^ h^−1^) and precipitation methods (3.68 mmol g^−1^ h^−1^). This is mainly attributed to that SOMC enables highly uniform dispersion of Pt nanoparticles with a small size of 1 nm which is highly efficient for mediating chares and chemical species behavior. Owing to an appropriate band structure, i.e., suitable bandgap (2.7–2.9 eV) and redox properties, CeO_2_ is also an ideal building block of photocatalytic architecture for glycerol reforming.^[^ ^305^ ^]^ A synergetic photocatalytic hybrid of CeO_2_‐Au‐rGO was developed for photoreforming of glycerol aqueous solution into H_2_. In this hybrid, the introduced rGO allowed for expanding the light absorption range of CeO_2_ and promoting the good dispersion of Au NPs while Au as a co‐catalyst facilitated H_2_ evolution via surface plasmon resonance effect. The combined effects leaded to H_2_ production at an appreciable rate of 270 µmol g^−1^ h^−1^ under visible light irradiation.

**Table 6:** Representative examples of photocatalytic H_2_ production from glycerol.

Photocatalysts		Light source	H_2_ evolution rate	Lifetime	Reference
Pt/TiO_2_‐m_SOMC	1 M glycerol aqueous solution	300 W Xe lamp, 100 mW cm^−2^	4.93 mmol g^−1^ h^−1^	–	[304]
CeO_2_‐rGO‐Au	10% (v/v) glycerol aqueous solution	150 W Xe lamp	270 µmol g^−1^ h^−1^	five subsequent runs	[305]
4%BiOCl@ZIS/0.0625 wt% Pt	5% (v/v) glycerol: water mixture	1000 W Xenon lamp, >420 nm	674 µmol g^−1^ h^−1^	8 h	[307]
CdO‐QDs/CdS	10 mg glycerol, 0.95 mL CH_3_CN and 0.05 mL H_2_O	LED light 455 nm	12.5 µmol g^−1^ h^−1^	–	[308]
CdS‐Cl	10 mg glycerol, 0.95 mL CH_3_CN and 0.05 mL H_2_O	LED light 455 nm	0.36 mmol g^−1^ h^−1^	–	[309]
ZCNS	10 vol% glycerol aqueous solution	UV‐visible irradiation	9.3 mmol g^−1^ h^−1^	five cycles	[310]
Pt@UiO‐66(Zr)‐NH_2_	4 vol% glycerol aqueous solution	Hg‐Xe lamp‐150W‐L8253	0.77 mmol g^−1^ h^−1^	70 h	[312]
2D Au/TiO_2_	10 vol% glycerol aqueous solution, 65 °C	300 W Xe lamp, 320–780 nm	4.2 mmol g^−1^ h^−1^	6 h	[315]
1% Ag/TiO_2_	10 vol% glycerol aqueous solution, 337 K	300 W Xe lamp, 320–780 nm, 350 mW cm^−2^	7956 µmol g^−1^ h^−1^	–	[316]

**Figure 18:**
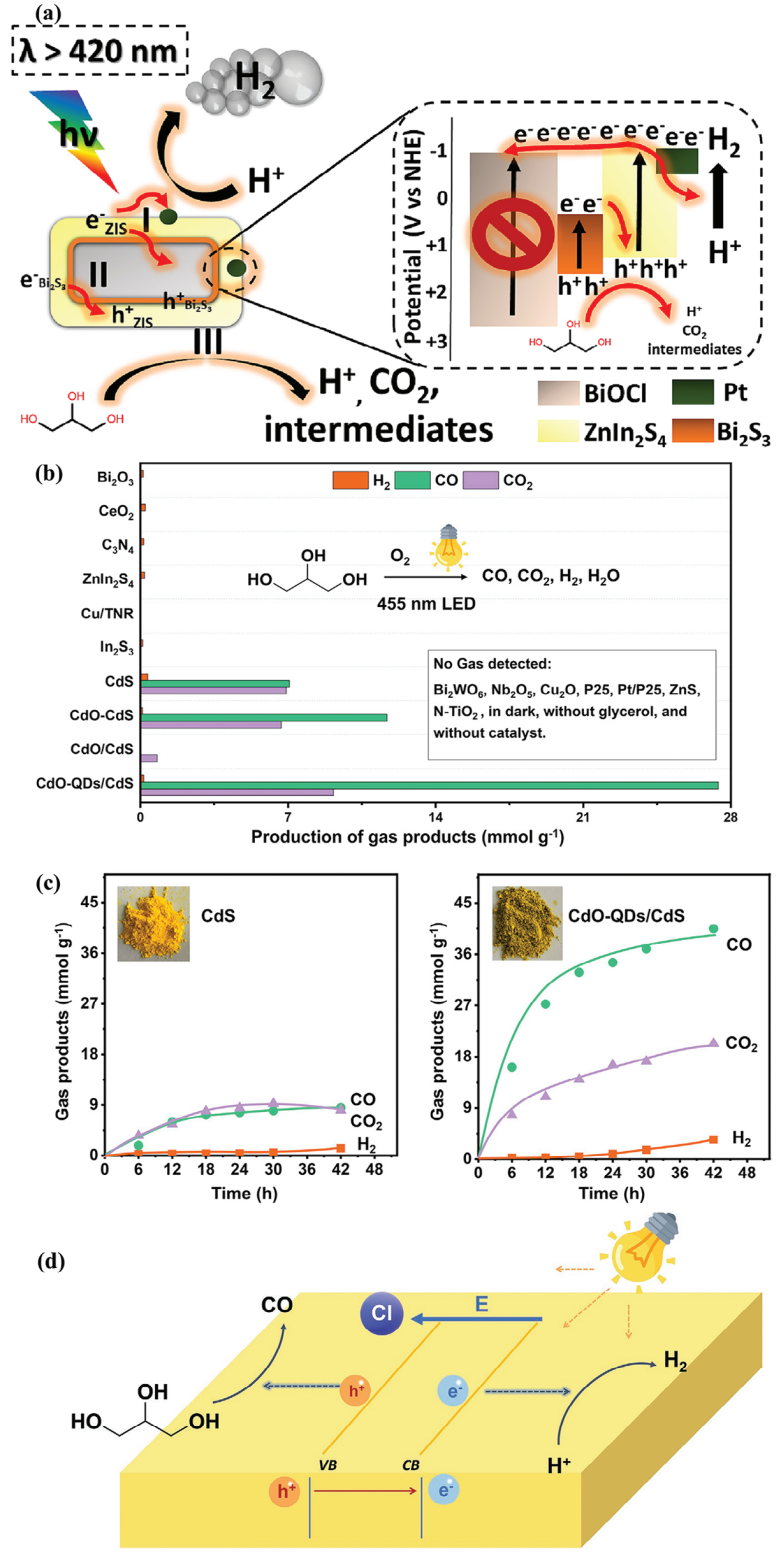
a) The overall mechanism of PHE over BiOCl@ZIS/Pt composite under visible light irradiation. Reproduced with permission.^[^ ^301^ ^]^ Copyright 2023, Elsevier Inc. b) Catalyst screening for photocatalytic glycerol reforming, and c) Comparison of catalytic activity between CdS and CdO‐QDs/CdS catalysts. Reproduced with permission.^[^ ^307^ ^]^ Copyright 2022, Cell Press. d) Mechanism of the photocatalytic reforming of glycerol on CdS‐Cl. Reproduced with permission.^[^ ^309^ ^]^ Copyright 2023, Wiley‐VCH.

**Figure 19:**
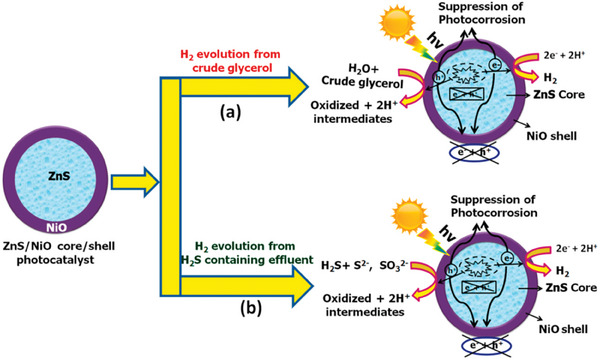
Proposed reaction mechanism for a) photocatalytic H_2_ generation from glycerol aqueous solution and b) H_2_ evolution from H_2_S over the ZCNS core‐shell nanocomposite. Reproduced with permission.^[^ ^310^ ^]^ Copyright 2021, Elsevier Inc.

From the optoelectronic and chemical points of view, metal sulfides often show a narrower bandgap for more effective sunlight harvesting and surface redox reactions compared with metal oxide, because of their higher VB position occupied by the S‐3p orbital than that contributed from O‐2p orbital.^[^ ^306^ ^]^ Metal sulfides have attracted tremendous research interest for photocatalysis as a result of their unique occupied/unoccupied orbitals, as well as catalytic properties. Thus far, great efforts have been devoted to developing metal sulfide‐based semiconductors for photocatalytic reforming of glycerol toward H_2._ As a typical attempt, ZnIn_2_S_4_ (ZIS) nanosheets were coupled with BiOCl microplates for photoreforming of glycerol into H_2_.^[^ ^307^ ^]^ The 4%BiOCl@ZIS/0.0625 wt% Pt photocatalyst, containing 0.0625 wt% Pt cocatalyst and 4% wt% BiOCl microplates, showed an optimal hydrogen production rate of 674 µmol g^−1^ h^−1^, which was ≈1.75‐fold that of ZIS/0.0625 wt% Pt (384 µmol g^−1^ h^−1^) under the same condition. The mechanism study revealed that the in situ formation of low bandgap Bi_2_S_3_ semiconductor between ZIS and BiOCl was the key to improving the catalytic performance by broadening light absorption region and enhancing photo‐generated e^−^/h^+^ separation (**Figure** **18**a). Recently, plasma‐assisted construction of CdO quantum dots (QDs)/CdS photocatalyst was reported for photocatalytic conversion of glycerol in the presence of O_2_.^[^ ^308^ ^]^ The intimate heterojunction between CdO‐QDs and CdS and the quantum size of CdO‐QDs greatly facilitated the separation and migration of photogenerated electrons, promoting the photocatalytic syngas generation from glycerol. The H_2_, CO, and CO_2_ generation rates were 0.15, 27.4, and 9.2 mmol g_cat_^−1^ after 12 h reaction (Figure 18b,c). It is noted that Bio‐CO evolution rather than hydrogen production is the focus of this study. Zhang et al. proposed a strategy of decorating chlorine on the CdS surface by impregnation to reduce the recombination of photo‐generated electrons and holes.^[^ ^309^ ^]^ The study found that chlorine (Cl), distributed on the surface of CdS (CdS‐Cl), was able to enhance the internal electric field and improve charge separation and migration to the surface (Figure 18d). Meanwhile, the CdS‐Cl composite enabled inhibiting hole migration and extending photocarrier lifetime. These characteristics endow a CO production rate of 0.48 mmol g^−1^ h^−1^ and a H_2_ production rate of 0.36 mmol g^−1^ h^−1^ from glycerol photoreforming, which were 13‐ and 11‐fold higher than that of pristine CdS, respectively. ZnS is another class of metal sulfide semiconductor, possessing high mobility of photogenerated electrons to the surface, which is deemed as the potential semiconductor for photocatalytic H_2_ production. The ZnS/NiO nanocomposites with core‐shell hierarchal nanostructures (ZCNS) were developed by wrapping ZnS with NiO, where ZnS was core with a size of ≈158 nm and NiO was a shell with a thickness of ≈9.3 nm.^[^ ^310^ ^]^ The NiO not only serves as a shell to protect the core ZnS from photo‐corrosion, but also as a co‐catalyst to effectively separate photo‐generated electrons (**Figure** **19**). The H_2_ production rate reached 9.3 mmol g^−1^ h^−1^ from 10 vol% glycerol aqueous solution, which was 1.47 and 4.4 times higher than those of ZnS and NiO, respectively.

Metal–organic frameworks (MOFs), as a new class of porous crystalline materials, are composed of metal nodes and organic ligands linked by coordination bonds. In view of their unique characteristics of uniform pore structures with high surface areas, chemical tunability, structural designability, high compatibility with other materials, and easy modification with functional groups, MOFs have also received considerable research interest in photocatalytic H_2_ production from glycerol.^[^ ^311^ ^]^ For example, the Zr‐MOFs including UiO‐66(Zr)‐X (X: H, NO_2_, NH_2_) and MIL‐125(Ti)‐NH_2_ were prepared for photocatalytic H_2_ production from glycerol aqueous solutions.^[^ ^312^ ^]^ The descending order of H_2_ production activity is UiO‐66(Zr)‐NH_2_ > UiO‐66(Zr)‐ NO_2_ > MIL‐125(Ti)‐NH_2_ > UiO‐66(Zr). The order of activity is mainly attributed to the energy band difference of the photocatalysts, and UiO‐66(Zr)‐NH_2_ has a suitable bandgap (2.81 eV) that can respond to visible light and facilitate reduction reactions. When Pt nanoparticles as co‐catalyst were deposited within the UiO‐66(Zr)‐NH_2_ network, the catalytic activity of UiO‐66(Zr)‐NH_2_ was greatly improved from 1.7 to 2.3 mmol g^−1^ within 3 h.

**Figure 20:**
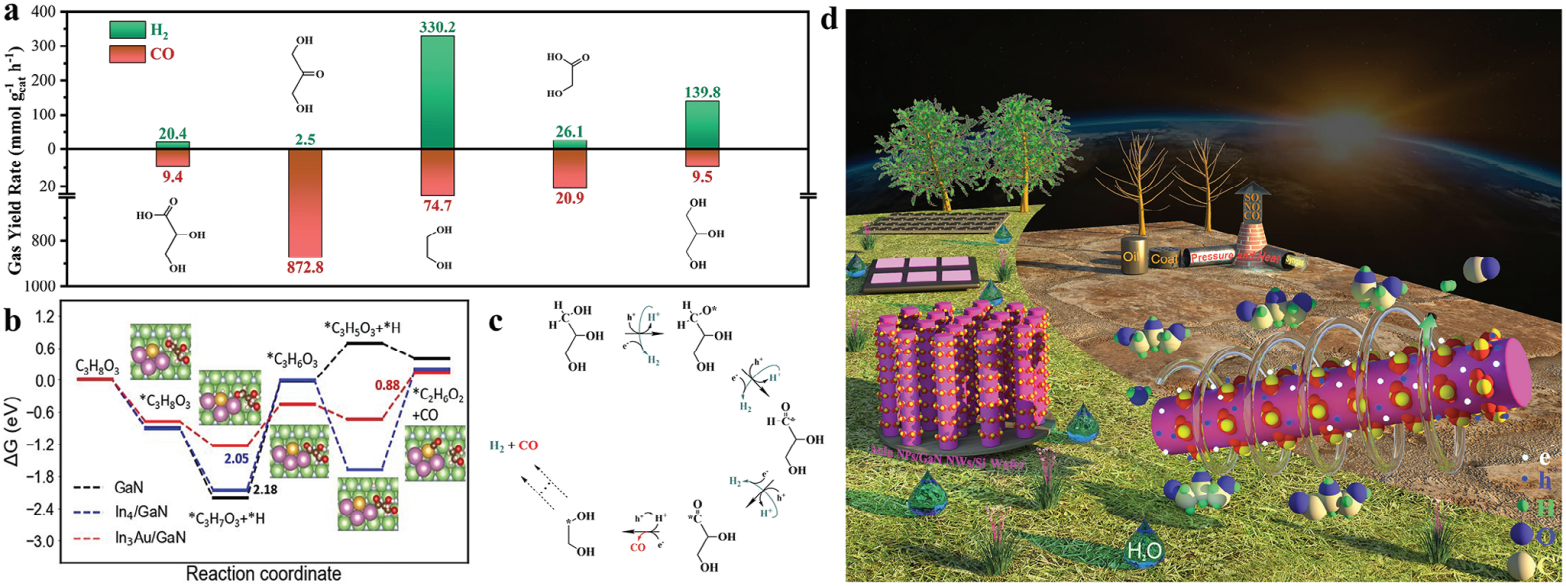
Green syngas production from glycerol, water, and sunlight over AuIn NPs/GaN NWs/Si. a) The syngas activities of several possible C_2_ and C_3_ intermediates. b) Calculated free‐energy diagram for glycerol reforming. c) Photocatalytic glycerol reforming. d) Schematic illustration of syngas production from glycerol and water. Reproduced with permission.^[^ ^314^ ^]^ Copyright 2023, The Royal Society of Chemistry.

Owing to its excellent optical, electronic, and catalytic characteristics, one‐dimensional gallium nitride nanowires vertically aligned onto silicon substrate are appearing as a next‐generation semiconductor platform for photocatalysis.^[^ ^313^ ^]^ Most recently, dual gold‐indium (AuIn) nanoparticles (NPs)‐decorated GaN nanowires/Si (AuIn NPs/GaN NWs/Si) were employed for photocatalytic syngas production from glycerol (**Figure** **20**).^[^ ^314^ ^]^ The synergism of AuIn cocatalysts and GaN nanowires promotes the dehydrogenation of the edge ─CH_2_OH group in the glycerol skeleton. Moreover, the reaction energy barrier is greatly reduced by the cleavage of the inert C–C bond to generate an ethylene glycol‐based intermediate. The syngas production rate of 149.3 mmol g^−1^ h^−1^ with a tailored H_2_/CO ratio from 19.3 to 0.9 was realized under focused simulated sunlight conditions with 4 W cm^−2^ light intensity. Such an endeavor opens a new way for solar‐driven biomass valorization with the use of the emerging Ga(X)N NWs/Si semiconducting architecture.

**Figure 21:**
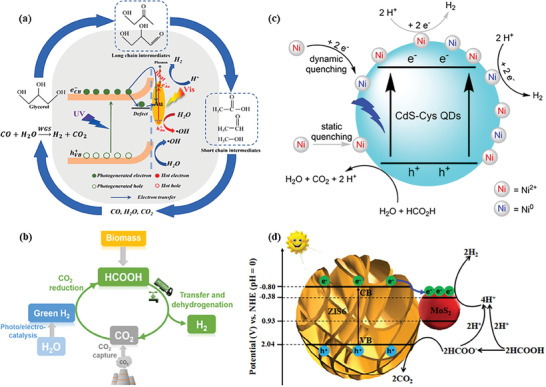
a) Proposed mechanism of photothermal reforming of glycerol over 2D Au/TiO_2_. Reproduced with permission.^[^ ^315^ ^]^ Copyright 2021, Elsevier Inc. b) Carbon cycle for utilization of formic acid as a LOHC. Reproduced with permission.^[^ ^319^ ^]^ Copyright 2023, Cell Press. c) Proposed mechanism for hydrogen production from formic acid photoreforming over Ni/CdS QDs. Reproduced with permission.^[^ ^333^ ^]^ Copyright 2023, Wiley‐VCH. d) Proposed mechanism for photocatalytic H_2_ production from formic acid over 0.5% MoS_2_/Zn_3_In_2_S_6_. Reproduced with permission.^[^ ^335^ ^]^ Copyright 2021, Elsevier Inc.

To improve the overall efficiency, photothermal catalysis was employed to convert glycerin to hydrogen using plasmonic Au‐loaded TiO_2_ nanoflakes (**Figure** **21**a).^[^ ^315^ ^]^ The immobilization of plasmonic Au nanoparticles had no obvious change in the bandgap of TiO_2_. Notably, Au nanoparticles affected the catalytic activity of TiO_2_ via the surface plasmonic resonance effect. In particular, Au nanoparticles could effectively convert solar energy into heat, which can promote the collision of substrate molecules. They could also accelerate the migration efficiency and decrease the recombination of photogenerated electrons and holes. These outstanding properties led to an impressive hydrogen production rate of 4.2 mmol g^−1^ h^−1^ at 65 °C. The influence of temperature on the Ag/TiO_2_ activity for photothermal reforming of glycerol to hydrogen production was further investigated.^[^ ^316^ ^]^ It was observed that the temperature had a significant influence on hydrogen production and could be regulated by controlling the loading of plasmonic Ag. The plasmonic Ag loading within weight ratios of 1–5% is positively related to the temperature of the Ag/TiO_2_ catalyst. In sharp contrast, the temperature can be lowered when the Ag loading is 9% because thermal diffusion is faster than the photothermal effect at a high Ag loading. The hydrogen evolution rate of the optimal 1% Ag/TiO_2_ was as high as 7956 µmol g^−1^ h^−1^ at 337 K.

### 7.4 Solar‐Driven Formic Acid Reforming for Hydrogen Generation

Formic acid (HCOOH, FA), as the simplest carboxylic acid, is a colorless bulk chemical with a global annual yield of ≈1500 kilotons.^[^ ^317^ ^]^ Of note, as a grand promise, formic acid can be broadly produced from biomass conversion and CO_2_ hydrogenation in the future (Figure 21b).^[^ ^318^, ^319^ ^]^ Due to its merits of renewability, high volumetric capacity (53.4 g H_2_ L^−1^), high hydrogen content (4.4 wt%), low toxicity, low flammability, and liquid phase at ambient conditions,^[^ ^320^ ^]^ formic acid has also been widely utilized as an ideal LOHC for on‐site hydrogen production. It thus offers an appropriate solution for addressing the critical issues associated with H_2_ storage and transportation, which is the bottleneck of utilizing H_2_ as a future energy carrier.^[^ ^321^, ^322^ ^]^ (**Table** **7**). Hydrogen production from FA can proceed via two main paths of dehydrogenation toward H_2_ and CO_2_ (HCOOH → H_2_ + CO_2_, Δ*H*° = −14.9 kJ mol^−1^) and dehydration toward CO and H_2_O (HCOOH → H_2_O + CO, Δ*H*° = 26.2 kJ mol^−1^), respectively.^[^ ^323^ ^]^ The desired dehydrogenation (Δ*G* = −32.8 kJ mol^−1^) is thermodynamically favored compared to the dehydration path (Δ*G* = −20.7 kJ mol^−1^). The development of suitable photocatalysts that enable selective hydrogen production via dehydrogenation rather than dehydration of FA is highly desirable.

**Table 7:** Representative examples of photocatalytic H_2_ production from formic acid.

Photocatalysts	Light source	H_2_ evolution rate	AQY	Lifetime	Reference
Cosal‐NH_2_‐MIL‐68@In_2_S_3_	300 W Xe lamp, >420 nm	18746 µmol g^−1^ h^−1^	–	–	[172]
NiCoP@CdS	300 W Xe lamp, λ > 420 nm	354 µmol g^−1^ h^−1^	45.5% at 420 nm	>48 h	[288]
Au@Pd/UiO‐66‐ (Zr85Ti15)	Xe lamp, 320 mW/cm^2^, λ > 420 nm	42000 mL g^−1^ h^−1^	–	–	[329]
UiO‐66(COOH)_2_‐Cu	Xe lamp, 71 mW/cm^2^, >390 nm	5 mmol g^−1^ h^−1^	–	3 days	[332]
Ni/CdS QDs	10 W blue LEDs, 50 °C	282 µmol g^−1^ h^−1^	–	60 h	[333]
CuFe_2_O_4_	300 W Xe lamp, 100 mW cm^−2^	6.6 mmol g^−1^ h^−1^	7.3%	40 h	[334]
0.5 MoS_2_/Zn_3_In_2_S_6_	300 W Xe lamp, λ > 420 nm	74.25µmol h^−1^	2.9% at 400 nm	–	[335]
MIP‐177‐LT	300 W Xe lamp	650 µmol g^−1^ h^−1^	22% in the UV region	–	[338]
CdS/CoP@RGO	LED λ ≥ 420 nm), 11 mW cm^−2^	182 mmol g^−1^ h^−1^	–	>7 day	[340]
CoPSA‐CdS	300 W Xe lamp, 800 mW cm^−2^	34309 mmol g^−1^ h^−1^	–	24 h	[72]
FeP@CdS	300 W Xe lamp, λ > 420 nm	556 µmol h^−1^	54% at 420 nm	>4 day	[342]
CdS/W_2_N_3_	300 W Xe lamp, λ > 420 nm	262 mmol h^−1^	17.6% at 420 nm	>50 h	[343]

Recently, a variety of photocatalysts have been developed for FA dehydrogenation by coupling noble metals such as Pd,^[^ ^324^ ^]^ Au,^[^ ^325^ ^]^ Ag,^[^ ^326^ ^]^ Pt,^[^ ^327^ ^]^ and Ru^[^ ^328^ ^]^ with different semiconductor materials. For instance, the plasmonic Au@Pd nanoparticles supported‐UiO‐66(Zr_85_Ti_15_) bearing ─NH_2_ groups were fabricated for photocatalytic reforming of FA toward H_2_.^[^ ^329^ ^]^ The synergistic effect of the electron‐rich Pd resulting from the surface plasmon resonance (LSPR) effect of Au and NH_2_ groups of UiO‐66 facilitated the cleavage of O─H and C─H of FA, resulting in a high H_2_ production rate of 42 000 mL h^−1^ g^−1^ (Pd) with a turnover frequency (TOF) of 200 h^−1^. Different noble metals including Pt, Pd, Ru, and Au, supported by CdS as the photocatalysts were also employed for the photoreforming of FA aqueous solution into H_2_.^[^ ^330^ ^]^ It was found that the hydrogen production rate declined in the order of Pt/CdS > Pd/CdS > Ru/CdS > Au/CdS, further suggesting that Pt is a state‐of‐the‐art HER cocatalyst. Meanwhile, the critical role of H_2_O in facilitating H_2_ production also was studied by experimental investigation and theoretical calculation. The introduction of H_2_O in the catalytic systems showed a two‐ to fourfold enhancement in H_2_ production, which primarily originated from the remarkable reduction of the activation energy of the reaction. Specifically, OH* (activated OH^−^) and H* (activated H^+^) from H_2_O enabled to facilitate the O–H bond rupture of FA by hydrogen‐bonding interaction and boost H_2_ generation by combining with the H* from FA, respectively. Cheng and coworkers demonstrated that AgPd/N‐ompg‐C_3_N_4_ catalysts were prepared by immobilization of AgPd nanoparticles on N‐deficient ordered mesoporous graphitic carbon nitride (N‐ompg‐C_3_N_4_) for photocatalytic H_2_ evolution from FA.^[^ ^331^ ^]^ The Ag_0.1_Pd_0.9_/N‐ompg‐C_3_N_4_ photocatalyst possessed unique characteristics of large surface area and surface defects, strong interaction of AgPd and N‐ompg‐C_3_N_4_, and efficient charge transfer, thus leading to a superior turnover frequency (TOF) value of 1588.2 h^−1^.

For the sake of practical applications, a number of non‐noble metal cocatalysts have been explored for hydrogen production from FA by photocatalysis. The UiO‐66‐(COOH)_2_‐Cu catalyst, synthesized by metalation of carboxylic‐functionalized MOF (UiO‐66(COOH)_2_) with copper nitrate, demonstrated a H_2_ production rate of 5 mmol g_cat_^−1^ h^−1^ with >99.99% selectivity and stability of three days under visible light.^[^ ^332^ ^]^ The superior photocatalytic performance compared with UiO‐66‐Cu was ascribed to the generation of Cu^0^/Cu_2_O nanoparticles trapped in the UiO‐66 cages during the in‐situ restructuring process. The CdS quantum dots (QDs) is attractive semiconductor because of their unique properties for light harvesting and charge separation. The Ni/CdS QDs photocatalyst were prepared by in‐situ photo deposition of Ni^2+^ cocatalyst on CdS QDs for photocatalytic H_2_ production from formic acid aqueous solution.^[^ ^333^ ^]^ The Ni° cocatalysts, formed in the dynamic quenching as an electron outlet, facilitate charge transfer and improve the CdS QDs stability by significantly decelerating CdS QDs photocorrosion (Figure 21c). This noble‐metal‐free catalytic system offered a H_2_ production rate of 282 µmol g_cat_^−1^ h^−1^ with 99.8% H_2_ selectivity under visible light irradiation at 50 °C. Thanks to its unique characteristics of high charge transfer, redox properties, visible light response, nontoxicity, and low cost, the CuFe_2_O_4_ spinel was employed as a photocatalyst for the photoreforming of formic acid to H_2_.^[^ ^334^ ^]^ When the molar ratio of Cu to Fe is 3:1 and the annealing temperature is 800 °C, the CuFe_2_O_4_ spinel exhibits the optimal activity with a H_2_ release rate of 6.6 mol g_cat_^−1^ h^−1^ and an apparent quantum yield of 7.3%. This activity is mainly ascribed to the uniformly distributed tetrahedral Cu^2+^(OH)_2‐x_ sites on the CuFe_2_O_4_ surface and the in‐situ restructuring phenomenon. During the in situ reconstruction process, tetrahedral Cu^2+^(OH)_2‐x_ sites were restructured in situ into a trinary (Cu^+^/Cu^0^/spinel), resulting in the formation of Z‐scheme heterojunction, which promotes the separation of photogenerated electrons and holes pairs and the reduction of protons to H_2_. Considering its narrow bandgap with strong visible light absorption, accessibility, and outstanding ability, MoS_2_ has emerged as an appropriate alternative to noble metals for hydrogen evolution reactions. The MoS_2_/Zn_3_In_2_S_6_ photocatalysts were synthesized using the one‐pot hydrothermal method, where MoS_2_ was highly dispersed on Zn_3_In_2_S_6_ (ZIS6) microspheres.^[^ ^335^ ^]^ The 0.5% MoS_2_/Zn_3_In_2_S_6_ photocatalyst exhibited the highest hydrogen production rate of 74.25 µmol h^−1^ from formic acid with a quantum efficiency of 2.9% at about 400 nm, which surpassed those of pure Zn_3_In_2_S_6_ and MoS_2_. The MoS_2_ with a low Fermi energy level can effectively trap photo‐generated carriers from Zn_3_In_2_S_6_ to reduce H^+^ to H_2_, facilitating the separation of photogenerated electrons and holes and H_2_ generation from formic acid (Figure 21d). Metal–organic frameworks (MOFs) are attracting tremendous interest in the field of photocatalysis owing to their versatility of ordered porous structures with high surface areas and adjustable compositions^[^ ^336^, ^337^ ^]^ Especially, titanium oxocluster‐based Ti‐MOFs are a very attractive class of materials as photocatalysts due to containing Ti metal ions exhibiting photophysical response and redox photoactivity. In view of the robust 3D microporous structure and remarkable stability against strong acids, MIP‐177‐LT was employed as a photocatalyst for hydrogen production from FA.^[^ ^338^ ^]^ MIP‐177‐LT exhibited 650 µmol g_cat_^−1^ h^−1^ H_2_ release, which is higher than those of MIL‐125(Ti) (133 µmol g_cat_^−1^ h^−1^), UiO‐66(Zr) (180 µmol g_cat_^−1^ h^−1^) and TiO_2_ P25 (170 µmol g_cat_^−1^ h^−1^).

**Figure 22:**
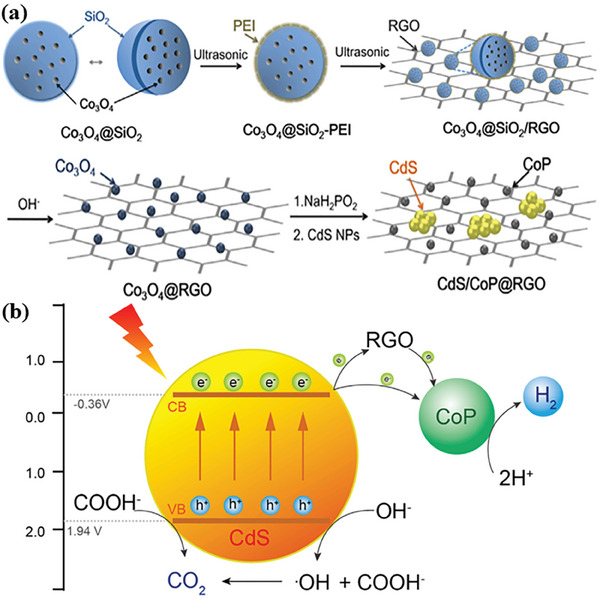
a) Schematic illustration of the CdS/CoP@RGO. b) Proposed mechanism for photocatalytic H_2_ production from formic acid over CdS/CoP@RGO. Reproduced with permission.^[^ ^340^ ^]^ Copyright 2018, Cell Press.

Transition‐metal phosphides, such as CoP, FeP, Ni_2_P, and MoP, have recently emerged as a family of cocatalysts for practical photocatalytic hydrogen release from formic acid.^[^ ^339^ ^]^ The remarkable characteristics of the catalytic property, good stability, and low cost make them promising alternative to noble metals for hydrogen generation. In detail, by integrating ultra‐small CoP nanoparticles with the semiconducting hybrid of CdS/@RGO synthesized by loading CdS nanoparticle on reduced graphene oxide (RGO), a multifunctional CdS/CoP@RGO photocatalyst was designed for hydrogen production from formic acid (**Figure** **22**a).^[^ ^340^ ^]^ It is found that the CoP nanoparticles (CoP NPs) exhibited metal‐like property and superior electrical conductivity through XPS and current‐voltage measurement, which facilitates charge transfer during the photocatalytic process. Strikingly, the as‐prepared CoP even exhibits lower hydrogen desorption energy than state‐of‐the‐art Pt. Owing to the superior catalytic property, the H_2_ production rate of CdS/CoP@RGO reached 182 mmol g^−1^ h^−1^, which was over 30 times higher than that of bare CdS. Meanwhile, the selectivity of H_2_ was as high as >99.5%. The reaction mechanism was investigated by using terephthalate fluorescence probing and electron spin resonance measurements. The hydrogen protons (H^+^) derived from formic acid and H_2_O were reduced to H_2_ on the CoP NPs by photogenerated electrons from CdS. The COOH^−^ anion was oxidated into CO_2_ by  OH radicals on the VB band of CdS (Figure 22b). Similarly, CdS nanorods decorated with atomically dispersed Co‐P_3_ species (CoPSA‐CdS) were also utilized for FA photoreforming toward H_2_.^[^ ^72^ ^]^ Co‐P_3_ species enabled the dissociation of the adsorbed formic acid and activated the C–H bond of formic acid. The CoPSA‐CdS showed an impressive H_2_ evolution rate of 34309 mmol g^−1^ h^−1^, even outperforming state‐of‐the‐art noble metals, i.e., Ru‐, Rh‐, Pd‐, and Pt‐loaded CdS nanorods. The crystalline iron phosphide (FeP) nanoparticles with ≈5–10 nm and NiCoP nanoparticles with ≈5 nm as co‐catalysts were anchored to CdS nanorods as well. Both FeP and NiCoP were capable of effectively separating the electron–hole pairs produced by CdS nanorods, which was highly beneficial for hydrogen generation. The H_2_ evolution rate for NiCoP@CdS reached ≈354 mmol g^−1^ h^−1^, which was even superior to that of Pt@CdS, Au@CdS, Ru@CdS, Pd@CdS, and Ag@CdS.^[^ ^341^ ^]^ What is more, the H_2_ production rate over FeP@CdS was ≈556 µmol h^−1^, which was ≈37 times higher than that of bare CdS.^[^ ^342^ ^]^

W_2_N_3_ has gained significant research interest in photocatalytic H_2_ production owing to its Pt‐like characteristics and unique photo‐electric properties of wide light absorption range, remarkable conductivity, and high carrier density. Wang and coworkers synthesized crystalline W_2_N_3_ nanosheets by a hydrothermal method and high‐temperature calcination in an ammonia atmosphere and subsequently combined them with CdS nanorods to construct type‐II heterojunction through two steps process of grinding and calcination.^[^ ^343^ ^]^ The formed type‐II heterojunction between CdS and W_2_N_3_ is highly favorable for photogenerated charge carriers separation and transfer. In conjunction with the rich active sites and promoted reaction kinetics, the composite CdS/W_2_N_3_ photocatalyst exhibited a distinct production rate of ≈262 mmol h^−1^ for H_2_ and 207 mmol h^−1^ for CO, which were 9.4 and 11.5 times higher than those of bare CdS, respectively. The apparent quantum yields of H_2_ and CO generation were 17.6% and 16.9%, respectively. These results further validate the viability of using cost‐effective alternatives to noble metals for hydrogen production from biomass derivatives.

Based on the detailed discussion above, it is discovered that semiconductor‐based photocatalysis provides a promising strategy for the sustainable production of green hydrogen with the inputs of biomass and sunlight. What is more, since some of the underutilized biomass such as lignin from the pulping industry is well known as an environmental pollutant, photocatalytic reforming also presents an environment‐friendly and economically favorable route for biomass utilization. Considering the rich C and O content with diverse functional groups, rational design of chemical routes for biomass reforming will further improve the economic benefits. It is worth noting that the in situ formation of active H species from photocatalytic biomass reforming and water splitting is an absolutely green reductant, which can initiate important reduction reactions under ambient conditions. It thus holds a grand promise for avoiding the drawbacks of conventional thermocatalytic reduction reactions. Please see below the recent advance in using biomass as a green hydrogen resource for photocatalytic production of value‐added chemicals.

Except for semiconductor structures as discussed in Section 3, the photoreforming reaction conditions, including light source, the concentration of feedstock, temperature, and pH, also affect the hydrogen production efficiency. Light is the energy source driving photocatalytic reactions. For laboratory evaluation of photocatalyst performance, solar simulator is often utilized as a convenient radiation source for photocatalytic reactions. Wavelength and light intensity, as two key parameters of light exhibit a significant impact on photocatalytic reactions. The selection of light spectral range is often based on the bandgap of the semiconductor as discussed in Section 3. For instance, TiO_2_ with a bandgap of ≈3.2 eV, can only be triggered by high‐energy ultraviolet light with wavelength less than 400 nm, which only accounts for about 5% of the sunlight. For relatively narrow bandgap semiconductors, such as CdS (2.40 eV) and g‐C_3_N_4_ (2.70 eV), their light absorption range can be extended from ultraviolet to visible light with a wavelength of 400–800 nm, accounting for ≈40% of the sunlight. Light intensity is defined as radiant power per surface area, and increasing light intensity with a suitable wavelength can raise the photocatalytic hydrogen production rate because the reaction is driven by incident photons to excite photocatalysts. For instance, the influence of light intensity on hydrogen production rate from methanol was investigated over Pt/TiO_2_ photocatalyst under near‐ultraviolet light irradiation. When the initial methanol concentration is low (3.0 mm), increasing light intensity from 0.5 to 2.3 mW cm^−2^ led to the achievement of an optimal hydrogen production rate, but further increasing the light intensity to 3.0 mW cm^−2^ did not significantly improve the hydrogen production rate.^[^ ^344^ ^]^ For methanol concentration of 10.0 mm as substrate, the maximum hydrogen production rate increases monotonically as the light intensity increases from 0.5 to 3.0 mW cm^−2^. The appropriate concentration of biomass or its derivatives can promote its accessibility with photocatalyst, which is beneficial for promoting the hydrogen production. In general, the hydrogen production rate is positively associated with the substrate concentration, because of the improved contact efficiency between photocatalyst and substrate. However, excessive substrate's concentration may reduce the photoreforming rate since excessive substrate's concentration may prevent the photocatalyst from light absorption. What is more, it is documented that the photocatalytic reaction rate can be also enhanced by increasing the reaction temperature. Typically, photocatalytic hydrogen production from carbohydrates was explored at a reaction temperature range from 40 to 80 °C.^[^ ^345^ ^]^ It was discovered that hydrogen production rate increased by nearly 50% when the reaction temperature was increased from 30 to 60 °C. It is possible that the increasing temperature not only promoted the diffusion and hydrolysis of the substrate, but also facilitated the H_2_ desorption. The pH value of the catalytic system also shows a significant impact on multiple aspects of biomass photoreforming by influencing the solubility of substrates, especially raw biomass with complex structure. What is more, the kinetics of light‐driven biomass reforming is associated with the pH value of the reaction system. Thereby, it affects the reaction.

## 8 Photocatalytic Production of Value‐Added Chemicals with Biomass as Green Hydrogen Resource

**Figure 23:**
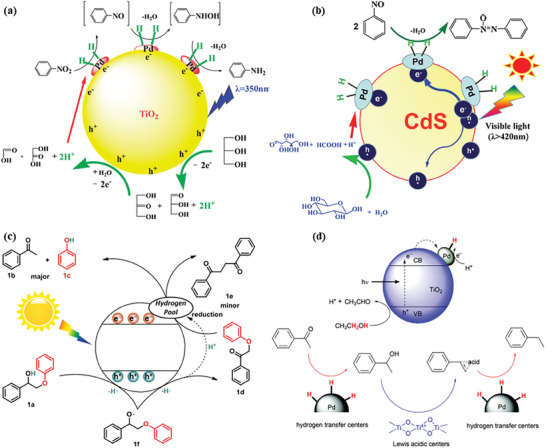
a) Schematic illustration of photocatalytic reduction of nitrobenzene to aniline coupled with oxidation of glycerol to fine chemicals over Pd/TiO_2_. Reproduced with permission. Reproduced with permission.^[^ ^349^ ^]^ Copyright 2015, The Royal Society of Chemistry. b) Schematic diagram of visible‐light‐driven simultaneous conversion of glucose to arabinose and nitrosobenzene to azoxybenzene over Pd/Meso CdS. Reproduced with permission.^[^ ^350^ ^]^ Copyright 2016, The Royal Society of Chemistry. c) Proposed mechanism for photoconversion of the lignin model via self‐hydrogen transfer hydrogenolysis. Reproduced with permission.^[^ ^352^ ^]^ Copyright 2017, American Chemical Society. d) Proposed mechanism for photocatalytic deoxygenation of aromatic ketones using ethanol as a hydrogen donor over Pd/TiO_2_ photocatalyst. Reproduced with permission. Reproduced with permission.^[^ ^353^ ^]^ Copyright 2020, The Royal Society of Chemistry.

Hydrogenation is one of the most efficient approaches to producing value‐added compounds in chemical industry, pharmaceutical industry, and so on. However, the conventional hydrogenation suffers from harsh reaction conditions (high temperature, high pressure_),_ extensive thermal input. Meanwhile, H_2_ for hydrogenation is frequently obtained from fossil resources with irreversible carbon emission. In some cases, transition metals (e.g., Fe) were even utilized as electron donor for hydrogenation, leading to the inevitable emission of environmental hazardous waste. Together, the conventional hydrogenation process is unsustainable. In sharp contrast, photocatalysis offers a thrilling hydrogenation technology.^[^ ^346^ ^]^ Because of the rich ‐OH and ‐CH*_x_* groups of biomass with high hydrogen content, biomass is a family of absolutely green and renewable hydrogen sources for unsaturated compounds hydrogenation, especially if powered by sunlight under ambient conditions.^[^ ^347^, ^348^ ^]^ Based on the aforementioned discussions, it is rationalized that, compared to hydrogen evolution from biomass, direct generation of value‐added products via hydrogenation using biomass as a green reactant shows a disruptive strategy for biomass valorization and green hydrogen utilization. In detail, utilizing biomass as a hydrogen resource for hydrogenation reactions presents a number of merits as follows: 1) Avoiding to use of high‐temperature and high‐pressure H_2_ gas and to generate eco‐unfriendly waste; 2) Use of inexhaustible sunlight as the energy source; 3) Green process under ambient conditions. 4) If rationally designed, simultaneous generation of desired products from biomass valorization and hydrogenation reaction can be achieved. Glycerol with poly‐hydroxyl structure, as the by‐product of biodiesel production, displays high hydrogen content and is a promising renewable hydrogen source for hydrogenation reaction. In our efforts, photoreduction of nitrobenzene to aniline and conversion of glycerol into high‐value‐added chemicals (e.g., glyceraldehyde, hydroxyacetic acid, and 1,3‐dihydroxyacetone) was cooperatively realized over Pd/TiO_2_ under ultraviolet light illumination.^[^ ^349^ ^]^ It was found that the mixing crystal phases of TiO_2_ displayed superior photocatalytic activity compared with the pure rutile or anatase with the descending order of Pt/P‐25 > Pt/TiO_2_ anatase > Pd/TiO_2_ rutile. The mixed crystal phase of P‐25 is more efficient for charge carrier separation without notable varied light absorption, thus benefiting the reaction. Meanwhile, the Pd nanoparticles (PdNPs) played a critical role in the reactions by not only acting as electron sinks for suppressing the recombination of the electron and hole pairs, but also provided active sites to generate H‐PdNPs species from glycerol for the hydrogenation of nitrobenzene (**Figure** **23**a). Photogenerated holes from P‐25 were considered as active species for glycerol oxidation to high‐value‐added chemicals including 1,3‐dihydroxyacetone, glyceraldehyde, formic acid, and hydroxyacetic acid. This work opens up a new way for the simultaneous transformation of glycerol to chemicals and the production of aniline from nitrobenzene with the only input of light energy under ambient conditions.

As a key building block of lignocellulose biomass, glucose was also employed as a green reductant for the photoreduction of nitrosobenzene to azoxybenzene over Pd‐decorated mesoporous CdS. Correspondingly, glucose was transformed into arabinose.^[^ ^350^ ^]^ In this photocatalytic process, mesoporous CdS was excited to generate electrons and holes under visible light (>420 nm) irradiation (Figure 23b). The holes of mesoporous CdS effectively oxidized glucose to arabinose and formic acid through C1‐C2 bond cleavage. The electrons captured by Pd nanoparticles from mesoporous CdS were consumed by H^+^ from water splitting and glucose depronation to form H‐PdNPs for the reduction of nitrosobenzene. The synergism of PdNPs and mesoporous CdS was responsible for achieving 92% selectivity of azoxybenzene and 70% selectivity of arabinose.

Lignin, as the exclusive natural aromatics, has received increasing research interest. Meanwhile, lignin also can be used as hydrogen source for hydrogenation because it contains massive hydroxyl and methoxy groups.^[^ ^351^ ^]^ Luo et. al. presented hydrogenolysis of lignin models and extracts into phenols driven by solar energy using itself as a hydrogen source and ZnIn_2_S_4_ as a photocatalyst.^[^ ^352^ ^]^ The ZnIn_2_S_4_ was excited to generate photo‐generated electrons and holes under light illumination. The photo‐generated holes oxidized the C_α_H‐OH in lignin models into C═O, and photogenerated electrons were used to transform protons into hydrogen species a “hydrogen pool” adsorbed on the surface of the catalyst (Figure 23c). The adsorbed hydrogen species enabled hydrogenolysis of the adjacent C_β_‐O bonds to cleave lignin models into two monomers. Overall 71–91% yields of phenols were realized by a transformation of lignin β‐O‐4 models. Furthermore, Li and co‐workers developed photocatalytic deoxygenation of lignin‐derived aromatic ketones to alkyl arenes using Pd/TiO_2_ as a photocatalyst.^[^ ^353^ ^]^ In this catalytic process, alcohols not only substitute for H_2_ as the green hydrogen donors but also serve as solvents. No hydrogen production during the process indicated that deoxygenation was realized through hydrogen transfer from alcohols. It was found that the optimal Pd/TiO_2_ catalyst contains abundant Lewis acidic sites. The synergism of the hydrogen transfer mechanism and Lewis acidic sites of Pd/TiO_2_ mainly contributed to the efficient deoxygenation of lignin‐derived aromatic ketones to alkyl arenes (Figure 23d).

## 9 Conclusion and Perspectives

In summary, biomass is a highly promising feedstock for green hydrogen supply owing to its merits of carbon neutrality, renewability, low cost, and earth abundance. Especially, the non‐edible lignocellulose, as the second most abundant hydrogen resource behind water on the planet, is a huge natural hydrogen reservoir and an ideal alternative to fossil fuels for powering the carbon‐neutral economy in the future. Among various methods, heterogeneous photocatalysis has demonstrated great potential in biomass reforming toward H_2_ with the only input of sunlight under ambient conditions. In this review, we have summarized the recent advances in light‐driven hydrogen production from biomass over a number of heterogeneous semiconductor‐based photocatalysts. Both advantages and disadvantages of different semiconductors and cocatalysts have been compared with representative examples. In spite of numerous achievements, a number of critical issues, e.g., low optical absorption, and severe charges recombination need to be well addressed. Meanwhile, even though the relatively low Gibbs free energy of biomass reforming enabled efficient utilization of solar energy for biomass reforming toward H_2_ compared to water splitting, there has been no virtual success because of the lack of rational photocatalytic architectures. More critically, the insoluble nature and stubborn molecular structure of raw biomass, especially the lignocellulose, pose extraordinary challenges for hydrogen production because of sluggish reaction kinetics and undesired selectivity. What is more, the energy and molecule mechanism of solar‐to‐H_2_ transformation during light‐driven reforming biomass has remained largely unknown. At last, although the simultaneous generation of value‐added chemicals from biomass reforming with H_2_ seems attractive, the achievement of desirable products with high efficiency and high selectivity remains an extraordinary challenge, which is associated with the unfavorable physical properties and complex chemical structure of raw biomass. Based on the current challenges of photocatalytic biomass reforming, the following points are recommended:

1) First of all, there is an urgent need to develop efficient and cost‐effective depolymerization methods for converting raw biomass or its derivative macromolecules from its native form into a more accessible form for accelerating photocatalytic hydrogen production. The main reason is that compared to the raw biomass with a complex network of chemical bonds, the basic units and derivatives, e.g., monosaccharides, bio‐derived alcohols, and organic acids, are highly favorable for photocatalytic reforming toward H_2_ due to their high solubility and high reactivity. However, the indirect method is a commonly used strategy for processing insoluble raw biomass, involving a two‐step reaction process, i.e., depolymerization of insoluble substrates, followed by photocatalytic reforming of the derived small molecules. Such a method suffers from inherent shortcomings of multistep reactions and complex purification processes with high cost and low conversion efficiency. Various pretreatment technologies have been developed to improve efficiency of photocatalytic hydrogen production from raw biomass, including physical (e.g., mechanical grinding, radiation), chemical (e.g., acid, base, ionic liquids dissolution), and biological methods. Considering their technical‐economic bottlenecks and efficiency challenges, low‐cost and efficient technologies for hydrolysis of raw biomass toward soluble building blocks are highly expected, especially without adverse environmental impact. Biological pretreatment is favored due to its merits of low energy consumption, mild conditions, and nonpolluting, but still suffers from shortcomings of time‐consuming and low efficiency. Bio‐coordinating different pretreatment methods (i.e., physical/chemical/physical–chemical method) can reduce pretreatment time and enhance efficiency, exhibiting enormous prospects in process efficiency, environmental sustainability, and economic viability.

Certainly, if appropriate photocatalysts that can directly break the complex chemical bond network of biomass for releasing hydrogen can be explored, it will make great sense for green biomass‐to‐hydrogen conversion. Attention should be paid to the emerging solvents i.e. ionic liquids by cooperating with the rational photocatalyst, which is efficient for improving the solubility of raw biomass and facilitating the subsequent conversion. Herein, it is worth noting conversion of raw biomass into liquid organic hydrogen carriers, such as methanol, ethanol, and formic acid, for on‐site hydrogen generation can offer an ideal solution for addressing the critical issues of H_2_ storage and transportation, which is a key bottleneck of hydrogen industry chain. With the emergence of biocatalysis innovations, mainly in the fields of enzyme catalysis and fermentation technology, new avenues have opened for the conversion of raw biomass into liquid organic hydrogen carriers. Thus, the coupling of biocatalysis and photocatalysis processes provides a promising synergistic alternative for facilitating hydrogen production from raw biomass.

2) Ongoing efforts are still needed for the rational design and deliberate synthesis of active and robust semiconductor‐based photocatalysts. It is at the core of photocatalytic reforming of biomass toward hydrogen. Up to date, most photocatalysts are limited by ineffective light harvesting due to a large bandgap, and severe recombination of photogenerated electrons and holes. For example, the most reported semiconductors, e.g., TiO_2_, ZnO, and ZrO_2_‐based photocatalysts can merely absorb ultraviolet light, thus fundamentally limiting the ceiling efficiency of solar‐to‐H_2_ conversion during photocatalytic biomass reforming. By energy band structure engineering, it is feasible to tailor the light absorption capacity of the semiconductors. The exploration of a suitable light absorber without significantly compromising redox potentials is critical to breaking the bottleneck of biomass‐to‐H_2_ conversion. From the perspective of maximizing solar utilization, the infrared light that accounts for 53% of solar energy should be not overlooked although it cannot excite most of the conventional semiconductors to produce energetic charges. However, the prominent photo‐thermal effect may facilitate the depolymerization of raw biomass. More importantly, the reaction barrier of hydrogen release from the basic units of biomass may be reduced by the synergetic effect of charge carriers and high localized temperature. It is highly desirable to uncover the underlying energy‐transformation and molecule‐evolving mechanism during photo‐thermo‐catalysis. Meanwhile, as an essential descriptor of the photocatalyst, stability should also be considered. At present, the lifetime of photocatalysts is far below the requirements of practical application, which is mainly due to severe photocorrosion. The exploration of revolutionary semiconductor materials for addressing the critical issues above is required. Of note, the significant advances in materials science and technology, e.g., molecular beam epitaxy, metal–organic chemical vapor deposition, atomic layer deposition, and so on provide highly controllable and powerful tools for designing and fabricating semiconductors and cocatalysts at atomic scale. Advanced computing methods such as machine learning have also appeared as next‐generation tools for assisting in the rational design of semiconductors and cocatalysts, and integrating them into photocatalysts.
3) The property–activity–selectivity relationship of the photocatalyst remains largely unknown. What is more, the majority of research is merely focused on the reduction half‐reaction. Yet the other half‐reaction, i.e., biomass oxidation is usually overlooked. At present, the oxidation half reaction is still not clear enough even though it plays a vital role in the efficiency and selectivity of photocatalytic biomass reforming. It is essential to uncover the reaction mechanism at the atomic level by cooperatively using advanced in situ spectroscopic characterization technologies and theoretical calculations. For example, Diffuse Reflectance Infrared Fourier Transform Spectroscopy (DRIFT) and Surface Enhanced Raman Spectroscopy (SERS), can work in synergy with electron paramagnetic resonance (EPR) spectroscopy to provide a better understanding of the formation of active species such as radicals and the evolving track of biomass and water molecule toward chemicals and H_2_ over the photocatalyst. Certainly, first‐principle theoretical calculation can also provide reaction mechanism information at the atomic level. In addition, the mediation and optimization of charge carriers behavior are expected to be conducted by various approaches with the assistance of time‐resolved photoluminescence spectroscopy, transient absorption spectroscopy, and so on. Herein, considering the high reactivity of radical species during photocatlaysis, it is necessary to explore rational routes for forming value‐added chemicals rather than mineralization. It is noted that biomass is not only a hydrogen reservoir but also a natural precursor of various high‐value chemicals because of its diverse organic framework and rich functional groups. By rationally designing the photocatalyst properties, such as bandgap, built‐in electric field, crystal phase, morphology, size, surface, and interface, it is promising to produce high‐value chemicals by the oxidation half‐reaction. This strategy enables sunlight‐driven biomass‐to‐H_2_ conversion more economically competitive and promising.

The significant gap between the current status of solar energy utilization and its immeasurable potential defines a thrilling imperative for the post‐fossil fuel era. In practice, high‐flux concentrated sunlight is highly beneficial for biomass‐to‐H_2_ conversion. At first, the concentrated sunlight can significantly improve the reaction by offering dense charge carriers and high localized surface temperature. The cooperation of charge carriers and heat shows great potential in breaking the bottleneck of solar‐powered biomass‐to‐H_2_ conversion. Notably, both photocatalyst usage and land occupancy can be significantly reduced, thus technologically and economically favoring the commercialization. As a matter of fact, concentrating sunlight can be easily achieved by using a simple and cheap optical lens system composed of resinous materials.

Life cycle assessment (LCA) offers a powerful tool for a comprehensive assessment of the environmental impacts of biomass‐to‐H_2_ conversion. First, the LCA assessment can quantify and compare the carbon footprint and other environmental impacts of hydrogen from solar‐powered biomass with those of other methods such as fossil fuels steam reforming and water electrolysis. What is more, the resource consumption and energy utilization efficiency involved in the production, collection, and distribution of hydrogen from biomass can be well assessed through LCA, aiming at providing a scientific basis for resource management and energy planning. In addition, solar‐powered biomass‐to‐H_2_ conversion has potential economic advantages. With the continuous development of solar technology and biomass conversion technology, the cost of hydrogen from biomass would be gradually reduced. In this case, the cost structure and competitiveness of hydrogen from solar‐powered biomass reforming can also be analyzed by LCA assessment to provide reference for investment decision and market development. However, a number of challenges need to be overcome prior to the real commercialization of solar‐powered biomass‐to‐H_2_ conversion. The sustainable generation, collection, and supply of biomass resources without compromising ecosystem and food security are paramount. Meanwhile, further development of efficient and durable biomass conversion technologies is highly required for stepping forward to practice. In addition, the emerging hydrogen storage and transportation technologies need to be well integrated with the biomass‐to‐H_2_ conversion process.

Overall, solar‐driven reforming of biomass toward hydrogen holds a grand promise for meeting the massive requirements of low‐carbon hydrogen in the post‐fossil fuel era. However, considering its extraordinary complexity, this is not a single disciplinary business, and numerous research efforts by the entire science community are required. The joint efforts by chemists, materials scientists, physicists, and so forth are believed to be greatly favored for advancing this critical research.

## Conflict of Interest

The authors declare no conflict of interest.

## Supporting information

Supporting Information
