# Supplementary material for: Solar‐Driven Biomass Reforming for Hydrogen Generation: Principles, Advances, and Challenges
Source: Adv Sci (Weinh). 2024 May 30;11(29):2402651. doi: 10.1002/advs.202402651 (PMC11304308; doi:10.1002/advs.202402651)
Supplement: Supplementary file 1 — Supporting Information [file ADVS-11-2402651-s001.docx]

**Supporting Information**

**Solar-Driven Biomass Reforming for Hydrogen** **Generation: Principles, Advances and Challenges**

Hu Pan, ^a,^^b^ Jinglin Li, ^b^ Yangang Wang, ^a,^* Qineng Xia, ^a^ Liang Qiu ^b^, Baowen Zhou ^b,^*

^a^ College of Biological, Chemical Science and Engineering, Jiaxing University, Jiaxing, Zhejiang 314001, China.

^b^ Key Laboratory for Power Machinery and Engineering of Ministry of Education, Research Center for Renewable Synthetic Fuel, School of Mechanical Engineering, Shanghai Jiao Tong University, 800 Dongchuan Road, Shanghai 200240, China.

**Corresponding Authors:**

E-mails: ygwang8136@zjxu.edu.cn (Yangang Wang); zhoubw@sjtu.edu.cn (Baowen Zhou)

**Table S1**. Band gaps of the widely studied semiconductors for photocatalysis.^1,2^

| Semiconductors | Band gap structure (PH = 7) | | |
| --- | --- | --- | --- |
|  | Conduction band | Valence band | Band gap (eV) |
| Ce_2_O_3_ | -0.50 | 1.90 | 2.40 |
| Cu_2_O | -0.28 | 1.92 | 2.20 |
| In_2_O_3_ | -0.62 | 2.18 | 2.80 |
| LaTi_2_O_7_ | -0.60 | 3.40 | 4.00 |
| NiO | -0.50 | 3.00 | 3.50 |
| SrTiO_3_ | -1.26 | 2.14 | 3.40 |
| Ta_2_O_5_ | -0.17 | 3.83 | 4.00 |
| TiO_2_ | -0.29 | 2.91 | 3.20 |
| ZnO | -0.31 | 2.89 | 3.20 |
| ZrO_2_ | -1.09 | 3.91 | 5.00 |
| CaFe_2_O_4_ | 0.16 | 2.09 | 1.93 |
| Y-Fe_2_O_3_ | -0.20 | 2.40 | 2.60 |
| Bi_4_Ti_3_O_12_ | -1.88 | 1.12 | 3.00 |
| K_4_Nb_6_O_17_ | -0.04 | 3.06 | 3.10 |
| Nb_2_O_5_ | 0.09 | 3.49 | 3.40 |
| Bi_2_MoO_6_ | -0.39 | 2.49 | 2.88 |
| BiVO_4_ | 0.46 | 2.86 | 2.40 |
| InVO_4_ | -0.50 | 1.50 | 2.00 |
| BaTiO_3_ | 0.08 | 3.38 | 3.30 |
| WO_3_ | -0.10 | 2.70 | 2.80 |
| α-Fe_2_O_3_ | 0.28 | 2.48 | 2.20 |
| CuO | -1.16 | 0.85 | 2.00 |
| Bi_2_O_3_ | 0.33 | 3.13 | 2.80 |
| Agln_5_S_8_ | -0.61 | 1.45 | 2.06 |
| SnS_2_ | -0.06 | 2.04 | 2.10 |
| ZnSe | -0.64 | 2.16 | 2.80 |
| CdS | -0.52 | 1.88 | 2.40 |
| Ce_2_S_3_ | -0.91 | 1.19 | 2.10 |
| CuInS_2_ | -0.44 | 1.06 | 1.50 |
| In_2_S_3_ | -0.80 | 1.20 | 2.00 |
| PbS | -0.56 | -0.07 | 0.49 |
| Sb_2_S_3_ | 0.22 | 1.94 | 1.72 |
| ZnS | -1.04 | 2.56 | 3.60 |
| CuIn_5_S_8_ | -0.41 | 0.85 | 1.26 |
| TaON | -0.40 | 2.10 | 2.50 |
| g-C_3_N_4_ | -1.30 | 1.40 | 2.70 |
| Ta_3_N_5_ | -0.75 | 1.35 | 2.10 |
| LaTiO_2_N | -0.90 | 1.20 | 2.10 |
| InN | 0.70 | 1.35 | 0.65 |
| GaSb | -0.45 | 0.20 | 0.65 |

Hydrogen production rate= The yield of hydrogen/ (weight of catalyst × reaction time)

According to the references^3^, the apparent quantum yield (AQY) for H_2_ evolution was estimated as AQY (%) = A× R/ I×100

where A, R, and I represent the reaction coefficient, the H_2_ evolution rate, and the rate of incident photons, respectively.

The apparent quantum efficiency (AQE) of H_2_ evolution was calculated using the following equation:

$$AQE=\frac{2 \times Number of evolved hydrogen molecules}{Number of incident photons}\times100\%$$

where the number of incident photons was measured using a light spectroradiometer.

**Supporting references:**

[S1] Q. Lu, Y. Yu, Q. Ma, B. Chen, H. Zhang, *Adv. Mater.* **2016**, *28*, 1917-1933.

[S2] Y. Xu, M. A. Schoonen, *Am. Mineral.* **2000**, *85*, 543-556.

[S3] T. Oshima, S. Nishioka, Y. Kikuchi, S. Hirai, K. I. Yanagisawa, M. Eguchi, Y. Miseki, T. Yokoi, T. Yui, K. Kimoto, K. Sayama, O. Ishitani, T. E. Mallouk, K. Maeda, *J. Am. Chem. Soc.* **2020**, *142*, 8412-8420.
